# The genome of the tegu lizard *Salvator merianae*: combining Illumina, PacBio, and optical mapping data to generate a highly contiguous assembly

**DOI:** 10.1093/gigascience/giy141

**Published:** 2018-11-27

**Authors:** Juliana G Roscito, Katrin Sameith, Martin Pippel, Kees-Jan Francoijs, Sylke Winkler, Andreas Dahl, Georg Papoutsoglou, Gene Myers, Michael Hiller

**Affiliations:** 1 Max Planck Institute of Molecular Cell Biology and Genetics, Pfotenhauerstr. 108, 01307, Dresden, Germany; 2 Max Planck Institute for the Physics of Complex Systems, Nöthnitzerstr. 38, 01187, Dresden, Germany; 3 Center for Systems Biology Dresden, Pfotenhauerstr. 108, 01307, Dresden, Germany; 4 BioNano Genomics, Towne Centre Drive Suite, 100, 92121, San Diego, CA, USA; 5 Center for Molecular and Cellular Bioengineering, Technische Universität Dresden, Fetscherstr. 105, 01307, Dresden, Germany

**Keywords:** genome assembly, reptiles, lizard, Salvator merianae, Illumina, PacBio, optical map, genome alignment, conserved non-coding elements, transposon

## Abstract

**Background:**

Reptiles are a species-rich group with great phenotypic and life history diversity but are highly underrepresented among the vertebrate species with sequenced genomes.

**Results:**

Here, we report a high-quality genome assembly of the tegu lizard, *Salvator merianae*, the first lacertoid with a sequenced genome. We combined 74X Illumina short-read, 29.8X Pacific Biosciences long-read, and optical mapping data to generate a high-quality assembly with a scaffold N50 value of 55.4 Mb. The contig N50 value of this assembly is 521 Kb, making it the most contiguous reptile assembly so far. We show that the tegu assembly has the highest completeness of coding genes and conserved non-exonic elements (CNEs) compared to other reptiles. Furthermore, the tegu assembly has the highest number of evolutionarily conserved CNE pairs, corroborating a high assembly contiguity in intergenic regions. As in other reptiles, long interspersed nuclear elements comprise the most abundant transposon class. We used transcriptomic data, homology- and *de novo* gene predictions to annotate 22,413 coding genes, of which 16,995 (76%) likely have human orthologs as inferred by CESAR-derived gene mappings. Finally, we generated a multiple genome alignment comprising 10 squamates and 7 other amniote species and identified conserved regions that are under evolutionary constraint. CNEs cover 38 Mb (1.8%) of the tegu genome, with 3.3 Mb in these elements being squamate specific. In contrast to placental mammal-specific CNEs, very few of these squamate-specific CNEs (<20 Kb) overlap transposons, highlighting a difference in how lineage-specific CNEs originated in these two clades.

**Conclusions:**

The tegu lizard genome together with the multiple genome alignment and comprehensive conserved element datasets provide a valuable resource for comparative genomic studies of reptiles and other amniotes.

## Introduction

Comparative whole-genome analyses are of great importance to understanding the evolutionary trajectory of different species. The increasing number of sequenced genomes from diverse animal groups deepens the power of such comparative analysis, resulting in novel insights into the origin and evolution of many of the shared and unique genomic features that characterize different species.

Squamate reptiles comprise a species-rich group of approximately 6,500 lizards, 3,700 snakes, and 200 amphisbaenian species [1]. However, this group is heavily under-represented among the vertebrate species with sequenced genomes, especially considering the great morphological, behavioral, and life history diversity in this group. The green anole, *Anolis carolinensi*s, was the first lizard to have the genome sequenced [2]. Since then, squamates have been gaining attention for their relevance in understanding vertebrate evolution, as well as for reptile-specific features that are of human interest, such as venom with medical implications and adhesive features of gecko feet. This interest resulted in the sequencing and assembly of additional squamate genomes. To date, nine snake species (*Boa constrictor*, Burmese python, two rattlesnakes, king cobra, garter snake, corn snake, and two vipers) and six lizards (green anole, two geckos, Asian glass lizard, dragon lizard, Chinese crocodile lizard) have assembled genomes [3–16].

**Figure 1:**
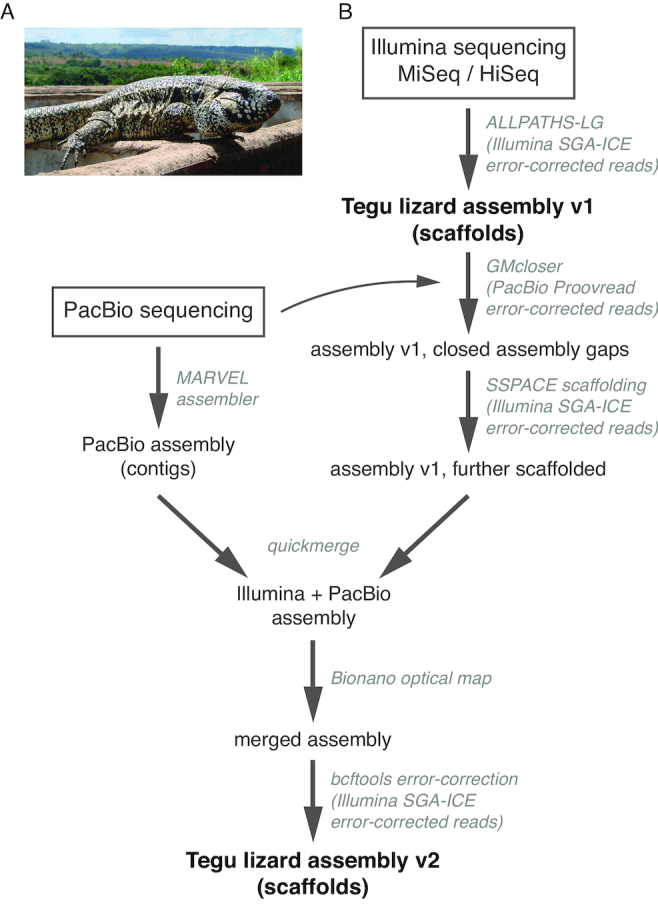
Workflow to generate the tegu lizard v2 assembly. **(A)** The tegu lizard, *Salvator merianae*. **(B)** Assembly v1 was built entirely from Illumina short-read data. To improve this assembly, we used Pacific Biosciences (PacBio) long-read data to close assembly gaps and extend scaffolds and merged the improved Illumina with a PacBio-only assembly. Finally, optical mapping data were used to resolve contig chimeras and scaffold even further. Used tools and their input data are shown in gray.

We extend the sampling of lizard species to the tegu lizard, *Salvator merianae* (Fig. 1A), a teiid lizard and the first representative of the Lacertoidea group with a sequenced genome. Tegus are large, omnivorous reptiles that are generally easy to keep in captivity and have economic importance in South America mainly for leather and meat, in addition to being sold as pets. The tegu lizard, native to South America, is widely distributed in open vegetation areas and also in forested landscapes [1, 17, 18]. Tegus are opportunistic and adapt well to many environments. Since it preys on crocodile, bird, and turtle eggs, tegus frequently become a threat to endangered species [19, 20].

Here, we generated Pacific Biosciences (PacBio) long-read and Bionano optical mapping data to substantially improve the quality of a previous short read-based tegu genome assembly [21]. We show that the new genome exhibits greatly increased contigs and scaffolds, making it the most contiguous reptile assembly to date. It also has the highest completeness of genes and conserved non-exonic elements (CNEs) compared to the genomes of other reptile species. We further provide repeat and gene annotations for this assembly. Finally, we generated a reptile-based multiple genome alignment comprising 10 squamates and 7 other amniote species and identified reptile-specific conserved genomic regions, together providing a valuable resource for comparative reptile genomics.

## Results

### Overview of the v2 tegu lizard assembly process

The first version of the tegu genome (v1) was assembled with ALLPATHS-LG [22] using high-coverage Illumina sequencing data (41X 2 × 300 bp MiSeq reads and 33X 2 × 150 bp HiSeq reads; Supplementary Table S1), resulting in a 2.026 Gb assembly with a scaffold N50 of 28.1 Mb (5,988 scaffolds) [21]. Despite the large number of scaffolds, the 36,428 contigs have an N50 value of only 175.8 Kb, and 80 Mb (4%) of the assembly consisted of assembly gaps. To upgrade this assembly, we generated 29.8X long sequencing reads with an N50 value of 8.4 Kb using the PacBio platform and corrected base errors in these reads using our Illumina data and the Proovread tool [23] (Supplementary Table S1). Next, we applied GMcloser [24] to close or shrink assembly gaps with these error-corrected PacBio reads. Since GMcloser also extends scaffold ends with the PacBio reads, which could provide new anchor points for Illumina mate-pair reads, we subsequently applied another round of scaffolding using our Illumina data and SSPACE [25]. Independently of using PacBio data to directly improve the Illumina assembly, we also assembled the PacBio reads into contigs with MARVEL [26, 27]. Because the read coverage of <25X after sequencing artifact correction in MARVEL's patch phase was lower than the minimum recommended 50X coverage to generate a PacBio-only assembly with high completeness [28, 29], we preferred to combine both Illumina and PacBio assemblies into a higher-quality hybrid assembly using quickmerge [30]. To further scaffold and resolve chimeric contigs, we used the Bionano system to generate a *de novo* optical map using molecules longer than 100 Kb. We found that 94.2% of the “quickmerged” assembly aligned to this optical map, showing that the optical map covered the genome well. We then combined the optical mapping and the quickmerged assembly into a final genome assembly and applied a last round of error correction using Illumina data. The workflow to generate the final v2 tegu assembly is illustrated in Fig. 1B.

The final v2 assembly of the tegu lizard genome has a size of 2.068 Gb, which is close to the 1.904–1.905 Gb size estimated by *k*-mer analysis of Illumina reads. The v2 assembly has scaffold N50/N90 values of 55.4/3.6 Mb and contig N50/N90 values of 521/79.7 Kb (Supplementary Table S2). Compared to the v1 assembly, contig N50/N90 values improved by 3/2.3 fold, and the number of bases in assembly gaps decreased 2.3-fold from 80 to 34.33 Mb.

### Comparing contiguity to other reptile assemblies

**Figure 2:**
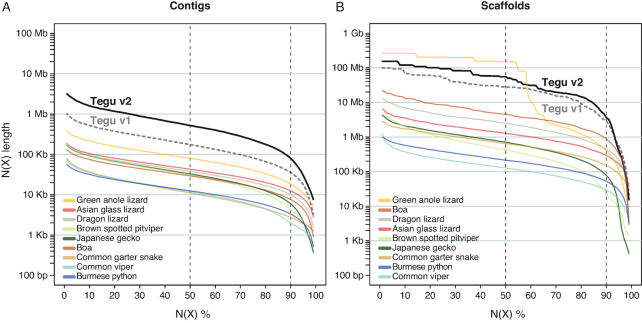
Comparison of assembly contiguity. N(x)% graphs show the contig **(A)** and scaffold **(B)** sizes (*y*-axis), where x% of the genome assembly consists of contigs and scaffolds of at least that size. The tegu lizard v1 and v2 assemblies are shown in gray and black. All other assemblies are sorted by the N50 values in the insets. Dashed lines mark the N50 and N90 values.

In comparison to other published squamate reptile genome assemblies, the v2 tegu assembly has the second-largest scaffold N50 value (Fig. 2A, Supplementary Table S2). Only the green anole lizard assembly (N50 of 150.6 Mb), which relied on fluorescence *in situ* hybridization of bacterial artificial chromosome clones to anchor scaffolds to chromosomes [2], has larger scaffolds. However, the v2 tegu assembly has a scaffold N90 value that is 9-times larger than that of the anole lizard (3.6 Mb vs 0.41 Mb). Furthermore, the v2 tegu assembly has the largest contig N50 and N90 values compared to all other assemblies (Fig. 2B, Supplementary Table S2), with a 6.5-times larger N50 value than the next best assembly (green anole lizard, 521 vs 80 Kb). Thus, the v2 tegu assembly represents the most contiguous reptile assembly at the moment.

### Comparing assembly completeness

**Figure 3:**
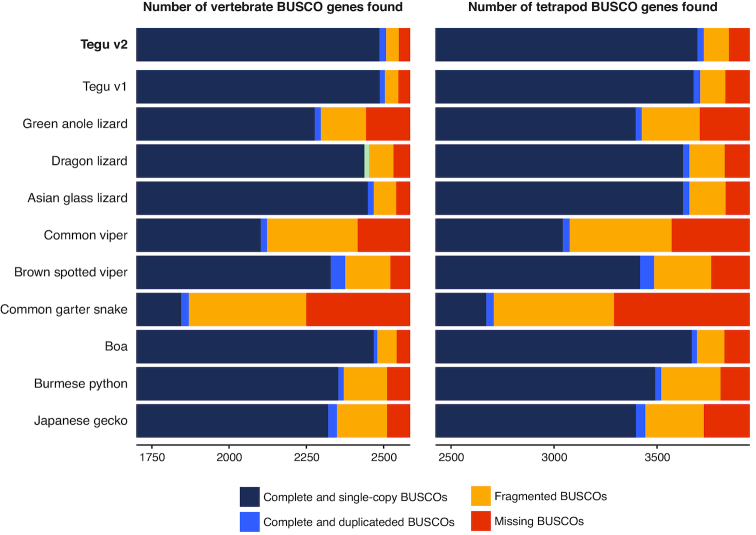
Comparison of genome completeness for coding genes. The bar charts show the number of complete, fragmented, and missing genes using two BUSCO datasets for vertebrate-conserved (left) and tetrapod-conserved (right) genes.

Next, we assessed whether the higher contiguity of the v2 tegu assembly is also reflected in higher completeness in functional genomic regions. First, we used Benchmarking Universal Single-Copy Orthologs (BUSCO) [31] to assess genome completeness for genes conserved in vertebrates (vertebrata database; 2,586 genes) and tetrapods (tetrapoda database; 3,950 genes). The v2 tegu genome has BUSCO completeness scores of 97% for the vertebrate gene set and 94.4% for the tetrapod gene set. This represents a slight improvement over the previous v1 assembly and higher scores compared to other reptile genomes (Fig. 3, Supplementary Table S3).

**Figure 4:**
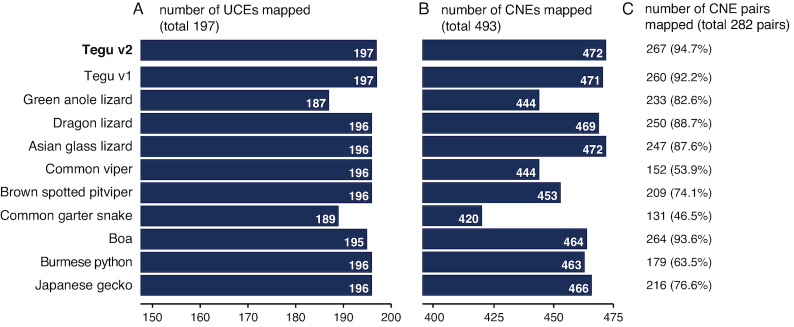
Using conserved non-coding elements to compare genome completeness and contiguity. Bar charts show **(A)** the number of aligning UCEs that do not overlap coding regions (N = 197 in total) and **(B)** the number of aligning CNEs that do not overlap exons (N = 493 in total). **(C)** The percentage of 282 evolutionarily conserved pairs of neighboring CNEs that are also found as neighbors in the squamate assemblies. Both UCE and CNE sets are highly conserved among vertebrates and thus are likely to exist in squamates.

Second, we assessed assembly completeness by quantifying the number of highly conserved non-exonic elements that can be found in the tegu genome and in the genomes of other reptiles. We first selected a set of 197 ultra-conserved elements (UCEs, originally defined as genomic regions longer than 200 bp that are identical between human, mouse, and rat [32]; Supplementary Table S4) that are also well conserved in chicken, zebrafish, and medaka. All 197 UCEs were identified in the tegu lizard genome (both v1 and v2 assemblies), while other reptile assemblies miss at least one of the UCEs (Fig. 4A). In addition, we selected a larger set of 493 vertebrate CNEs (Supplementary Table S5), defined as regions longer than 300 bp that are conserved among mammals, teleost fish, shark, and lamprey [33], and counted the number of elements that aligned to the genome of each species with at least 80% coverage and 60% identity. We found 472 CNEs (95.7%) in the v2 tegu lizard genome assembly and in the Asian glass lizard genome; all other reptile assemblies (including the tegu v1) contained fewer CNEs (Fig. 4B).

In addition, we used our CNE mapping data to assess assembly contiguity in intergenic regions by investigating conservation of CNE synteny. First, we defined a set of 282 pairs of neighboring CNEs that are located in the same chromosome and are at most 1 Mb apart from each other in three outgroup species (chicken, mouse, and human). Then, we asked how many of these pairs could be retrieved in the reptile assemblies. Since CNEs often overlap regulatory elements [34, 35] and maintain a conserved order with respect to their target genes and other CNEs, we expect that both CNEs in such a pair are also identified on the same scaffold in a well-assembled genome. In the v2 tegu assembly, we found 267 (94.7%) of these CNE pairs are located on the same scaffold and also at most 1 Mb apart from each other. The second-best assembly is the boa snake with 264 pairs (Fig. 4C). For the green anole lizard, only 233 pairs were found, likely because several CNEs align to shorter scaffolds or do not align at all. These results corroborate that the v2 tegu assembly also has a high completeness and contiguity in non-exonic regions, suggesting that this assembly is a valuable resource to study gene regulation in a reptile.

### Repeat content

**Figure 5:**
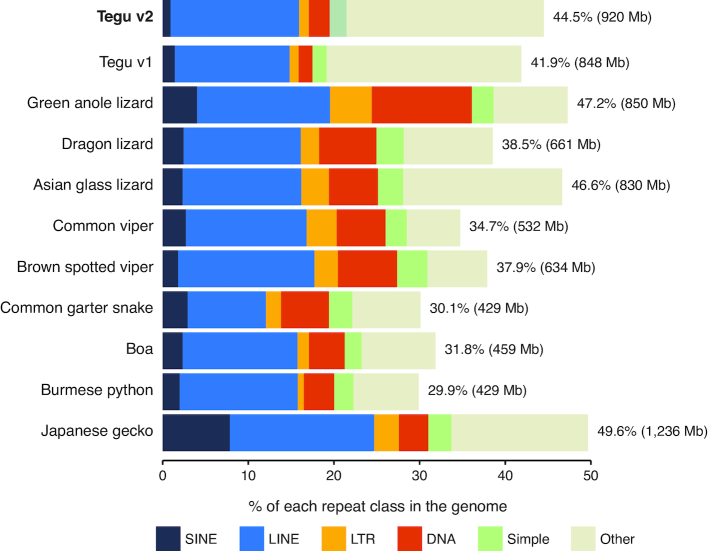
Repeat landscape in squamate genomes. Major classes of repeats are color coded and shown as bar charts that represent the portion of the genome they cover. Simple repeats comprise tandem repeats, low complexity regions, and satellite repeats.

To assess the repeat content of the tegu genome, we modeled and masked repeats in the v2 tegu lizard genome assembly using RepeatModeler and RepeatMasker. A high proportion of the genome (44.5%) was annotated as repeats, with long interspersed nuclear elements (LINEs) comprising the largest repeat class (Fig. 5). The v2 assembly contained 72 additional Mb in repeat-masked sequence that was not present in the v1 assembly. We also modeled and masked repeats in the other reptile genomes analyzed in this study and found a similar repeat content, with the exception of snakes that generally have fewer repeats (29%–38% vs 38%–50% for non-snake reptiles; Fig. 5, Supplementary Table S6).

### Tegu gene annotation

To annotate genes in the tegu lizard genome, we used MAKER [36] with four types of input data: transcriptome data, protein sequences from 33 sauropsid species (Supplementary Table S7), human genes mapped to the tegu lizard genome, and gene predictions based on the gene annotation of the v1 assembly. First, we used RNA sequencing (RNA-seq) data obtained from tegu lizard tissues [21] that we assembled to 304,367 transcripts. Second, we mapped sauropsid protein sequences available on UNIPROT to the tegu genome using exonerate [37], resulting in 3,637 high-quality homology-derived gene models. Third, we mapped human genes to the tegu genome using CESAR [38, 39], which resulted in 16,995 mappings that align, at least partially, to the tegu genome. Since CESAR was run on a genome alignment that makes extensive use of conserved alignment order, these 16,995 tegu loci likely contain orthologs of human genes. Fourth, we used BRAKER [40] to obtain gene predictions based on mapped RNA-seq data and the previous gene set from v1 assembly, resulting in 75,444 predictions after removing short, overlapping genes. The final gene set produced by MAKER contains 22,413 genes (BUSCO completeness score of 94.1%), which is within the range of the number of genes annotated in other lizards [2, 4, 12, 13, 16].

### Generating a resource for comparative reptile genomics

**Figure 6:**
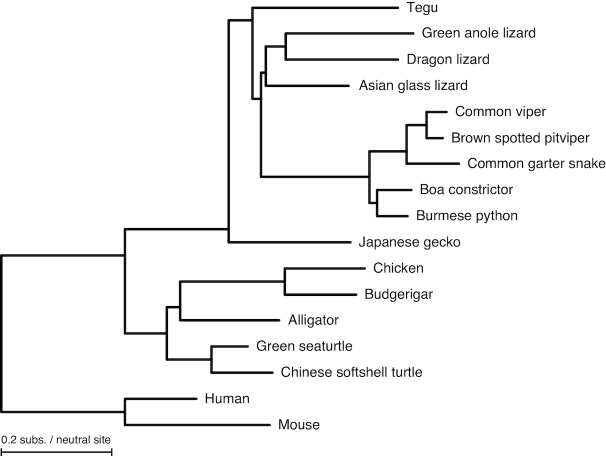
Phylogenetic tree of the amniote species included in our multiple genome alignment. The topology of the tree is based on [46] and [47]. Branch lengths represent the number of substitutions per neutral site, as estimated from four-fold degenerated codon positions.

To facilitate using the v2 tegu lizard assembly for comparative genomics, we generated a reptile-focused, highly sensitive, multiple-genome alignment. Since the tegu lizard genome is currently the most contiguous assembly, we used it as the reference. Our alignment includes 10 squamates and 7 other outgroup species such as mouse, human, birds, turtles, and alligator (Fig. 6, Supplementary Table S8). To detect genomic regions that are under evolutionary constraint, we integrated conserved regions detected by PhastCons [41] and GERP [42]. The multiple alignment, the conserved regions, and the tegu gene annotation are available at [43] and can be loaded into the University of California, Santa Cruz (UCSC) genome browser as an assembly hub [44] with the hub URL [45].

### CNE analysis

**Figure 7:**
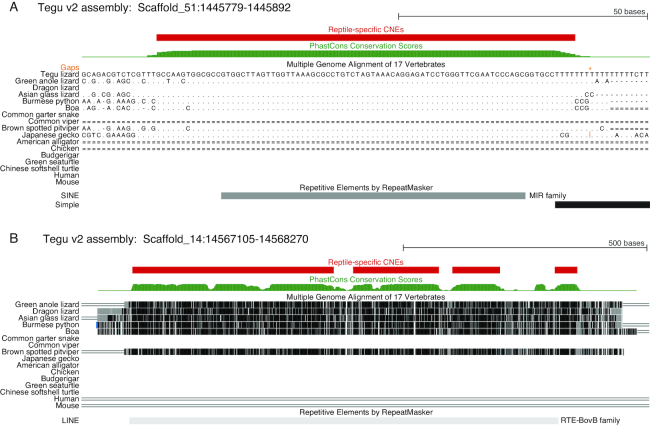
Transposon-derived conserved non-exonic (CNE) squamate-specific elements. **(A)** A squamate-specific CNE likely originated from the insertion of a short interspersed nuclear element (SINE) belonging to the Mammalian-wide interspersed repeats (MIR) family. The multiple genome alignment shows that this CNE is highly conserved among squamates but does not align to non-reptile species. **(B)** Several squamate-specific CNEs likely originated from the insertion of a LINE of the RTE-BovB family. This insertion likely happened after the split from the lineage leading to geckos as no sequence aligns to the gecko genome.

We intersected the conserved elements with our gene annotation to extract conserved regions that do not overlap exons. This resulted in 324,770 CNEs, covering 38 Mb (1.83% of the tegu genome). We further used our genome alignment to extract a CNE subset that is only conserved among squamates, resulting in 47,931 squamate-specific CNEs (3.3 Mb, 0.16% of the tegu genome). By intersecting squamate-specific CNEs with transposons, we found that only 146 of the 47,931 CNEs (0.3%) overlap transposons (Fig. 7). These 146 CNEs add up to 19.8 Kb, 86% of which overlap LINEs, consistent with this transposon class being the most abundant one in the assembly (Fig. 5). Overall, transposons may have given rise to only 0.6% (19.8 kb of 3.3 Mb) of the bases in squamate-specific CNEs.

## Discussion

Here, we present a high-quality genome assembly for the tegu lizard, the first sequenced Lacertoidea species. We combined Illumina short-read with PacBio long-read sequencing and Bionano optical mapping technologies to obtain an assembly with fewer gaps. In comparison to other available reptile genomes, the tegu v2 assembly has the longest contigs and thus the highest contiguity and also a higher completeness of genes and non-exonic elements. Furthermore, the new v2 assembly contains several additional megabases of repetitive sequences that were not present in the previous Illumina-based v1 assembly. This illustrates the ability of long PacBio reads to add repetitive sequence to a short-read assembly and thus increase the completeness in the repeat content of an assembly.

We found that all analyzed reptile genomes have a similar repeat composition, with LINEs making up the largest portion. Interestingly, even though the repeat content of reptile genomes is fairly high, very few of the squamate-specific CNEs overlap transposable elements. This contrasts with previous observations in mammals, where 16% of the placental mammal-specific CNEs overlap transposons [48]. Squamates are an older lineage compared to mammals (∼200 vs ∼100 million years ago), which makes the identification of ancient squamate-specific repeats more challenging. Nevertheless, the CNE-transposon overlap is more than 50-fold lower in squamates, which highlights a difference in how functional lineage-specific non-exonic elements evolved in these two clades.

We assessed and compared assembly completeness considering not only coding genes but also considering CNEs that often have regulatory activity. Furthermore, we used evolutionarily conserved pairs of CNEs as a novel measure of assembly contiguity. Together, this allows evaluating assembly completeness and contiguity in the non-exonic regions of an assembly. Since *cis*-regulatory elements typically reside in non-exonic regions, it is important to have a high completeness and contiguity in the intergenic portion of the genome, especially when applying high-throughput functional genomics methods, such as ChIP–seq or ATAC-seq, to discover regulatory elements.

To facilitate using the tegu genome for comparative studies focusing on the evolution of *cis*-regulatory elements, we generated a multiple genome alignment of nine other reptiles and seven other amniotes and annotated a comprehensive set of CNEs. The alignment and CNE sets provide a valuable resource for comparative reptile genomics and will help in our understanding of genome evolution in vertebrates.

## Methods

### DNA extraction and library preparation

DNA for Illumina and PacBio libraries was isolated after lysis of liver tissue in QIAgen Q2 lysis buffer with proteinase K and standard phenol-chloroform extraction. High-molecular-weight genomic DNA was precipitated by centrifugation after adding ice-cold ethanol and dissolved in Tris-EDTA, pH 8.0. All pipetting steps were carefully done with wide-bore pipetting tips to avoid any damage to the genomic DNA. RNA was removed by RNase A treatment. Pulse field gel electrophoresis (PFGE; SAGE Pippinpulse) showed that the resulting DNA molecules were between 50 and 200 Kb long.

Extraction of megabase genomic DNA for Bionano optical mapping was done according to the IrysPrep Animal Tissue protocol (Bionano Tech Note v. 1.1.12). Briefly, cell nuclei were isolated from embryonic tegu tissue and embedded in agarose plugs. After proteinase K and RNAse treatment of plugs, genomic DNA was extracted from agarose plugs and cleaned by drop dialysis against 1x TE. PFGE revealed DNA molecules with a minimum of 100 Kb and up to 1 Mb of length.

For transcriptome sequencing, we extracted total RNA from two tegu lizard embryos. Tissues were immediately frozen in liquid nitrogen, and total RNA was later extracted following a standard Trizol extraction.

### Sequencing

#### Illumina sequencing

Sequencing of the tegu genome with the Illumina platform is described in detail in [21]. Briefly, we sequenced 2 × 300 bp reads from three libraries on the MiSeq platform to a coverage of 41.3X and sequenced 2 × 150 bp reads from two 2 Kb mate-pair libraries and from two 10 Kb mate-pair libraries on the HiSeq 2500 to a coverage of 32.7X after adapter trimming. To obtain transcriptomic data to annotate genes, we sequenced 2 × 75 bp reads from eight strand-specific mRNA libraries on the Illumina HiSeq 2500 platform.

#### PacBio sequencing

Long insert libraries were prepared as recommended by PacBio according to the guidelines for preparing size-selected 20 Kb SMRTbel templates. Covaris g-Tubes were used for shearing 10 μg genomic DNA following the manufacturer's instructions to fragments sizes of 10 to 25 Kb. The PacBio SMRTbell library was size selected for fragments larger than 9 Kb making use of the SAGE BluePippin device. A second large insert library was prepared as described, but shearing of genomic DNA to 40 Kb fragments was done with the MegaRuptor device (Diagenode); this PacBio SMRTbell library was size selected for fragments larger than 10 Kb. A total of 205 single-molecule real-time sequencing (SMRT) cells were sequenced on the PacBio RSII instrument making use of P4 polymerase and C2 sequencing chemistry. Movie length was 3 hours for all SMRT cells.

#### Optical map

We delivered high-molecular-weight DNA embedded in agarose gel to the VIB Nucleomics Core. The purified DNA sequence-specific labeling was performed by the Nick, Labelling, Repair, and Staining steps according to IrysPrep TM NLRS assay (900 ng) version 30024D. Sequence specificity was provided by the nickase Nt.BspQ1 using a concentration between 5U and 7U. Labeling was carried out by a nick translation process in the presence of a fluorophore-labeled nucleotide. The labeled nicks were repaired to restore strand integrity, and DNA molecules were stained for visualization of the backbone visualization. The molecules were imaged using the Irys system, loading stained molecules automatically into Bionano Genomics nanochannel chips using electrophoresis. Label positions and lengths of DNA molecules were recorded by the on-board CCD camera using green and blue lasers in the Bionano Genomics Irys system. Data was generated from five flow cells.

### Genome assembly

#### Illumina-only assembly

We previously generated an assembly using only Illumina sequencing data [21]. Briefly, we used cutadapt (cutadapt, RRID:SCR_011841) [49] (v1.5) to trim adapters in the raw Illumina sequencing reads, iteratively corrected sequencing errors with the SGA-ICE pipeline [50], and assembled the error-corrected MiSeq and HiSeq reads using ALLPATHS-LG (ALLPATHS-LG, RRID:SCR_010742) [22] (v52188, parameters “*CLOSE_UNIPATH_GAPS = False HAPLOIDIFY = True*”). Details of this previous Illumina-only assembly are described in [21].

Next, we improved this Illumina assembly by closing gaps and further scaffolding using PacBio data generated for this study. First, we applied SOAP gapcloser (GapCloser, RRID:SCR_015026) [51] (v1.12, default parameters) with the SGA-ICE error-corrected MiSeq and HiSeq reads as input to resolve ambiguous base positions (Ns) that typically represent single-nucleotide polymorphisms. To correct sequencing errors in the PacBio reads, we used our SGA-ICE error-corrected MiSeq reads and Proovread [23] with the bwa mapper, first seeding with 12-mers and subsequently seeding with 13-mers. Then, we used GMcloser (GMcloser, RRID:SCR_000646) [24] (v1.5, parameter “*min_gap_size 200*”) with the error-corrected PacBio reads as input and the *–extend* parameter set to fill gaps and extend scaffold ends with aligning PacBio reads. This gap-closing step decreased the number of assembly gaps (runs of ≥25 Ns) from 28,792 to 11,628. Finally, we further scaffolded the scaffolds with extended ends with SSPACE (SSPACE, RRID:SCR_005056) [25] (v2.0, default parameters) and the SGA-ICE-corrected Illumina data.

#### PacBio assembly

Raw PacBio reads were assembled using the MARVEL assembler [26, 27] with default parameters unless mentioned otherwise. MARVEL consists of three major steps, namely, the setup phase, patch phase, and assembly phase. In the setup phase, reads were filtered by choosing only the best read of each ZMW and requiring subsequently a minimum read length of 2 Kb. The resulting 7.9 million reads (27.35X coverage) were stored in an internal database. The patch phase detects and corrects read artifacts including missed adapters, polymerase strand jumps, chimeric reads, and long low-quality read segments that are the primary impediments to long contiguous assemblies. The patched reads (24.6X coverage) were then used for the final assembly phase, which stitches short alignment artifacts resulting from bad sequencing segments within overlapping read pairs. This step is followed by repeat annotation and the generation of the overlap graph. To this end, we used the tool LAq with a quality cutoff of 35 to calculate a quality and a trim annotation track. In addition, alignments were forced through low-quality regions (<200 bp) that remained in the patched reads. LArepeat in coverage auto detection mode was used to create a repeat annotation track based on overlap coverage anomalies. The final assembled contigs are generated by touring the overlap graph. To correct base errors, we first used the correction module of MARVEL, which makes use of the final overlap graph and corrects only the reads that were used to build the contigs. Corrected contigs were further polished using PacBio's Quiver tool [52].

#### Merging Illumina and PacBio assemblies

We used quickmerge [30] to combine the improved Illumina and PacBio assemblies. Quickmerge was run in two rounds. In the first round, we used the improved Illumina assembly as query and the PacBio assembly as reference, specifying the “*-l*” parameter to the scaffold N50 of the reference assembly. In the second round, we again used the improved Illumina assembly as query but the resulting assembly from round 1 as reference (again setting the “*–l*” parameter to the N50 of the reference assembly).

#### Optical map

A genome map was assembled *de novo* and used to order and orient the scaffolds from the quickmerged Illumina-PacBio assembly and to correct contig misassemblies. Consensus physical maps (CMAPs) were assembled using Bionano Access 1.1.2 and Bionano Solve 3.2. Molecules were filtered for minimum length of 100 Kb, minimum of eight labels on each molecule, and a backbone intensity of maximum 0.45 (n = 835,772; approximately 89X raw coverage). A *P* value threshold for the optical mapping assembly was set to at least 1 × 10^−10^. A total of 2,742 CMAPs (N50 of 1.052 Mb; total CMAP length of 2,141.075 Mb) were generated.

#### Hybrid scaffolding

We used the Bionano Access 1.1.2/Bionano Solve 3.2 hybrid-scaffolding pipeline, with input parameters optimized for human (see Bionano Genomics “Hybrid Scaffolding Theory of Operation” for a detailed explanation and summary of all input parameters [53]). In short, the process of hybrid scaffolding includes alignment of the Illumina-PacBio assembly against the Bionano physical maps, identifying and resolving conflicting alignments, merging of non-conflicting assembly and CMAPs into hybrid scaffolds, and the final translation back to fasta format.

#### Final assembly polishing

To correct remaining base errors, we used the variant detector FreeBayes (FreeBayes, RRID:SCR_010761) [54] and bcftools consensus [55] with a score cutoff of 1 to detect and correct erroneous or polymorphic positions in the assembly. Of 6,322,937 assembly positions where the base identity was changed (0.3% of the genome), 82.8% correspond to heterozygous positions and 17.2% correspond to erroneous base calls in the original assembly.

#### Obtaining per-base quality values

We used bcftools (SAMtools/BCFtools, RRID:SCR_005227) [56] with the Illumina sequencing reads (parameters “*bcftools mpileup -A | bcftools call -c*”) to obtain a quality value for each base in the assembly where a read is mapped to. Overall, 99.8% of the bases in the tegu v2 assembly have a Phred quality score greater than 40, which corresponds to a base accuracy of 99.99%.

### *k*-mer size estimation

We used genomescope [57] to obtain a *k*-mer-based estimate of the size of the tegu genome. We used the Illumina sequencing reads and the default *k*-mer size of 21 bp and obtained a minimum-to-maximum estimate of 1.904 to 1.905 Gb.

### Comparison of the tegu v1 and v2 assemblies

To analyze the sequence similarity between both assemblies, we aligned the v2 assembly to the v1 assembly as described previously [58] but with lastz [59] parameters “*–gappedthresh = 8000 –hspthresh = 4000*”. Then we determined the sequence identity in all aligning regions (note that no comparison can be made in assembly gap regions that were closed in v2). This showed that 99.83% of the bases in the v1 assembly are unchanged in v2 and that both assemblies differ in 0.12% substitutions, 0.04% insertions, and 0.0028% deletions. Inspecting the differences and the aligned Illumina reads showed that almost all differences between both assemblies correspond to polymorphisms, where reads support both variants and the assembly polishing step changed identity of the variant.

### Transcriptome assembly

We first trimmed the raw sequencing reads for the presence of sequencing adapters with cutadapt (cutadapt, RRID:SCR_011841) [49] (v1.5), setting a minimum read length of 30 bp, and then mapped the trimmed reads against the tegu lizard genome using HISAT2 (HiSat2, RRID:SCR_015530) [60] (v2.1.0, parameters “*–rna-strandness RF*”). We assembled the mapped reads using Cufflinks (Cufflinks, RRID:SCR_014597) [61] (v2.2.1, parameters “*–library-type fr-firststrand*”), resulting in 63,127 transcripts, and also using Trinity (Trinity, RRID:SCR_013048) [62] (v2.3.2, parameters “*–SS_lib_type RF –genome_guided_max_intron 20000*”), resulting in 481,835 transcripts. Next, we applied PASA (PASA, RRID:SCR_014656) [63] (v.2.3.0, default parameters) to map the assembled transcripts to the genome with Basic Local Alignment Search Tool-like alignment tool. PASA also removed low-quality alignments (alignment identity less than 95% and minimum of 75% aligned) and combined both trinity and cufflinks transcripts by collapsing redundant transcripts and clustering transcripts that have overlapping exons on the same strand. This resulted in 304,367 transcripts.

### Assessing assembly completeness

We assessed completeness of the tegu lizard genome assembly and compared it to the genomes of other reptiles by quantifying both the number of conserved genes and non-exonic genomic regions found in each genome. For genes, we ran BUSCO (BUSCO, RRID:SCR_015008) [31] (v3.0.2) on genome mode to search for genes conserved in vertebrate and tetrapod species (vertebrata_odb9 and tetrapoda_odb9 gene databases, created on 2016–02-13). The vertebrata database consists of 2,586 genes, and the tetrapoda database consists of 3,950 genes.

We further assessed assembly completeness using two sets of non-exonic regions that are highly conserved among vertebrates. First, as previously described [26], we selected a set of 197 UCEs, which are genomic regions equal or greater than 200 bp that are identical between human, mouse, and rat [32] that are also conserved in chicken, zebrafish, and medaka and that do not overlap exons (based on human hg38 ensGene table from UCSC genome browser). Second, we obtained CNEs that are well conserved among mammals and teleost fish and also align to shark and lamprey from [33]. To ensure that CNEs can be easily found in a genome if the CNE sequence is present, we focused only on those CNEs that are longer than 300 bp. Furthermore, we removed 30 bp from both ends as often the CNE core is conserved among large evolutionary distances. This resulted in a set of 493 CNEs. Of these, 282 pairs of CNEs are neighbors located on the same chromosome and at most 1 Mb from each other in the human, mouse, and chicken genome and, thus, are evolutionarily conserved neighbors. Both UCE and CNE sets were mapped to the genome using lastz [59] (v1.02.00, parameters “*–gappedthresh = 3000 –hspthresh = 2500 –seed = match6 –format = general*”). We further filtered these mappings for ≥60% alignment identity and ≥80% alignment coverage. The UCE/CNE sequences are provided as fasta files at [43] as a resource for further vertebrate assembly completeness assessments.

### Repeat annotation

We used RepeatModeler (RepeatModeler, RRID:SCR_015027) [64] (v1.0.8, parameters “*-engine ncbi*”) to *de novo* identify repeat families in the tegu genome. Then, we used RepeatMasker (RepeatMasker, RRID:SCR_012954)(v4.0.5, default parameters) with the resulting repeat library to soft-mask the tegu genome and ran Tandem Repeat Finder [65] to annotate simple and tandem repeats. We applied the same procedure to the genomes of all other analyzed squamates.

### Gene annotation

In order to annotate genes in the tegu genome, we prepared the following four evidence-based datasets. First, we used our assembled PASA transcripts, which were passed to MAKER via the est_gff option in the maker_opts.ctl file. Second, we downloaded protein sequences available on UNIPROT (data accessed in March/April 2018; 20 lizard species, 9 snake species, chicken, softshell turtle, and two alligator species; Supplementary Table S7). We only kept those proteins with strong experimental evidence (sequences annotated with PE = 1 or PE = 2), resulting in 3,739 protein sequences. We mapped these sequences to the tegu v2 genome with exonerate [37] (v2.2.20, parameters “*-m protein2genome –subopt 0 -M 20 000 -D 2000 –minintron 20 –maxintron 50 000 –softmasktarget T –proteinhspdropoff 20 –exhaustive no –refine region –bestn 1*”). This resulted in 3,637 mappings for 3,607 proteins, which were passed to MAKER via the protein_gff option in the maker_opts.ctl file. Third, we mapped human genes to the tegu lizard genome with CESAR [38, 39]. We selected 20,145 transcripts corresponding to the longest isoform of human Ensembl genes downloaded from UCSC genome browser (hg38 ensGene table) and, based on our pairwise whole genome alignment (below), annotated exons with an intact open reading frame and consensus splice sites in the tegu lizard genome. We filtered out mappings corresponding to single-exon genes that were smaller than 100 bp and mappings spanning more than 10 Mb. This resulted in 16,995 mappings that were passed to MAKER via the model_gff option in the maker_opts.ctl file. Fourth, we ran BRAKER [40] with the HISAT2-mapped reads and the gene annotation of the v1 assembly version [21] as input. We filtered the 81,625 gene predictions from BRAKER to eliminate short, low-scoring overlapping genes, resulting in 75,444 predictions that were passed to MAKER via the pred_gff option in the maker_opts.ctl file. In addition to evidence-based datasets, we also used *de novo* gene prediction using Augustus [66] with a previously obtained gene model [21] and specified the MAKER augustus_species option in the maker_opts.ctl file.

We ran MAKER [36] (v2.31.9), setting *est2genome* and *protein2genome = 1, max_dna_len = 300 000, min_contig = 100, always_complete = 1, keep_preds = 0, split_hit = 10 000, single_exon = 1, single_length = 150, correct_est_fusion = 1*, and *alt_splice = 0*.

### Multiple genome alignment

First, we computed pairwise genome alignments between tegu and other reptiles and amniotes using the lastz/chain/net pipeline, as described in [33, 58]. To this end, we used lastz [59] (v1.04.00) with alignment parameters “*K = 2200 L = 3000 Y = 9400 H = 2000*” and the default scoring matrix for aligning reptile species to the tegu genome. The same parameters were used to align non-squamate species, except that we set *Y =*3,400 and used the HoxD55 scoring matrix. Next, we built co-linear alignment chains with axtChain [67] using default parameters and applied chainCleaner [68] (parameters *-LRfoldThreshold = 2.5 -doPairs -LRfoldThresholdPairs = 10 -maxPairDistance = 10 000 -maxSuspectScore = 100 000 -minBrokenChainScore = 75 000*) to improve alignment specificity. For non-squamate species, which are separated from the tegu by >0.72 neutral substitutions per site, we subsequently ran an additional round of highly sensitive local alignments with lastz to uncover additional alignments that were missed before. To this end, we used the parameters “*K = 1500 L = 2500 W = 5*” on all non-aligning regions flanked by local alignments in the chains that are between 20 bp and 100 Kb long. As shown in [33, 58], this procedure is able to uncover numerous additional alignments to exons and CNEs. All local alignments were quality-filtered by requiring that each alignment contain at least one ≥30 bp region with ≥60% sequence identity and ≥1.8 bits entropy as described in [33]. We then generated alignment nets from the chains using chainNet [67] with default parameters. We removed low-scoring alignment nets that are unlikely to represent real homologies by running a non-nested filtering procedure that keeps only nets that span ≥4 Kb in both genomes and have a score ≥20,000. Nets that represent inversions or local translocations and have a score ≥10,000 were also kept. Finally, we used Multiz [69] to produce a multiple alignment from all filtered pairwise alignment nets. The phylogenetic position of the squamate species was taken from [46]. We estimated neutral branch lengths in the phylogenetic tree using phyloFit [41] with parameters “*–EM –precision HIGH –subst-mod REV*” and 4-fold degenerated third codon positions based on our gene annotation.

### Annotating conserved regions

To detect genomic regions that are under evolutionary constraint, we applied PhastCons [41] (parameters “*expected-length = 45, target-coverage = 0.3 rho = 0.3*”) and GERP (GERP, RRID:SCR_000563) [42] (default parameters) to our multiple alignment using the phylogenetic tree with neutral branch lengths. We merged both PhastCons and GERP sets of conserved regions, joined those regions separated by ≤10 bp, and filtered the resulting ones for a minimum size of 30 bp. Finally, we only kept conserved regions that align well to at least four of the nine non-tegu squamates in the tegu-based alignment.

To obtain CNEs, we excluded all bases from the full set of conserved elements that overlap exons in our CESAR or MAKER gene annotation. Specifically, we subtracted exonic bases from all bases in conserved elements and required that the resulting CNEs are at least 30 bp long.

We defined two subsets of CNEs, a squamate-specific set and a not squamate-specific set, based on well-aligning regions in other species. For each species in the multiple alignment, we determined all windows of ≥30 bp where the alignment identity is ≥60%. To define the squamate-specific subset, we selected those CNEs that overlap these aligning windows in at least six of the nine squamates and not a single non-squamate amniote. To define the not squamate-specific subset, we selected those CNEs that overlap aligning windows in at least six of the nine squamates and overlap aligning windows in at least one non-squamate amniote. To determine the overlap between CNEs and transposons, we considered SINE, LINE, LTR, and DNA transposons from our RepeatMasker annotation and extracted CNEs that overlap transposons for at least 30 bp.

## Availability of supporting data

All raw sequencing data and genome assemblies are available at the National Center for Biotechnology Information under project accession number PRJNA473319. All other data, including annotated genes, the multiple genome alignment, and conserved element datasets, are available at https://bds.mpi-cbg.de/hillerlab/TeguGenomeData/. The genome and its annotations can also be loaded into the UCSC genome browser as an assembly hub (URL https://bds.mpi-cbg.de/hillerlab/TeguGenomeData/assemblyHub/hub.txt). Optical map, annotation, and tree data are also available from the *GigaScience* GigaDB repository [70].

## Additional files

SupplementaryTables.xlsx

## Abbreviations

BUSCO: Benchmarking Universal Single-Copy Orthologs; CMPA: consensus physical map; CNE: conserved non-exonic element; LINE: long interspersed nuclear element; PacBio: Pacific Biosciences; PFGE: pulse field gel electrophoresis; RNA-seq: RNA sequencing; SMRT: single-molecule real-time sequencing; UCE: ultra-conserved element.

## Competing Interests

The authors declare no competing interests.

## Ethics, consent, and permissions

All DNA samples were derived from a liquid nitrogen snap-frozen liver tissue sample from a single male individual of the tegu lizard, *Salvator merianae*, collected in the state of Mato Grosso, Brazil (specimen accession number LG2117), in accordance with the Brazilian environmental and scientific legislation, under the SISBIO (Sistema de Autorização e Informação em Biodiversidade, Instituto Chico Mendes de Conservação da Biodiversidade) license 30309–4.

## Funding

This work was funded by the Max-Planck Gesellschaft (Michael Hiller, Gene Myers), Klaus Tschira Stiffung (Gene Myers), and FAPESP (Juliana G. Roscito; 2012/013198 and 2012/23360). Author contributions. JGR, KS, and MH conceived the study. JGR, KS, MP, KJF and GP participated in genome assembly. SW and AD extracted DNA and performed sequencing. JGR annotated the genome. JGR and MH analysed the data. GM acquired major funding. JGR and MH wrote the paper.

## Supplementary Material

giga-d-18-00186_original_submission.pdf

giga-d-18-00186_revision_1.pdf

reviewer_1_report_(original_submission) -- Athanasia Tzika 6/6/2018 Reviewed

reviewer_2_report_(original_submission) -- Todd Castoe 6/11/2018 Reviewed

reviewer_2_report_revision_1 -- Todd Castoe 10/9/2018 Reviewed

reviewer_3_report_(original_submission) -- Guojie Zhang 6/26/2018 Reviewed

reviewer_3_report_revision_1 -- Guojie Zhang 10/18/2018 Reviewed

Supplemental Tables
