# Supplementary material for: The genome of the tegu lizard Salvator merianae: combining Illumina, PacBio, and optical mapping data to generate a highly contiguous assembly
Source: Gigascience. 2018 Nov 27;7(12):giy141. doi: 10.1093/gigascience/giy141 (PMC6304105; doi:10.1093/gigascience/giy141)
Supplement: giga-d-18-00186_original_submission.pdf [file giy141_giga-d-18-00186_original_submission.pdf]

GigaScience

The genome of the tegu lizard Salvator merianae: combining Illumina, PacBio, and optical mapping data to generate a highly contiguous assembly

--Manuscript Draft--

**Manuscript Number:**	GIGA-D-18-00186
**Full Title:**	The genome of the tegu lizard Salvator merianae: combining Illumina, PacBio, and optical mapping data to generate a highly contiguous assembly
**Article Type:**	Data Note
**Funding Information:**	Max-Planck-Gesellschaft — Dr. Michael Hiller<br>FAPESP (2012/01319-8, 2012/23360) — Dr. Juliana Roscito<br>Klaus Tschira Stiftung — Prof. Gene Myers
**Abstract:**	Background: Reptiles are a species-rich group with a great phenotypic and life history diversity, but are highly underrepresented among the vertebrate species with sequenced genomes.<br>Results: Here, we report the genome assembly of the tegu lizard Salvator merianae, the first lacertid with a sequenced genome. We combined Illumina short read, PacBio long read and optical mapping data to generate a high-quality assembly with a scaffold N50 value of 55.4 Mb. The contig N50 value of this assembly is 521 Kb, making it the most contiguous reptile assembly so far. We show that the tegu assembly has the highest completeness of coding genes and conserved non-exonic elements (CNEs) compared to other reptiles. Furthermore, the tegu assembly has the highest number of evolutionarily conserved CNE pairs, corroborating a high assembly contiguity in intergenic regions. Like in other reptiles, Long Interspersed Nuclear Elements (LINEs) comprise the largest transposon class. We used transcriptomics data, homology- and de novo gene predictions to annotate 19,101 coding genes, of which 14,636 (77%) likely have human orthologs as inferred by CESAR-derived gene mappings. Finally, we generated a reptile-focused multiple genome alignment comprising 10 squamates and 7 other amniote species and identified conserved regions that are under evolutionary constraint. Conserved non-exonic elements cover 38 Mb (1.8%) of the tegu genome, with 3.3 Mb in these elements being reptile-specific. Some of these reptile-specific CNEs, totalling 20 Kb, originated from inserted transposons; however, overall this evolutionary mechanism is rare.<br>Conclusions: The tegu lizard genome together with the multiple genome alignment and comprehensive conserved element datasets provides a valuable resource for comparative genomic studies of reptiles and other amniotes.
**Corresponding Author:**	Michael Hiller<br><br>GERMANY
**Corresponding Author Secondary Information:**	
**Corresponding Author's Institution:**	
**Corresponding Author's Secondary Institution:**	
**First Author:**	Juliana Roscito
**First Author Secondary Information:**	
**Order of Authors:**	Juliana Roscito<br>Katrin Sameith<br>Martin Pippel<br>Kees-Jan Francoijs<br>Sylke Winkler

Powered by Editorial Manager® and ProduXion Manager® from Aries Systems Corporation

	Andreas Dahl
	Georg Papoutsoglou
	Gene Myers
	Michael Hiller
**Order of Authors Secondary Information:**	
**Additional Information:**	
**Question**	**Response**
Are you submitting this manuscript to a special series or article collection?	No
**Experimental design and statistics**<br><br>Full details of the experimental design and statistical methods used should be given in the Methods section, as detailed in our Minimum Standards Reporting Checklist. Information essential to interpreting the data presented should be made available in the figure legends.<br><br>Have you included all the information requested in your manuscript?	Yes
**Resources**<br><br>A description of all resources used, including antibodies, cell lines, animals and software tools, with enough information to allow them to be uniquely identified, should be included in the Methods section. Authors are strongly encouraged to cite Research Resource Identifiers (RRIDs) for antibodies, model organisms and tools, where possible.<br><br>Have you included the information requested as detailed in our Minimum Standards Reporting Checklist?	Yes
**Availability of data and materials**<br><br>All datasets and code on which the conclusions of the paper rely must be either included in your submission or deposited in publicly available repositories (where available and ethically appropriate), referencing such data using a unique identifier in the references and in the “Availability of Data and Materials” section of your manuscript.	Yes

Powered by Editorial Manager® and ProduXion Manager® from Aries Systems Corporation

Have you have met the above requirement as detailed in our Minimum Standards Reporting Checklist?	

Manuscript
Click here to download Manuscript Manuscript.pdf

The genome of the tegu lizard *Salvator merianae*: combining Illumina, PacBio, and optical mapping data to generate a highly contiguous assembly

Juliana G. Roscito[1,2,3], Katrin Sameith[1,2,3], Martin Pippel[1,3], Kees-Jan Francoijs[4], Sylke Winkler[1], Andreas Dahl[5], Georg Papoutsoglou[4], Gene Myers[1,3] and Michael Hiller[1,2,3*]

[1] Max Planck Institute of Molecular Cell Biology and Genetics, Dresden, Germany

[2] Max Planck Institute for the Physics of Complex Systems, Dresden, Germany

[3] Center for Systems Biology Dresden, Germany

[4] BioNano Genomics, San Diego, USA

[5] Center for Molecular and Cellular Bioengineering, Technische Universität Dresden, Germany

* To whom correspondence should be addressed:

Michael Hiller

Computational Biology and Evolutionary Genomics, Max Planck Institute of Molecular Cell Biology and Genetics & Max Planck Institute for the Physics of Complex Systems, Dresden, Germany.

Tel: +49 351 210 2781

Fax: +49 351 210 1209

Email: hiller@mpi-cbg.de

Abstract

Background: Reptiles are a species-rich group with a great phenotypic and life history diversity, but are highly underrepresented among the vertebrate species with sequenced genomes.

Results: Here, we report the genome assembly of the tegu lizard *Salvator merianae*, the first lacertid with a sequenced genome. We combined Illumina short read, PacBio long read and optical mapping data to generate a high-quality assembly with a scaffold N50 value of 55.4 Mb. The contig N50 value of this assembly is 521 Kb, making it the most contiguous reptile assembly so far. We show that the tegu assembly has the highest completeness of coding genes and conserved non-exonic elements (CNEs) compared to other reptiles. Furthermore, the tegu assembly has the highest number of evolutionarily conserved CNE pairs, corroborating a high assembly contiguity in intergenic regions. Like in other reptiles, Long Interspersed Nuclear Elements (LINEs) comprise the largest transposon class. We used transcriptomics data, homology- and *de novo* gene predictions to annotate 19,101 coding genes, of which 14,636 (77%) likely have human orthologs as inferred by CESAR-derived gene mappings. Finally, we generated a reptile-focused multiple genome alignment comprising 10 squamates and 7 other amniote species and identified conserved regions that are under evolutionary constraint. Conserved non-exonic elements cover 38 Mb (1.8%) of the tegu genome, with 3.3 Mb in these elements being reptile-specific. Some of these reptile-specific CNEs, totalling 20 Kb, originated from inserted transposons; however, overall this evolutionary mechanism is rare.

Conclusions: The tegu lizard genome together with the multiple genome alignment and comprehensive conserved element datasets provides a valuable resource for comparative genomic studies of reptiles and other amniotes.

Introduction

Comparative whole-genome analyses are of great importance to understanding the evolutionary trajectory of different species. The increasing number of sequenced genomes from diverse animal groups deepens the power of such comparative analysis, resulting in novel insights into the origin and evolution of many of the shared and unique genomic features that characterize different species.

Among vertebrates, the representation of species with genomes available is largely biased towards birds, mammals and fish. Non-avian reptiles (squamates), i.e. lizards, snakes, and amphisbaenians, are heavily under-represented, especially considering the great morphological, behavioural, and life history diversity in this group. The green anole *Anolis carolinensi*s was the first lizard to have the genome sequenced [1]. Since then, squamates have been gaining attention for their relevance in understanding vertebrate evolution, as well as for reptile-specific features that are of human interest, such as venom with medical implications and adhesive features of gecko feet. This interest resulted in the sequencing of additional reptiles such as the geckos *Eublepharis macularis* [2] and *Gekko japonicus* [3], the Asian glass lizard *Ophisaurus gracilis* [4], the dragon lizard *Pogona vitticeps* [5], the Chinese crocodile lizard *Shinisaurus crocodilurus* [6], the snake *Boa constrictor* [7], the Burmese python *Python bivitattus* [8], the rattlesnakes *Crotalus horridus* [9] and *C. mitchelli phyrrus* [10], the king cobra *Ophiophagus hannah* [11], the common garter snake *Thamnophis sirtalis* [12], the corn snake *Pantheropis gutattus* [13], the brown spotted pitviper *Protobothrops mucrosquamatus* [14] and the common viper *Vipera berus berus* [15]. However, these sequencing efforts are biased, considering the number of extant snake and lizard species: nine out of the approximately 3,690 snake species have genomes sequenced, while only five out of the approximately 6,450 lizard species have been sequenced [16].

We extend the sampling of lizard species to the tegu lizard *Salvator merianae* (Figure 1A), a teiid lizard and the first representative of the Lacertoidea group with a sequenced genome. Tegus are large, omnivorous reptiles that are generally easy to keep in captivity and have economic importance in South America mainly for leather and meat, in addition to being sold as pets. The tegu lizard, native to South America, is widely distributed in open vegetation areas and also in forested landscapes [16-18]. Tegus are opportunistic and adapt well to many environments. Since it preys on crocodile, bird and turtle eggs, tegus frequently become a threat to both native and non-native endangered species [19, 20].

Here, we generated PacBio long-read and Bionano optical mapping data to substantially improve the quality of a previous short read-based tegu genome assembly. We show that the new genome exhibits greatly increased contigs and scaffolds, making it the most contiguous reptile assembly so far. It also has the highest completeness in genes and conserved non-exonic elements compared to the genomes of other reptile species. We further provide repeat and gene annotation for this assembly. Finally, we generated a reptile-based multiple genome alignment comprising 10 squamates and 7 other amniote species and identified reptile-specific conserved genomic regions, altogether providing a valuable resource for comparative reptile genomics.

Results

Overview of the v2 tegu lizard assembly process

The first version of the tegu genome (v1) was assembled with ALLPATHS-LG [21] using high-coverage Illumina sequencing data, resulting in a 2.026 Gb assembly with a scaffold N50 of 28,146 Mb (5,988 scaffolds) [22]. Despite the large scaffolds, the 36,428 contigs had an N50 value of only 175,755 Kb, and 80 Mb (4%) of the assembly consisted of assembly gaps. To upgrade this assembly, we generated 29.8X long sequencing reads with a mean length of 5.7

Kb and an N50 value of 8.4 Kb using the PacBio platform and corrected base errors in these reads using our Illumina data and the Proovread tool [23]. Next, we applied GMcloser [24] to close or shrink assembly gaps with these error-corrected PacBio reads. Since GMcloser also extends scaffold ends with the PacBio reads, which could provide new anchor points for Illumina mate-pair reads, we subsequently applied another round of scaffolding using our Illumina data and SSPACE [25]. Independent of using PacBio data to directly improve the Illumina assembly, we also assembled the PacBio reads into contigs with MARVEL [26, 27]. Because the read coverage of <25X after sequencing artefact correction in MARVEL's patch phase was lower than the minimum recommended 50x coverage to generate a PacBio-only assembly with high completeness [28, 29], we preferred to combine both Illumina and PacBio assemblies into a higher-quality hybrid assembly using quickmerge [30]. To further scaffold and resolve chimeric contigs, we used the Bionano system to generate a *de novo* optical map using molecules longer than 100 Kb. 94.2% of the 'quickmerged' assembly aligned to this optical map, showing that the optical map covered the genome well. We then combined the optical mapping and the quickmerged assembly into a final genome assembly, and applied a last round of error-correction using Illumina data. The workflow to generate the final v2 tegu assembly is illustrated in Figure 1B.

The final v2 assembly of the tegu lizard genome has scaffold N50/N90 values of 55.4/3.6 Mb and contig N50/N90 values of 521/79.7 Kb (Supplementary Table 1). Compared to the v1 assembly, contig N50/N90 values improved by 3/2.3 fold, and the number of bases in assembly gaps decreased 2.3-fold from 80 to 34.33 Mb.

Comparing contiguity to other reptile assemblies

In comparison to other published squamate reptile genome assemblies, the v2 tegu assembly has the second-largest scaffold N50 value (Figure 2A, Supplementary Table 1). Only the green anole lizard assembly (N50 of 150.6 Mb), which relied on fluorescence in-situ hybridization of bacterial artificial chromosome clones to anchor scaffolds to chromosomes [1], has larger

scaffolds. However, the v2 tegu assembly has a scaffold N90 value that is 9-times larger than that of the anole lizard (3.6 Mb vs. 0.41 Mb). Furthermore, the v2 tegu assembly has the largest contig N50 and N90 values compared to all other assemblies (Figure 2B, Supplementary Table 1), with a 6.5-times larger N50 than the next best assembly (green anole lizard, 521 vs 80 Kb). Thus, the v2 tegu assembly represents the most contiguous reptile assembly at the moment.

Comparing assembly completeness

Next, we assessed whether the higher contiguity of the v2 tegu assembly is also reflected in higher completeness in functional genomic regions. First, we used BUSCO [31] to assess genome completeness for genes conserved in vertebrates (vertebrata database; 2,586 genes) and in tetrapods (tetrapoda database; 3,950 genes). The v2 tegu genome has BUSCO completeness scores of 97% for the vertebrate gene set and 94.4% for the tetrapod gene set. This represents a slight improvement over the previous v1 assembly and higher scores compared to other reptile genomes (Figure 3, Supplementary Table 2).

Second, we assessed assembly completeness by quantifying the number of highly conserved non-exonic elements that can be found in the tegu genome and in the genomes of other reptiles. We first selected a set of 197 Ultra-Conserved Elements (UCEs, originally defined as genomic regions of ≥ 200 bp that are identical between human, mouse and rat [32]; Supplementary Table 3) that are also well-conserved in chicken, zebrafish, and medaka. All 197 UCEs were identified in the tegu lizard genomes (both v1 and v2 assemblies), while other reptile assemblies miss at least one of the UCEs (Figure 4A). In addition, we defined a larger set of 493 vertebrate-conserved non-exonic elements (CNEs; Supplementary Table 4) and counted the number of elements that aligned to the genome of each species with at least 80% coverage and 60% identity. We found 472 CNEs (95.7%) in the v2 tegu lizard genome assembly and in the Asian glass lizard genome; all other reptile assemblies (including the tegu v1) contained less CNEs (Figure 4B).

In addition, we used our CNE mapping data to assess assembly contiguity in intergenic regions by investigating conservation of CNE synteny. We first defined a set of 282 pairs of neighbouring CNEs that are located in the same chromosome and are at most 1 Mb from each other in 3 non-reptile species (chicken, mouse, and human), and then asked how many of these pairs could be retrieved in the reptile assemblies. Since CNEs often overlap regulatory elements [33, 34] and maintain a conserved order with respect to their target genes and other CNEs, we expect that both CNEs in such a pair are also identified on the same scaffold in a well-assembled genome. In the v2 tegu assembly, we found 267 (94.7%) of these CNE pairs as pairs located in the same scaffold and also at most 1 Mb from each other. The second-best assembly is the boa snake with 264 pairs (Figure 4C). For the green anole lizard only 233 pairs were found, likely because several CNEs align to shorter scaffolds or do not align at all. These results corroborate that the v2 tegu assembly also has a high completeness and contiguity in non-exonic regions, suggesting that this assembly is a valuable resource to study gene regulation in a reptile.

Repeat content

To assess the repeat content of the tegu genome, we modelled and masked repeats in the v2 tegu lizard genome assembly using RepeatModeler and RepeatMasker. A high proportion of the genome (44.5%) was annotated as repeats, with Long Interspersed Nuclear Elements (LINEs) comprising the largest repeat class (Figure 5). The v2 assembly contained 72 additional Mb in repeat-masked sequence that was not present in the v1 assembly, consistent with the ability of long PacBio reads to add repetitive sequence to a short-read assembly. We also repeat modelled and masked the other reptile genomes analysed in this study and found a similar repeat content, with the exception of snakes that generally have less repeats (29-38% vs. 38-50% for non-snake reptiles; Figure 5, Supplementary Table 5).

Tegu gene annotation

To annotate genes in the tegu lizard genome we used MAKER [35] with three types of input data: transcriptome data, protein sequences from closely-related species, and human genes mapped to the tegu lizard genome. First, we used RNA-seq data obtained from tegu lizard tissues [22] that we assembled to 304,367 transcripts. Second, we mapped reptile protein sequences available on UNIPROT to the tegu genome using exonerate [36], resulting in 3,637 high-quality homology-derived gene models. Third, we mapped human genes to the tegu genome using CESAR [37], which resulted in 14,636 human genes that align at least partially to the tegu genome. Since CESAR was run on a genome alignment that makes extensive use of conserved alignment order, these 14,636 tegu loci likely contain orthologs of human genes. The final gene set produced by MAKER contains 19,128 transcripts for 19,101 genes.

Generating a resource for comparative reptile genomics

To facilitate using the v2 tegu lizard assembly for comparative genomics, we generated a reptile-focused, highly sensitive, multiple genome alignment, using the tegu lizard genome as the reference. Our alignment includes 10 squamates and 7 other outgroup species such as mouse, human, birds, turtles, and alligator (Figure 6, Supplementary Table 6). To detect genomic regions that are under evolutionary constraint, we integrated conserved regions detected by PhastCons [38] and GERP [39]. The multiple alignment, the conserved regions, and the tegu gene annotation are available at https://bds.mpi-cbg.de/hillerlab/TeguGenomeData/.

CNE analysis

We intersected the conserved elements with our gene annotation to extract conserved regions that do not overlap exons. This resulted in 324,770 CNEs, covering 38 Mb (1.83% of the tegu genome). We further used our genome alignment to extract a CNE subset that is only conserved among reptiles, resulting in 47,931 reptile-specific CNEs (3.3 Mb, 0.16% of the tegu genome). By intersecting reptile-specific CNEs with transposons, we found that 146 CNEs totalling 19.8 Kb (0.6% of 3.3 Mb) originate from transposons (Figure 7). LINEs contribute the

most of these CNEs (17 Kb), consistent with LINEs being the most abundant transposon class (Figure 5). Overall, this shows that transposons are also a source of reptile-specific CNEs, even though this appears to be a rare mechanism, consistent with results in mammals [40].

Discussion

Here we present a high-quality genome assembly for the tegu lizard, the first sequenced Lacertoidea species. We combined Illumina short read with PacBio long read sequencing and Bionano optical mapping technologies to obtain an assembly with fewer gaps and a higher completeness of genes, non-exonic elements, and repeats. In comparison to other available reptile genomes, the tegu v2 assembly has the longest contigs and thus the highest contiguity. We found that all analysed reptile genomes have a similar repeat composition with LINEs making up the largest portion. Our analysis also suggests that the repeat content of the green anole lizard (47%) is higher than the previously reported ~30% [1].

We assessed and compared assembly completeness not only considering coding genes, but also conserved non-exonic elements that often have regulatory activity. Furthermore, we used evolutionarily conserved pairs of CNEs as a novel measure of assembly contiguity. Together, this allows to evaluate assembly completeness and contiguity in the non-exonic regions that are important for studying gene regulation via high-throughput methods such as ChIP-seq or ATAC-seq.

To facilitate using the tegu genome for comparative studies that include gene regulation, we generated a multiple genome alignment covering 9 other reptiles and 7 other amniotes and annotated a comprehensive set of conserved non-exonic elements. The alignment and CNE sets provide a valuable resource for comparative reptile genomics.

Methods

Ethics, consent and permissions

All DNA samples were derived from a liquid nitrogen snap-frozen liver tissue sample from a single male individual of the tegu lizard *Salvator merianae*, collected in the state of Mato Grosso, Brazil (specimen accession number LG2117), in accordance with the Brazilian environmental and scientific legislation, under the SISBIO (Sistema de Autorização e Informação em Biodiversidade, Instituto Chico Mendes de Conservação da Biodiversidade) license 30309-4.

DNA extraction and library preparation

DNA for Illumina and PacBio libraries was isolated after lysis of liver tissue in QIAgen Q2 lysis buffer with Proteinase K and standard phenol-chloroform extraction. High molecular weight genomic DNA was precipitated by centrifugation after adding ice-cold Ethanol and dissolved in Tris-EDTA, pH 8.0. All pipetting steps were carefully done with wide bore pipetting tips to avoid any damage to the genomic DNA. RNA was removed by RNase A treatment. Pulse field gel electrophoresis (PFGE, SAGE Pippinpulse) showed that the resulting DNA molecules were between 50 and 200 Kb long.

Extraction of megabase genomic DNA for Bionano optical mapping was done according to the IrysPrep™ Animal Tissue protocol (Bionano Tech Note v. 1.1.12). Briefly, cell nuclei were isolated from embryonic tegu tissue and embedded in agarose plugs. After Proteinase K and RNAse treatment of plugs, genomic DNA was extracted from agarose plugs and cleaned by drop dialysis against 1x TE. PFGE revealed DNA molecules with a minimum of 100 Kb and up to 1 Mb of length.

For transcriptome sequencing, we extracted total RNA from two tegu lizard embryos. Tissues were immediately frozen in liquid nitrogen and total RNA was later extracted following a standard Trizol extraction.

Sequencing

Illumina sequencing

Sequencing of the tegu genome with the Illumina platform is described in detail in [22]. Briefly, we sequenced 2x300 bp reads from three libraries on the MiSeq platform to a coverage of 40X, and sequenced 2x150 bp reads from two 2 Kb mate-pair libraries and from two 10 Kb mate-pair libraries on the HiSeq 2500 to a coverage of 65X. To obtain transcriptomic data to annotate genes, we sequenced 2x75 bp reads from eight strand-specific mRNA libraries on the Illumina HiSeq 2500 platform.

PacBio sequencing

Long insert libraries were prepared as recommended by Pacific Biosciences according to the Guidelines for preparing size-selected 20 Kb SMRTbell™ templates. Covaris g-Tubes™ were used for shearing 10 ug genomic DNA following the manufacturer instructions to fragments sizes of 10 to 25 Kb. The PacBio SMRTbell™ library was size selected for fragments larger than 9 Kb making use of the SAGE BluePippin™ devise. A second large insert library was prepared as described, but shearing of genomic DNA to 40 Kb fragments was done with the MegaRuptor™ device (Diagenode) and this PacBio SMRTbell™ library was size selected for fragments larger than 10 Kb. A total of 205 SMRT cells were sequenced on the PacBio RSII instrument making use of P4 polymerase and C2 sequencing chemistry. Movie length was 3 hours for all SMRT cells.

Optical map

We delivered high molecular weight DNA embedded in agarose gel to the VIB Nucleomics Core. The purified DNA sequence-specific labelling was performed by the Nick, Labelling,

Repair and Staining steps according to IrysPrep TM NLRS assay (900 ng) version 30024D. Sequence specificity was provided by the nickase Nt.BspQ1 using a concentration between 5U and 7U. Labelling was carried out by a nick translation process in the presence of a fluorophore-labelled nucleotide. The labelled nicks were repaired to restore strand integrity and DNA molecules were stained for visualization of the backbone visualization. The molecules were imaged using the Irys system, loading stained molecules automatically into Bionano Genomics nanochannel chips using electrophoresis. Label positions and lengths of DNA molecules were recorded by the on-board CCD camera using green and blue lasers in the Bionano Genomics Irys system. Data was generated from a total of 5 flow cells.

Genome assembly

Illumina-only assembly

We previously generated an assembly using only Illumina sequencing data. Briefly, we used cutadapt [41] (v1.5) to trim adapters in the raw Illumina sequencing reads, iteratively corrected sequencing errors with the SGA-ICE pipeline [42], and assembled the error-corrected MiSeq and HiSeq reads using ALLPATHS-LG [21] (v52188, parameters '*CLOSE_UNIPATH_GAPS=False HAPLOIDIFY=True*'). Details of this previous Illumina-only assembly are described in [22].

Next, we improved this Illumina assembly by closing gaps and further scaffolding using PacBio data generated for this study. We first applied SOAP gapcloser [43] (v1.12, default parameters) with the SGA-ICE error-corrected MiSeq and HiSeq reads as input to resolve ambiguous base positions (N's) that typically represent SNPs. To correct sequencing errors in the PacBio reads, we used our SGA-ICE error-corrected MiSeq reads and Proovread [23] with the bwa mapper, first seeding with 12-mers and subsequently seeding with 13-mers. Then, we used GMcloser [24] (v1.5, parameter '*min_gap_size 200*') with the error-corrected PacBio reads as input and the *--extend* parameter set to fill gaps and extend scaffold ends with aligning PacBio reads. This gap closing step decreased the number of assembly gaps (runs of ≥25 N's) from 28,792

to 11,628. Finally, we further scaffolded the scaffolds with extended ends with SSPACE [25] (v2.0, default parameters) and the SGA-ICE corrected Illumina data.

PacBio assembly

Raw PacBio reads were assembled using the MARVEL assembler [26, 27] with default parameters unless mentioned otherwise. MARVEL consists of three major steps, namely the setup phase, patch phase and the assembly phase. In the setup phase, reads were filtered by choosing only the best read of each ZMW and requiring subsequently a minimum read length of 2 Kb. The resulting 7.9 million reads (27.35X coverage) were stored in an internal database. The patch phase detects and corrects read artefacts including missed adapters, polymerase strand jumps, chimeric reads and long low-quality read segments that are the primary impediments to long contiguous assemblies. The patched reads (24.6X coverage) were then used for the final assembly phase, which stitches short alignment artefacts resulting from bad sequencing segments within overlapping read pairs. This step is followed by repeat annotation and the generation of the overlap graph. To this end, we used the tool LAq with a quality cutoff of 35 to calculate a quality and a trim annotation track. In addition, alignments were forced through low quality regions (<200 bp) that remained in the patched reads. LArepeat in coverage auto detection mode was used to create a repeat annotation track based on overlap coverage anomalies. The final assembled contigs are generated by touring the overlap graph. To correct base errors, we first used the correction module of MARVEL, which makes use of the final overlap graph and corrects only the reads that were used to build the contigs. Corrected contigs were further polished using PacBio's Quiver tool [44].

Merging Illumina and PacBio assemblies

We used quickmerge [30] to combine the improved Illumina and PacBio assemblies. Quickmerge was run in two rounds. In the first round, we used the improved Illumina assembly as query and the PacBio assembly as reference, specifying the '*-l*' parameter to the scaffold N50 of the reference assembly. In the second round, we again used the improved Illumina

assembly as query but the resulting assembly from round 1 as Reference (again setting the '–l' parameter to the N50 of the reference assembly).

Optical map

A genome map was assembled *de novo* and used to order and orient the scaffolds from the quickmerged Illumina-PacBio assembly, and to correct contig misassemblies. Consensus physical maps (CMAP) were assembled using Bionano Access 1.1.2 and Bionano Solve 3.2. Molecules were filtered for minimum length of 100 Kb, minimum of eight labels on each molecule, and a backbone intensity of maximum 0.45 (n = 835772; approximately 89X raw coverage). A P-value threshold for the optical mapping assembly was set to at least $1x10^{-10}$. A total of 2,742 CMAPs (N50 of 1.052 Mb; total CMAP length of 2141.075 Mb) were generated.

Hybrid scaffolding

We used the Bionano Access 1.1.2/Bionano Solve 3.2 hybrid-scaffolding pipeline, with input parameters optimized for human (see Bionano Genomics 'Hybrid Scaffolding Theory of Operation' for a detailed explanation and summary of all input parameters [45]). In short, the process of hybrid scaffolding includes alignment of the Illumina-PacBio assembly against the Bionano physical maps, identifying and resolving conflicting alignments, merging of non-conflicting assembly and CMAPs into hybrid scaffolds, and the final translation back to fasta format.

Final assembly polishing

To correct remaining base errors, we used the variant detector FreeBayes [46] to detect polymorphic positions and fixed erroneous non-polymorphic sites in the reference sequence using bcftools consensus [47].

Transcriptome assembly

We first trimmed the raw sequencing reads for the presence of sequencing adapters with cutadapt [41] (v1.5), setting a minimum read length of 30 bp, and then mapped the trimmed reads against the tegu lizard genome using HISAT2 [48] (v2.1.0, parameters '*--rna-strandness RF*'). We assembled the mapped reads using Cufflinks [49](v2.2.1, parameters '*--library-type fr-firststrand*'), resulting in 390,275 transcripts, and also using Trinity [50](v2.3.2, parameters '*--SS_lib_type RF --genome_guided_max_intron 20000*'), resulting in 481,835 transcripts. Next, we applied PASA [51](v.2.3.0, default parameters) to map with BLAT the assembled transcripts to the genome, remove low-quality alignments (alignment identity less than 95% and minimum of 75% aligned), and combine both trinity and cufflinks transcripts by collapsing redundant transcripts and clustering overlapping transcripts. This resulted in 304,367 transcripts.

Assessing assembly completeness

We assessed completeness of the tegu lizard genome assembly and compared it to the genomes of other reptiles by quantifying both the number of conserved genes and non-exonic genomic regions found in each genome. For genes, we ran BUSCO [31](v3.0.2) on genome mode to search for genes conserved in vertebrate and tetrapod species (vertebrata_odb9 and tetrapoda_odb9 gene databases, created on 2016-02-13). The vertebrata database consists of 2,586 genes, and the tetrapoda database consists of 3,950 genes.

We further assessed assembly completeness using two sets of non-exonic regions that are highly conserved among vertebrates. First, as previously described [26], we selected a set of 197 Ultra-Conserved Elements (UCEs; genomic regions equal or greater than 200 bp that are identical between human, mouse and rat [32] that are also conserved in chicken, zebrafish, and medaka and that do not overlap exons (based on human hg38 ensGene table from UCSC genome browser). Second, we obtained CNEs that are well conserved among mammals and teleost fish, and also align to shark and lamprey from [52]. To ensure that CNEs can be easily found in a genome if the CNE sequence is present, we focused only on those CNEs that are

longer than 300 bp. Furthermore, we removed 30 bp from both ends as often the CNE core is conserved among large evolutionary distances. This resulted in a set of 493 CNEs. Of these, 282 pairs of CNEs are neighbours located on the same chromosome and at most 1 Mb from each other in the human, mouse and chicken genome, and thus are evolutionarily conserved neighbours. Both UCE and CNE sets were mapped to the genome using lastz [53](v1.02.00, parameters '*--gappedthresh=3000 --hspthresh=2500 --seed=match6 --format=general*'). We further filtered these mappings for ≥60% alignment identity and ≥80% alignment coverage. The UCE/CNE sequences are provided as fasta files at https://bds.mpi-cbg.de/hillerlab/TeguGenomeData/ as a resource for further vertebrate assembly completeness assessments.

Repeat annotation

We used RepeatModeler [54](v1.0.8, parameters '*-engine ncbi*') to *de novo* identify repeat families in the tegu genome. Then, we used RepeatMasker (v4.0.5, default parameters) with the resulting repeat library to soft-mask the tegu genome, and ran Tandem Repeat Finder [55]to annotate simple and tandem repeats. We applied the same procedure to the genomes of all other analyzed squamates.

Gene annotation

In order to annotate genes in the tegu genome, we prepared the following three evidence-based datasets: First, we used our assembled PASA transcripts, which were passed to MAKER via the est_gff option in the maker_opts.ctl file. Second, we downloaded reptile protein sequences available on UNIPROT (data accessed in March/April.2018), and mapped the 3,739 proteins for which there is strong experimental evidence (sequences annotated with PE=1 or PE=2) to the tegu v2 genome with exonerate [36](v2.2.20, parameters '*-m protein2genome --subopt 0 -M 20000 -D 2000 --minintron 20 --maxintron 50000 --softmasktarget T --proteinhspdropoff 20 --exhaustive no --refine region --bestn 1*'). This resulted in 3,637 mappings for 3,607 proteins, which were passed to MAKER via the

protein_gff option in the maker_opts.ctl file. Third, we mapped human genes to the tegu lizard genome with CESAR [37]. We selected 20,145 transcripts corresponding to the longest isoform of human Ensembl genes downloaded from UCSC genome browser (hg38 ensGene table), and, based on our pairwise whole genome alignment (below), annotated exons with an intact reading frame and consensus splice sites in the tegu lizard genome. We filtered out mappings corresponding to single-exon genes that were smaller than 100 bp, and mappings spanning more than 10 Mb. This resulted in 14,636 genes which were passed to MAKER via the pred_gff option in the maker_opts.ctl file. In addition to evidence-based datasets, we also used *de novo* gene prediction using Augustus [56] with a previously-obtained gene model [22] and specified the MAKER augustus_species option in the maker_opts.ctl file.

We ran MAKER [35](v2.31.9), setting *est2genome* and *protein2genome=1*, *max_dna_len=300000*, *min_contig=100*, *always_complete=1*, *keep_preds=0*, *split_hit=10000*, *single_exon=1*, *single_length=150*, and *correct_est_fusion=1*.

Multiple genome alignment

We first computed pairwise genome alignments between tegu and other reptiles and amniotes using the lastz/chain/net pipeline, as described in [37, 52]. To this end, we used lastz [53] (v1.04.00) with alignment parameters '*K=2200 L=3000 Y=9400 H=2000*' and the default scoring matrix for aligning reptile species to the tegu genome. The same parameters were used to align non-squamate species, except that we set *Y=3400* and used the HoxD55 scoring matrix. We next built co-linear alignment chains with axtChain [57] using default parameters and applied chainCleaner [58] (parameters *-LRfoldThreshold=2.5 -doPairs -LRfoldThresholdPairs=10 -maxPairDistance=10000 -maxSuspectScore=100000 -minBrokenChainScore=75000*) to improve alignment specificity. For non-squamate species, which are separated from the tegu by >0.72 neutral substitutions per site, we subsequently ran an additional round of highly sensitive local alignments with lastz to uncover additional alignments that were missed before. To this end, we used the parameters '*K=1500 L=2500*

W=5' on all non-aligning regions flanked by local alignments in the chains that are between 20 bp and 100 Kb long. As shown in [37, 52], this procedure is able to uncover numerous additional alignments to exons and CNEs. All local alignments were quality-filtered by requiring that each alignment contains at least one ≥30 bp region with ≥60% sequence identity and ≥1.8 bits entropy as described in [52]. We then generated alignment nets from the chains using chainNet [57] with default parameters. We removed low-scoring alignment nets that are unlikely to represent real homologies by running a non-nested filtering procedure that keeps only nets that span ≥4 Kb in both genomes and have a score ≥20,000. Nets that represent inversions or local translocations and have a score ≥10,000 were also kept. Finally, we used Multiz [59] to produce a multiple alignment from all filtered pairwise alignment nets. The phylogenetic position of the squamate species was taken from [60]. We estimated neutral branch lengths in the phylogenetic tree using phyloFit [38] with parameters '*--EM --precision HIGH --subst-mod REV*' and 4-fold degenerated third codon positions based on our gene annotation.

Annotating conserved regions

To detect genomic regions that are under evolutionary constraint, we applied PhastCons [38] (parameters '*expected-length=45, target-coverage=0.3 rho=0.3*') and GERP [39] (default parameters) to our multiple alignment using the phylogenetic tree with neutral branch lengths. We merged both PhastCons and GERP sets of conserved regions, joined those regions separated by ≤10 bp and filtered the resulting ones for a minimum size of 30 bp. Finally, we only kept conserved regions that align well to at least 4 of the 9 non-tegu squamates in the tegu-based alignment.

To obtain conserved non-exonic elements (CNEs), we excluded all bases from the full set of conserved elements that overlap exons in our CESAR or MAKER gene annotation. Specifically, we subtracted exonic bases from all bases in conserved elements and required that the resulting CNEs are at least 30 bp long.

We defined two subsets of CNEs, a reptile-specific set and a non-reptile-specific one, based on well-aligning regions in other species. For each species in the multiple alignment, we determined all windows of ≥30 bp where the alignment identity is ≥60%. To define the reptile-specific subset, we selected those CNEs that overlap these aligning windows in at least 6 of the 9 reptiles and not a single non-reptile amniote. To define the non-reptile-specific subset, we selected those CNEs that overlap aligning windows in at least 6 of the 9 reptiles and overlap aligning windows in at least one non-reptile amniote. To determine the overlap between CNEs and transposons, we considered SINE, LINE, LTR and DNA transposons from our RepeatMasker annotation and extracted CNEs that overlap transposons for at least 30 bp.

Data availability

All data, including the tegu lizard genome assembly, its gene annotation, the multiple genome alignment and conserved element datasets are available at https://bds.mpi-cbg.de/hillerlab/TeguGenomeData/.

Acknowledgments

We would like to thank the Computer Service Facilities of the MPI-CBG and MPI-PKS, and the Scientific Computing Facility of the MPI-CBG for their support, and Thomas Hackl and Peter Steinbach for help with error correction. This work was supported by the Max Planck Society, by FAPESP stipends 2012/01319-8 and 2012/23360 to JGR, and by the Tschira foundation.

References

1. Alfoldi J, Di Palma F, Grabherr M, Williams C, Kong L, Mauceli E, et al. The genome of the green anole lizard and a comparative analysis with birds and mammals. Nature. 2011;477 7366:587-91. doi:10.1038/nature10390.
2. Xiong Z, Li F, Li Q, Zhou L, Gamble T, Zheng J, et al. Draft genome of the leopard gecko, Eublepharis macularius. Gigascience. 2016;5 1:47. doi:10.1186/s13742-016-0151-4.
3. Liu Y, Zhou Q, Wang Y, Luo L, Yang J, Yang L, et al. Gekko japonicus genome reveals evolution of adhesive toe pads and tail regeneration. Nat Commun. 2015;6:10033. doi:10.1038/ncomms10033.
4. Song B, Cheng S, Sun Y, Zhong X, Jin J, Guan R, et al. A genome draft of the legless anguid lizard, Ophisaurus gracilis. Gigascience. 2015;4:17. doi:10.1186/s13742-015-0056-7.
5. Georges A, Li Q, Lian J, O'Meally D, Deakin J, Wang Z, et al. High-coverage sequencing and annotated assembly of the genome of the Australian dragon lizard Pogona vitticeps. Gigascience. 2015;4:45. doi:10.1186/s13742-015-0085-2.
6. Gao J, Li Q, Wang Z, Zhou Y, Martelli P, Li F, et al. Sequencing, de novo assembling, and annotating the genome of the endangered Chinese crocodile lizard Shinisaurus crocodilurus. Gigascience. 2017;6 7:1-6. doi:10.1093/gigascience/gix041.
7. Bradnam KR, Fass JN, Alexandrov A, Baranay P, Bechner M, Birol I, et al. Assemblathon 2: evaluating de novo methods of genome assembly in three vertebrate species. Gigascience. 2013;2 1:10. doi:10.1186/2047-217X-2-10.
8. Castoe TA, de Koning AP, Hall KT, Card DC, Schield DR, Fujita MK, et al. The Burmese python genome reveals the molecular basis for extreme adaptation in snakes. Proc Natl Acad Sci U S A. 2013;110 51:20645-50. doi:10.1073/pnas.1314475110.
9. Crotalus horridus (timber rattlesnake). https://www.ncbi.nlm.nih.gov/assembly/727941.
10. Gilbert C, Meik JM, Dashevsky D, Card DC, Castoe TA and Schaack S. Endogenous hepadnaviruses, bornaviruses and circoviruses in snakes. Proc Biol Sci. 2014;281 1791:20141122. doi:10.1098/rspb.2014.1122.
11. Vonk FJ, Casewell NR, Henkel CV, Heimberg AM, Jansen HJ, McCleary RJ, et al. The king cobra genome reveals dynamic gene evolution and adaptation in the snake venom system. Proc Natl Acad Sci U S A. 2013;110 51:20651-6. doi:10.1073/pnas.1314702110.
12. Castoe TA, Bronikowski AM, Brodie ED, 3rd, Edwards SV, Pfrender ME, Shapiro MD, et al. A proposal to sequence the genome of a garter snake (Thamnophis sirtalis). Stand Genomic Sci. 2011;4 2:257-70. doi:10.4056/sigs.1664145.
13. Ullate-Agote A, Milinkovitch MC and Tzika AC. The genome sequence of the corn snake (Pantherophis guttatus), a valuable resource for EvoDevo studies in squamates. Int J Dev Biol. 2014;58 10-12:881-8. doi:10.1387/ijdb.150060at.
14. Aird SD, Arora J, Barua A, Qiu L, Terada K and Mikheyev AS. Population Genomic Analysis of a Pitviper Reveals Microevolutionary Forces Underlying Venom Chemistry. Genome Biol Evol. 2017;9 10:2640-9. doi:10.1093/gbe/evx199.
15. Vipera berus berus (common viper). https://www.ncbi.nlm.nih.gov/assembly/233891.
16. Uetz P: The reptile database. http://www.reptile-database.org/. Accessed march.2018.
17. Ávila-Pires TC. Lizards of the brazilian Amazon (Reptilia:Squamata). Zool Verhandelingen (Leiden). 1995;299:706.
18. Presch W. A review of the tegu lizards genus Tupinambis (Sauria: Teiidae) from south America. Copeia. 1973;4:6.
19. Péres J. *Sistemática e conservação de lagartos do gênero Tupinambis (Squamata, Teiidae)*. Universidade de Brasília, 2003.

20. Mazzotti FJMMRMRRNEJKVJEJW, J. Tupinambis merianae as nest predators of crocodilians and turtles in Florida, USA. . Biological Invasions. 2015;17:3.
21. Gnerre S, Maccallum I, Przybylski D, Ribeiro FJ, Burton JN, Walker BJ, et al. High-quality draft assemblies of mammalian genomes from massively parallel sequence data. Proc Natl Acad Sci U S A. 2011;108 4:1513-8. doi:10.1073/pnas.1017351108.
22. Roscito JGS, K.; Parra, G.; Langer, B.; Petzold, A.; Rodrigues, M.T; Hiller, H. Phenotype loss is associated with widespread divergence of the gene regulatory landscape in evolution. https://www.biorxiv.org/content/early/2017/12/22/238634 BioRxiv. 2017 .
23. Hackl T, Hedrich R, Schultz J and Forster F. proovread: large-scale high-accuracy PacBio correction through iterative short read consensus. Bioinformatics. 2014;30 21:3004-11. doi:10.1093/bioinformatics/btu392.
24. Kosugi S, Hirakawa H and Tabata S. GMcloser: closing gaps in assemblies accurately with a likelihood-based selection of contig or long-read alignments. Bioinformatics. 2015;31 23:3733-41. doi:10.1093/bioinformatics/btv465.
25. Boetzer M, Henkel CV, Jansen HJ, Butler D and Pirovano W. Scaffolding pre-assembled contigs using SSPACE. Bioinformatics. 2011;27 4:578-9. doi:10.1093/bioinformatics/btq683.
26. Nowoshilow S, Schloissnig S, Fei JF, Dahl A, Pang AWC, Pippel M, et al. The axolotl genome and the evolution of key tissue formation regulators. Nature. 2018;554 7690:50-5. doi:10.1038/nature25458.
27. Grohme MA, Schloissnig S, Rozanski A, Pippel M, Young GR, Winkler S, et al. The genome of Schmidtea mediterranea and the evolution of core cellular mechanisms. Nature. 2018;554 7690:56-61. doi:10.1038/nature25473.
28. Pacbio github page. https://github.com/PacificBiosciences/Bioinformatics-Training/wiki/Large-Genome-Assembly-with-PacBio-Long-Reads.
29. Berlin K, Koren S, Chin CS, Drake JP, Landolin JM and Phillippy AM. Assembling large genomes with single-molecule sequencing and locality-sensitive hashing. Nat Biotechnol. 2015;33 6:623-30. doi:10.1038/nbt.3238.
30. Chakraborty M, Baldwin-Brown JG, Long AD and Emerson JJ. Contiguous and accurate de novo assembly of metazoan genomes with modest long read coverage. Nucleic Acids Res. 2016;44 19:e147. doi:10.1093/nar/gkw654.
31. Simao FA, Waterhouse RM, Ioannidis P, Kriventseva EV and Zdobnov EM. BUSCO: assessing genome assembly and annotation completeness with single-copy orthologs. Bioinformatics. 2015;31 19:3210-2. doi:10.1093/bioinformatics/btv351.
32. Bejerano G, Pheasant M, Makunin I, Stephen S, Kent WJ, Mattick JS, et al. Ultraconserved elements in the human genome. Science. 2004;304 5675:1321-5. doi:10.1126/science.1098119.
33. Woolfe A, Goodson M, Goode DK, Snell P, McEwen GK, Vavouri T, et al. Highly conserved non-coding sequences are associated with vertebrate development. PLoS Biol. 2005;3 1:e7. doi:10.1371/journal.pbio.0030007.
34. Visel A, Prabhakar S, Akiyama JA, Shoukry M, Lewis KD, Holt A, et al. Ultraconservation identifies a small subset of extremely constrained developmental enhancers. Nat Genet. 2008;40 2:158-60. doi:10.1038/ng.2007.55.
35. Cantarel BL, Korf I, Robb SM, Parra G, Ross E, Moore B, et al. MAKER: an easy-to-use annotation pipeline designed for emerging model organism genomes. Genome Res. 2008;18 1:188-96. doi:10.1101/gr.6743907.
36. Slater GS and Birney E. Automated generation of heuristics for biological sequence comparison. BMC Bioinformatics. 2005;6:31. doi:10.1186/1471-2105-6-31.
37. Sharma V, Schwede P and Hiller M. CESAR 2.0 substantially improves speed and accuracy of comparative gene annotation. Bioinformatics. 2017;33 24:3985-7. doi:10.1093/bioinformatics/btx527.
38. Siepel A, Bejerano G, Pedersen JS, Hinrichs AS, Hou M, Rosenbloom K, et al. Evolutionarily conserved elements in vertebrate, insect, worm, and yeast genomes. Genome Res. 2005;15 8:1034-50. doi:10.1101/gr.3715005.

39. Davydov EV, Goode DL, Sirota M, Cooper GM, Sidow A and Batzoglou S. Identifying a high fraction of the human genome to be under selective constraint using GERP++. PLoS Comput Biol. 2010;6 12:e1001025. doi:10.1371/journal.pcbi.1001025.
40. Simonti CN, Pavlicev M and Capra JA. Transposable Element Exaptation into Regulatory Regions Is Rare, Influenced by Evolutionary Age, and Subject to Pleiotropic Constraints. Mol Biol Evol. 2017;34 11:2856-69. doi:10.1093/molbev/msx219.
41. Martin M. Cutadapt removes adapter sequences from high-throughput sequencing reads. EMBnetjournal Bioinformatics in Action 2011;17.
42. Sameith K, Roscito JG and Hiller M. Iterative error correction of long sequencing reads maximizes accuracy and improves contig assembly. Brief Bioinform. 2015;18 1:1-8. doi:10.1093/bib/bbw003.
43. Luo R, Liu B, Xie Y, Li Z, Huang W, Yuan J, et al. SOAPdenovo2: an empirically improved memory-efficient short-read de novo assembler. Gigascience. 2012;1 1:18. doi:10.1186/2047-217X-1-18.
44. Quiver. https://github.com/PacificBiosciences/GenomicConsensus.
45. Bionano genomics. www.bionanogenomics.com.
46. Freebayes. https://github.com/ekg/freebayes.
47. Bcftools. https://samtools.github.io/bcftools/bcftools.html.
48. Kim D, Langmead B and Salzberg SL. HISAT: a fast spliced aligner with low memory requirements. Nat Methods. 2015;12 4:357-60. doi:10.1038/nmeth.3317.
49. Trapnell C, Williams BA, Pertea G, Mortazavi A, Kwan G, van Baren MJ, et al. Transcript assembly and quantification by RNA-Seq reveals unannotated transcripts and isoform switching during cell differentiation. Nat Biotechnol. 2010;28 5:511-5. doi:10.1038/nbt.1621.
50. Grabherr MG, Haas BJ, Yassour M, Levin JZ, Thompson DA, Amit I, et al. Full-length transcriptome assembly from RNA-Seq data without a reference genome. Nat Biotechnol. 2011;29 7:644-52. doi:10.1038/nbt.1883.
51. Haas BJ, Delcher AL, Mount SM, Wortman JR, Smith RK, Jr., Hannick LI, et al. Improving the Arabidopsis genome annotation using maximal transcript alignment assemblies. Nucleic Acids Res. 2003;31 19:5654-66.
52. Hiller M, Agarwal S, Notwell JH, Parikh R, Guturu H, Wenger AM, et al. Computational methods to detect conserved non-genic elements in phylogenetically isolated genomes: application to zebrafish. Nucleic Acids Res. 2013;41 15:e151. doi:10.1093/nar/gkt557.
53. Harris RS. *Improved pairwise alignment of genomic DNA.* The Pennsylvania State University, , 2007.
54. Repeat masker. http://www.repeatmasker.org/.
55. Tandem repeat finder. https://tandem.bu.edu/trf/trf.html.
56. Stanke M and Waack S. Gene prediction with a hidden Markov model and a new intron submodel. Bioinformatics. 2003;19 Suppl 2:ii215-25.
57. Kent WJ, Baertsch R, Hinrichs A, Miller W and Haussler D. Evolution's cauldron: duplication, deletion, and rearrangement in the mouse and human genomes. Proc Natl Acad Sci U S A. 2003;100 20:11484-9. doi:10.1073/pnas.1932072100.
58. Suarez HG, Langer BE, Ladde P and Hiller M. chainCleaner improves genome alignment specificity and sensitivity. Bioinformatics. 2017;33 11:1596-603. doi:10.1093/bioinformatics/btx024.
59. Blanchette M, Kent WJ, Riemer C, Elnitski L, Smit AF, Roskin KM, et al. Aligning multiple genomic sequences with the threaded blockset aligner. Genome Res. 2004;14 4:708-15. doi:10.1101/gr.1933104.
60. Pyron RA, Burbrink FT and Wiens JJ. A phylogeny and revised classification of Squamata, including 4161 species of lizards and snakes. BMC Evol Biol. 2013;13:93. doi:10.1186/1471-2148-13-93.

Figures

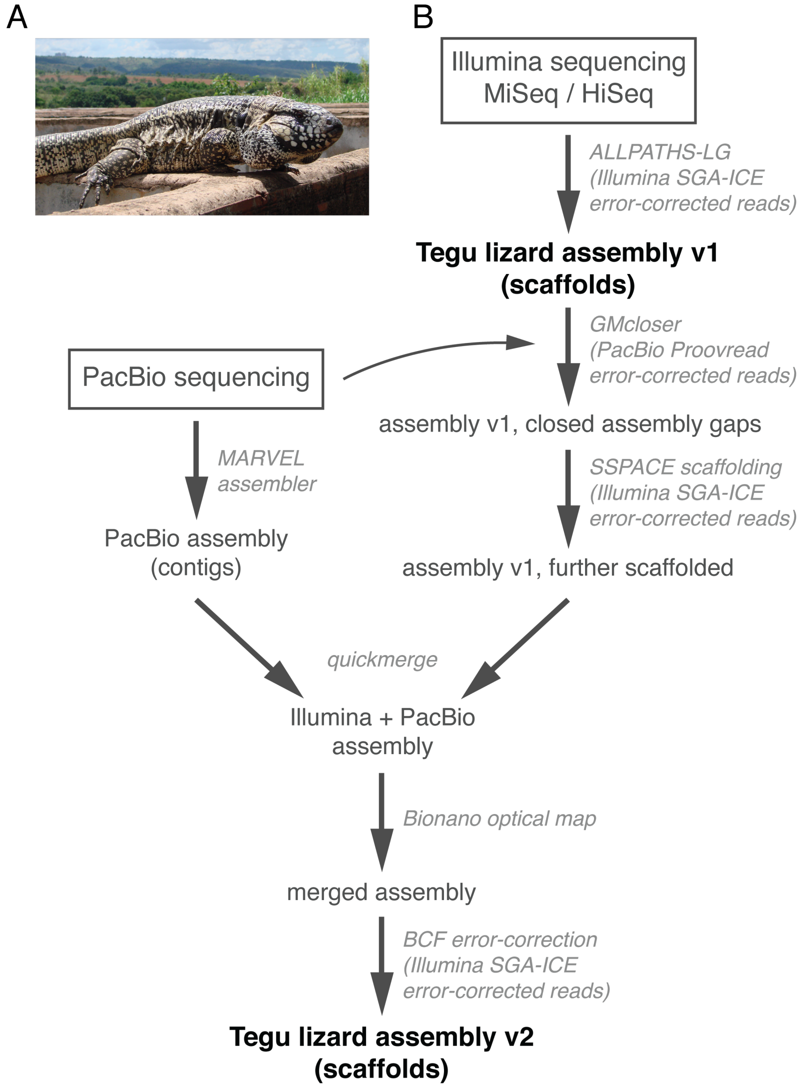

Figure 1: Workflow to generate the tegu lizard v2 assembly.

(A) The tegu lizard *Salvator merianae.*

(B) Assembly v1 was built entirely from Illumina short read data. To improve this assembly, we used PacBio long read data to close assembly gaps and extend scaffolds, and merged the improved Illumina with a PacBio-only assembly. Finally, optical mapping data was used to resolve contig chimeras and scaffold even further. Used tools and their input data are shown in grey.

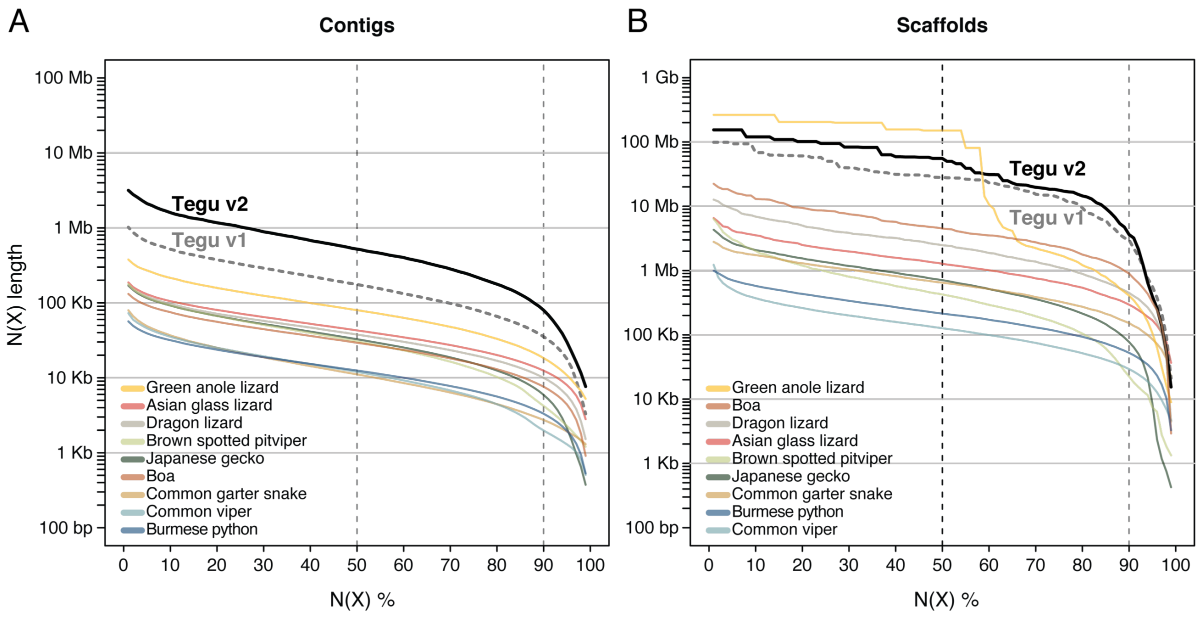

Figure 2: Comparison of assembly contiguity.

N(x)% graphs show the contig (A) and scaffold (B) sizes (y-axis), where x% of the genome assembly consists of contigs and scaffolds of at least that size. The tegu lizard v1 and v2 assemblies are shown in grey and black. All other assemblies are sorted by the N50 values in the insets. Dashed lines mark the N50 and N90 values.

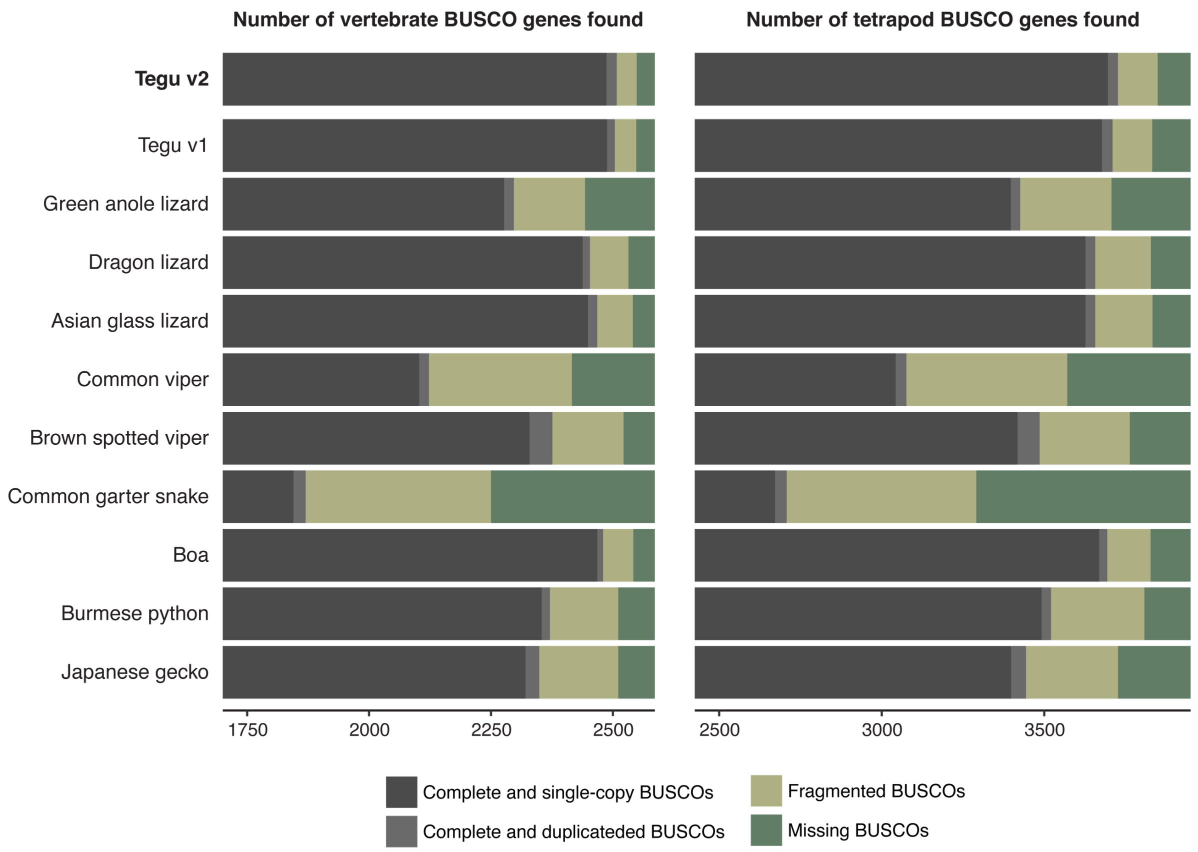

Figure 3: Comparison of genome completeness for coding genes.

The bar charts show the number of complete, fragmented and missing genes using two BUSCO datasets for vertebrate-conserved (left) and tetrapod-conserved (right) genes.

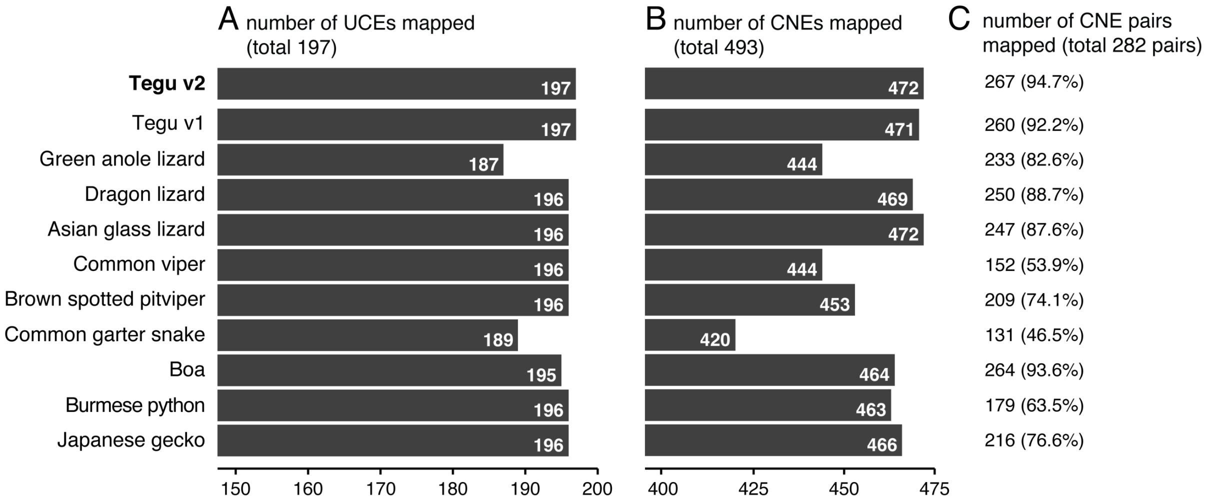

Figure 4: Using conserved non-exonic elements to compare genome completeness and contiguity.

Bar charts show (A) the number of aligning Ultra-Conserved Elements that do not overlap coding regions (UCEs, N=197 in total) and (B) the number of aligning conserved elements that do not overlap exons (CNEs, N=493 in total). (C) The percentage of 282 evolutionarily-conserved pairs of neighbouring CNEs that are also found as neighbours in the reptile assemblies. Both UCE and CNE sets are highly conserved among vertebrates and thus are likely to exist in reptiles.

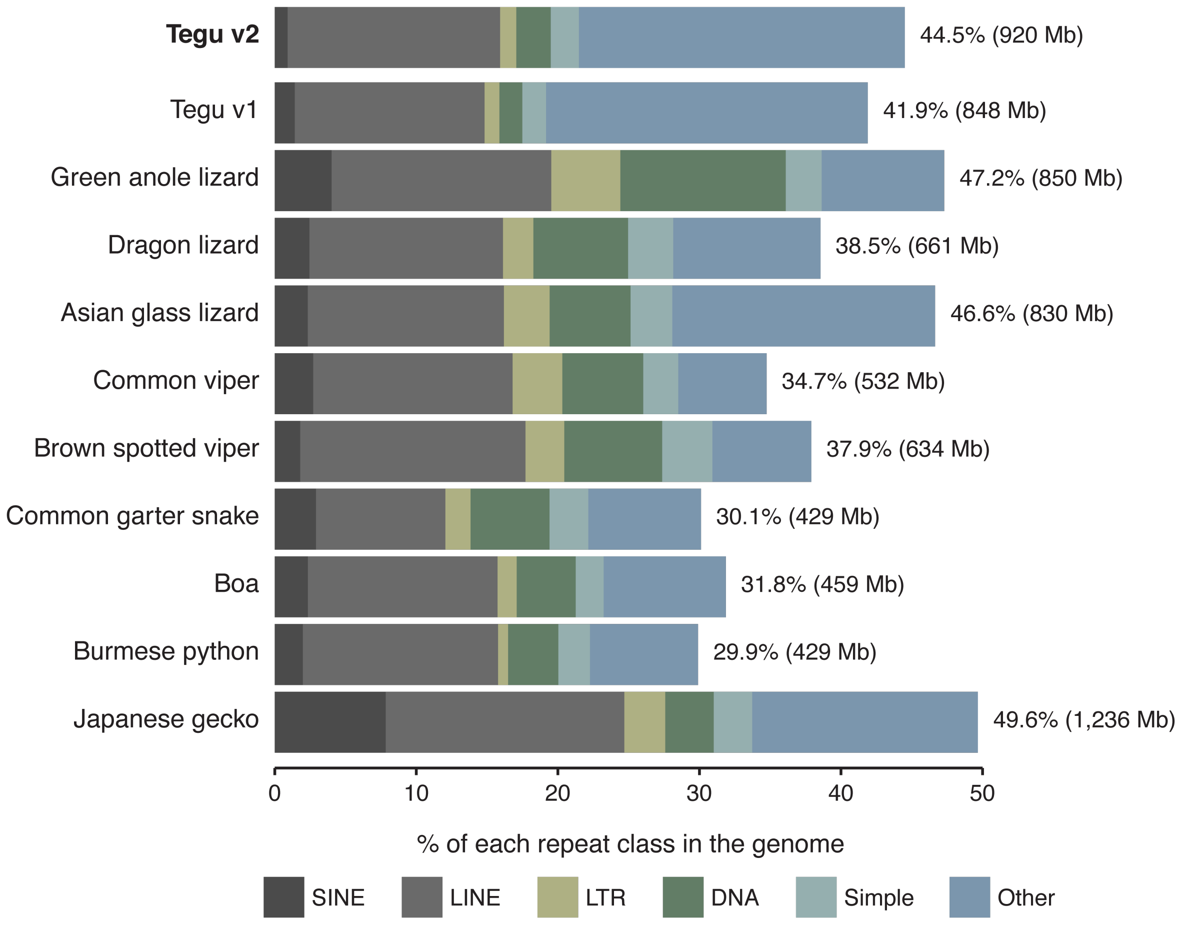

Figure 5: Repeat landscape in reptile genomes.

Major classes of repeats are color-coded and shown as bar charts that represent the portion of the genome they cover. Simple repeats comprise tandem repeats, low complexity regions and satellite repeats.

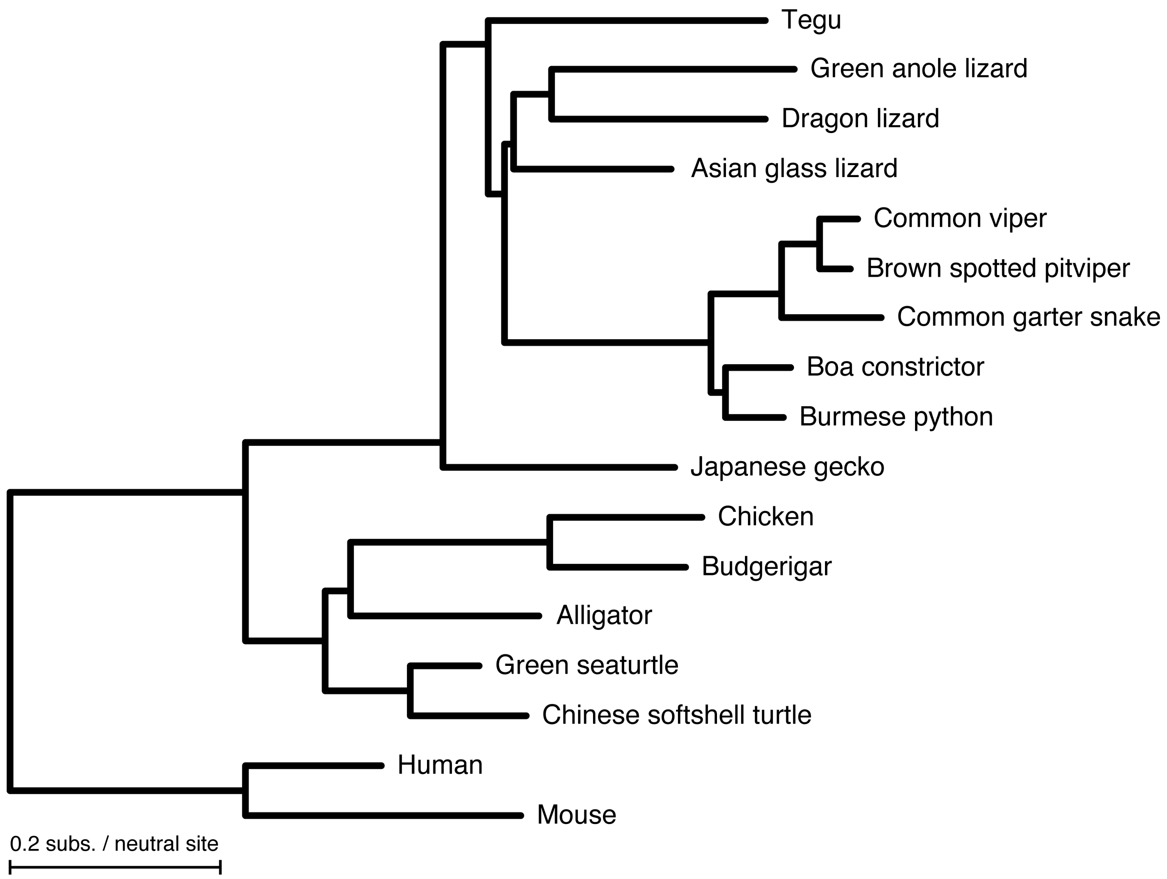

Figure 6: Phylogenetic tree of the amniote species included in our multiple genome alignment.

Branch lengths represent the number of substitutions per neutral site, as estimated from four-fold degenerated codon positions.

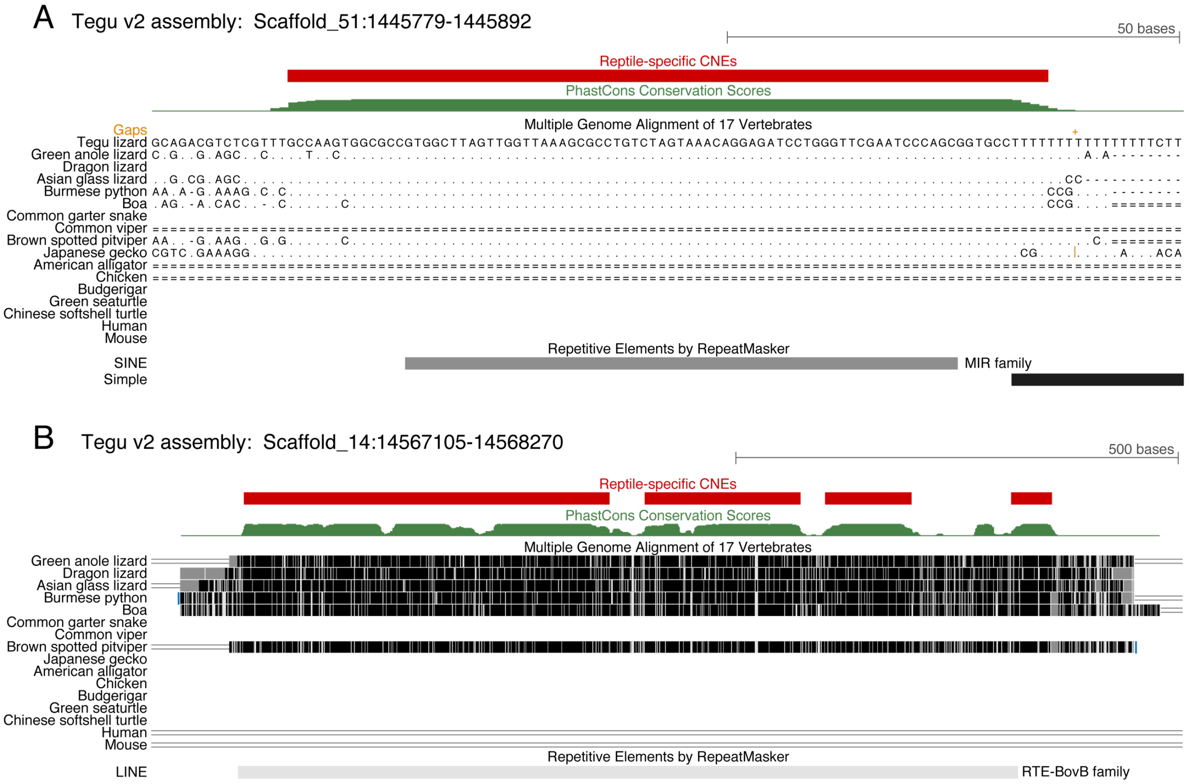

Figure 7: Transposon-derived conserved non-exonic (CNE) reptile-specific elements. (A) A reptile-specific CNE originated from the insertion of a Short Interspersed Nuclear Element (SINE) belonging to the MIR family. The multiple genome alignment shows that this CNE is highly-conserved among reptiles, but does not align to non-reptile species.

(B) Several reptile-specific CNEs originated from the insertion of a LINE of the RTE-BovB family. This insertion likely happened after the split from the lineage leading to geckos as no sequence aligns to the gecko genome.

Supplementary Tables

Click here to access/download
Supplementary Material
SupplementaryTables.xlsx
