# Supplementary material for: The genome of the tegu lizard Salvator merianae: combining Illumina, PacBio, and optical mapping data to generate a highly contiguous assembly
Source: Gigascience. 2018 Nov 27;7(12):giy141. doi: 10.1093/gigascience/giy141 (PMC6304105; doi:10.1093/gigascience/giy141)
Supplement: giga-d-18-00186_revision_1.pdf [file giy141_giga-d-18-00186_revision_1.pdf]

GigaScience

The genome of the tegu lizard Salvator merianae: combining Illumina, PacBio, and optical mapping data to generate a highly contiguous assembly

--Manuscript Draft--

**Manuscript Number:**	GIGA-D-18-00186R1
**Full Title:**	The genome of the tegu lizard Salvator merianae: combining Illumina, PacBio, and optical mapping data to generate a highly contiguous assembly
**Article Type:**	Data Note
**Funding Information:**	Max-Planck-Gesellschaft — Dr. Michael Hiller<br>FAPESP (2012/013198, 2012/23360) — Dr. Juliana Roscito<br>Klaus Tschira Stiftung — Prof. Gene Myers
**Abstract:**	Background: Reptiles are a species-rich group with great phenotypic and life history diversity, but are highly underrepresented among the vertebrate species with sequenced genomes.<br>Results: Here, we report a high-quality genome assembly of the tegu lizard Salvator merianae, the first lacertid with a sequenced genome. We combined 74X Illumina short read, 29.8X PacBio long read and optical mapping data to generate a high-quality assembly with a scaffold N50 value of 55.4 Mb. The contig N50 value of this assembly is 521 Kb, making it the most contiguous reptile assembly so far. We show that the tegu assembly has the highest completeness of coding genes and conserved non-exonic elements (CNEs) compared to other reptiles. Furthermore, the tegu assembly has the highest number of evolutionarily conserved CNE pairs, corroborating a high assembly contiguity in intergenic regions. Like in other reptiles, Long Interspersed Nuclear Elements (LINEs) comprise the most abundant transposon class. We used transcriptomic data, homology- and de novo gene predictions to annotate 22,413 coding genes, of which 16,995 (76%) likely have human orthologs as inferred by CESAR-derived gene mappings. Finally, we generated a multiple genome alignment comprising 10 squamates and 7 other amniote species and identified conserved regions that are under evolutionary constraint. Conserved non-exonic elements cover 38 Mb (1.8%) of the tegu genome, with 3.3 Mb in these elements being squamate-specific. In contrast to placental mammal-specific CNEs, very few of these squamate-specific CNEs (<20 Kb) overlap transposons, highlighting a difference in how lineage-specific CNEs originated in these two clades.<br>Conclusions: The tegu lizard genome together with the multiple genome alignment and comprehensive conserved element datasets provide a valuable resource for comparative genomic studies of reptiles and other amniotes.
**Corresponding Author:**	Michael Hiller<br><br>GERMANY
**Corresponding Author Secondary Information:**	
**Corresponding Author's Institution:**	
**Corresponding Author's Secondary Institution:**	
**First Author:**	Juliana Roscito
**First Author Secondary Information:**	
**Order of Authors:**	Juliana Roscito<br>Katrin Sameith<br>Martin Pippel<br>Kees-Jan Francoijs

Powered by Editorial Manager® and ProduXion Manager® from Aries Systems Corporation

	Sylke Winkler
	Andreas Dahl
	Georg Papoutsoglou
	Gene Myers
	Michael Hiller
**Order of Authors Secondary Information:**	
**Response to Reviewers:**	I uploaded a cover letter and the point-by-point response as two separate Word files under the category 'personal cover' since 'Cover letter' and 'Response to reviewers' was not available as a category
**Additional Information:**	
**Question**	**Response**
Are you submitting this manuscript to a special series or article collection?	No
**Experimental design and statistics**<br><br>Full details of the experimental design and statistical methods used should be given in the Methods section, as detailed in our Minimum Standards Reporting Checklist. Information essential to interpreting the data presented should be made available in the figure legends.<br><br>Have you included all the information requested in your manuscript?	Yes
**Resources**<br><br>A description of all resources used, including antibodies, cell lines, animals and software tools, with enough information to allow them to be uniquely identified, should be included in the Methods section. Authors are strongly encouraged to cite Research Resource Identifiers (RRIDs) for antibodies, model organisms and tools, where possible.<br><br>Have you included the information requested as detailed in our Minimum Standards Reporting Checklist?	Yes
**Availability of data and materials**	Yes

All datasets and code on which the conclusions of the paper rely must be either included in your submission or deposited in publicly available repositories (where available and ethically appropriate), referencing such data using a unique identifier in the references and in the "Availability of Data and Materials" section of your manuscript.<br><br>Have you have met the above requirement as detailed in our Minimum Standards Reporting Checklist?	

Powered by Editorial Manager® and ProduXion Manager® from Aries Systems Corporation

Manuscript Click here to access/download;Manuscript;Manuscript(1).docx
Click here to view linked References

The genome of the tegu lizard *Salvator merianae*: combining Illumina, PacBio, and optical mapping data to generate a highly contiguous assembly

Juliana G. Roscito[1,2,3], Katrin Sameith[1,2,3], Martin Pippel[1,3], Kees-Jan Francoijs[4], Sylke Winkler[1], Andreas Dahl[5], Georg Papoutsoglou[4], Gene Myers[1,3] and Michael Hiller[1,2,3*]

[1] Max Planck Institute of Molecular Cell Biology and Genetics, Dresden, Germany

[2] Max Planck Institute for the Physics of Complex Systems, Dresden, Germany

[3] Center for Systems Biology Dresden, Germany

[4] BioNano Genomics, San Diego, USA

[5] Center for Molecular and Cellular Bioengineering, Technische Universität Dresden, Germany

* To whom correspondence should be addressed:

Michael Hiller

Computational Biology and Evolutionary Genomics, Max Planck Institute of Molecular Cell Biology and Genetics & Max Planck Institute for the Physics of Complex Systems, Dresden, Germany.

Tel: +49 351 210 2781

Fax: +49 351 210 1209

Email: hiller@mpi-cbg.de

Abstract

Background: Reptiles are a species-rich group with great phenotypic and life history diversity, but are highly underrepresented among the vertebrate species with sequenced genomes.

Results: Here, we report a high-quality genome assembly of the tegu lizard *Salvator merianae*, the first lacertid with a sequenced genome. We combined 74X Illumina short read, 29.8X PacBio long read and optical mapping data to generate a high-quality assembly with a scaffold N50 value of 55.4 Mb. The contig N50 value of this assembly is 521 Kb, making it the most contiguous reptile assembly so far. We show that the tegu assembly has the highest completeness of coding genes and conserved non-exonic elements (CNEs) compared to other reptiles. Furthermore, the tegu assembly has the highest number of evolutionarily conserved CNE pairs, corroborating a high assembly contiguity in intergenic regions. Like in other reptiles, Long Interspersed Nuclear Elements (LINEs) comprise the most abundant transposon class. We used transcriptomic data, homology- and *de novo* gene predictions to annotate 22,413 coding genes, of which 16,995 (76%) likely have human orthologs as inferred by CESAR-derived gene mappings. Finally, we generated a multiple genome alignment comprising 10 squamates and 7 other amniote species and identified conserved regions that are under evolutionary constraint. Conserved non-exonic elements cover 38 Mb (1.8%) of the tegu genome, with 3.3 Mb in these elements being squamate-specific. In contrast to placental mammal-specific CNEs, very few of these squamate-specific CNEs (<20 Kb) overlap transposons, highlighting a difference in how lineage-specific CNEs originated in these two clades.

Conclusions: The tegu lizard genome together with the multiple genome alignment and comprehensive conserved element datasets provide a valuable resource for comparative genomic studies of reptiles and other amniotes.

Introduction

Comparative whole-genome analyses are of great importance to understanding the evolutionary trajectory of different species. The increasing number of sequenced genomes from diverse animal groups deepens the power of such comparative analysis, resulting in novel insights into the origin and evolution of many of the shared and unique genomic features that characterize different species.

Squamate reptiles comprise a species-rich group of approximately 6,500 lizards, 3,700 snakes, and 200 amphisbaenian species [1]. However, this group is heavily under-represented among the vertebrate species with sequenced genomes, especially considering the great morphological, behavioral, and life history diversity in this group. The green anole *Anolis carolinensis* was the first lizard to have the genome sequenced [2]. Since then, squamates have been gaining attention for their relevance in understanding vertebrate evolution, as well as for reptile-specific features that are of human interest, such as venom with medical implications and adhesive features of gecko feet. This interest resulted in the sequencing and assembly of additional squamate genomes. Right now, nine snake species (*Boa constrictor*, Burmese python, two rattlesnakes, king cobra, garter snake, corn snake, and two vipers) and six lizards (green anole, two geckos, Asian glass lizard, dragon lizard, Chinese crocodile lizard) have assembled genomes [3-16].

We extend the sampling of lizard species to the tegu lizard *Salvator merianae* (Figure 1A), a teiid lizard and the first representative of the Lacertoidea group with a sequenced genome. Tegus are large, omnivorous reptiles that are generally easy to keep in captivity and have economic importance in South America mainly for leather and meat, in addition to being sold as pets. The tegu lizard, native to South America, is widely distributed in open vegetation areas and also in forested landscapes [1, 17, 18]. Tegus are opportunistic and adapt well to

many environments. Since it preys on crocodile, bird and turtle eggs, tegus frequently become a threat to endangered species [19, 20].

Here, we generated PacBio long-read and Bionano optical mapping data to substantially improve the quality of a previous short read-based tegu genome assembly [21]. We show that the new genome exhibits greatly increased contigs and scaffolds, making it the most contiguous reptile assembly so far. It also has the highest completeness of genes and conserved non-exonic elements compared to the genomes of other reptile species. We further provide repeat and gene annotations for this assembly. Finally, we generated a reptile-based multiple genome alignment comprising 10 squamates and 7 other amniote species and identified reptile-specific conserved genomic regions, altogether providing a valuable resource for comparative reptile genomics.

Results

Overview of the v2 tegu lizard assembly process

The first version of the tegu genome (v1) was assembled with ALLPATHS-LG [22] using high-coverage Illumina sequencing data (41X 2x300 bp MiSeq reads and 33X 2x150 bp HiSeq reads; Supplementary Table 1), resulting in a 2.026 Gb assembly with a scaffold N50 of 28.1 Mb (5,988 scaffolds) [21]. Despite the large scaffolds, the 36,428 contigs have an N50 value of only 175.8 Kb, and 80 Mb (4%) of the assembly consisted of assembly gaps. To upgrade this assembly, we generated 29.8X long sequencing reads with an N50 value of 8.4 Kb using the PacBio platform and corrected base errors in these reads using our Illumina data and the Proovread tool [23] (Supplementary Table 1). Next, we applied GMcloser [24] to close or shrink assembly gaps with these error-corrected PacBio reads. Since GMcloser also extends scaffold ends with the PacBio reads, which could provide new anchor points for Illumina mate-pair reads, we subsequently applied another round of scaffolding using our

Illumina data and SSPACE [25]. Independently of using PacBio data to directly improve the Illumina assembly, we also assembled the PacBio reads into contigs with MARVEL [26, 27]. Because the read coverage of <25X after sequencing artifact correction in MARVEL's patch phase was lower than the minimum recommended 50X coverage to generate a PacBio-only assembly with high completeness [28, 29], we preferred to combine both Illumina and PacBio assemblies into a higher-quality hybrid assembly using quickmerge [30]. To further scaffold and resolve chimeric contigs, we used the Bionano system to generate a *de novo* optical map using molecules longer than 100 Kb. 94.2% of the 'quickmerged' assembly aligned to this optical map, showing that the optical map covered the genome well. We then combined the optical mapping and the quickmerged assembly into a final genome assembly and applied a last round of error-correction using Illumina data. The workflow to generate the final v2 tegu assembly is illustrated in Figure 1B.

The final v2 assembly of the tegu lizard genome has a size of 2.068 Gb, which is close to the 1.904 - 1.905 Gb size estimated by k-mer analysis of Illumina reads. The v2 assembly has scaffold N50/N90 values of 55.4/3.6 Mb and contig N50/N90 values of 521/79.7 Kb (Supplementary Table 2). Compared to the v1 assembly, contig N50/N90 values improved by 3/2.3 fold, and the number of bases in assembly gaps decreased 2.3-fold from 80 to 34.33 Mb.

Comparing contiguity to other reptile assemblies

In comparison to other published squamate reptile genome assemblies, the v2 tegu assembly has the second-largest scaffold N50 value (Figure 2A, Supplementary Table 2). Only the green anole lizard assembly (N50 of 150.6 Mb), which relied on fluorescence in-situ hybridization of bacterial artificial chromosome clones to anchor scaffolds to chromosomes [2], has larger scaffolds. However, the v2 tegu assembly has a scaffold N90 value that is 9-times larger than that of the anole lizard (3.6 Mb vs. 0.41 Mb). Furthermore, the v2 tegu

assembly has the largest contig N50 and N90 values compared to all other assemblies (Figure 2B, Supplementary Table 2), with a 6.5-times larger N50 value than the next best assembly (green anole lizard, 521 vs. 80 Kb). Thus, the v2 tegu assembly represents the most contiguous reptile assembly at the moment.

Comparing assembly completeness

Next, we assessed whether the higher contiguity of the v2 tegu assembly is also reflected in higher completeness in functional genomic regions. First, we used BUSCO [31] to assess genome completeness for genes conserved in vertebrates (vertebrata database; 2,586 genes) and tetrapods (tetrapoda database; 3,950 genes). The v2 tegu genome has BUSCO completeness scores of 97% for the vertebrate gene set and 94.4% for the tetrapod gene set. This represents a slight improvement over the previous v1 assembly and higher scores compared to other reptile genomes (Figure 3, Supplementary Table 3).

Second, we assessed assembly completeness by quantifying the number of highly conserved non-exonic elements that can be found in the tegu genome and in the genomes of other reptiles. We first selected a set of 197 Ultra-Conserved Elements (UCEs, originally defined as genomic regions of longer than 200 bp that are identical between human, mouse and rat [32]; Supplementary Table 4) that are also well-conserved in chicken, zebrafish, and medaka. All 197 UCEs were identified in the tegu lizard genome (both v1 and v2 assemblies), while other reptile assemblies miss at least one of the UCEs (Figure 4A). In addition, we selected a larger set of 493 vertebrate-conserved non-exonic elements (CNEs; Supplementary Table 5), defined as regions longer than 300 bp that are conserved among mammals, teleost fish, shark and lamprey [33], and counted the number of elements that aligned to the genome of each species with at least 80% coverage and 60% identity. We found 472 CNEs (95.7%) in the v2 tegu lizard genome assembly and in the Asian glass lizard genome; all other reptile assemblies (including the tegu v1) contained fewer CNEs (Figure 4B).

In addition, we used our CNE mapping data to assess assembly contiguity in intergenic regions by investigating conservation of CNE synteny. We first defined a set of 282 pairs of neighboring CNEs that are located in the same chromosome and are at most 1 Mb apart from each other in 3 outgroup species (chicken, mouse, and human). Then we asked how many of these pairs could be retrieved in the reptile assemblies. Since CNEs often overlap regulatory elements [34, 35] and maintain a conserved order with respect to their target genes and other CNEs, we expect that both CNEs in such a pair are also identified on the same scaffold in a well-assembled genome. In the v2 tegu assembly, we found 267 (94.7%) of these CNE pairs as pairs located on the same scaffold and also at most 1 Mb apart from each other. The second-best assembly is the boa snake with 264 pairs (Figure 4C). For the green anole lizard only 233 pairs were found, likely because several CNEs align to shorter scaffolds or do not align at all. These results corroborate that the v2 tegu assembly also has a high completeness and contiguity in non-exonic regions, suggesting that this assembly is a valuable resource to study gene regulation in a reptile.

Repeat content

To assess the repeat content of the tegu genome, we modelled and masked repeats in the v2 tegu lizard genome assembly using RepeatModeler and RepeatMasker. A high proportion of the genome (44.5%) was annotated as repeats, with Long Interspersed Nuclear Elements (LINEs) comprising the largest repeat class (Figure 5). The v2 assembly contained 72 additional Mb in repeat-masked sequence that was not present in the v1 assembly. We also modelled and masked repeats in the other reptile genomes analyzed in this study and found a similar repeat content, with the exception of snakes that generally have fewer repeats (29-38% vs. 38-50% for non-snake reptiles; Figure 5, Supplementary Table 6).

Tegu gene annotation

To annotate genes in the tegu lizard genome, we used MAKER [36] with four types of input data: transcriptome data, protein sequences from 33 sauropsid species (Supplementary Table 7), human genes mapped to the tegu lizard genome, and gene predictions based on the gene annotation of the v1 assembly. First, we used RNA-seq data obtained from tegu lizard tissues [21] that we assembled to 304,367 transcripts. Second, we mapped sauropsid protein sequences available on UNIPROT to the tegu genome using exonerate [37], resulting in 3,637 high-quality homology-derived gene models. Third, we mapped human genes to the tegu genome using CESAR [38, 39], which resulted in 16,995 mappings that align at least partially to the tegu genome. Since CESAR was run on a genome alignment that makes extensive use of conserved alignment order, these 16,995 tegu loci likely contain orthologs of human genes. Fourth, we used BRAKER [40] to obtain gene predictions based on mapped RNAseq data and the previous gene set from v1 assembly, resulting in 75,444 predictions after removing short, overlapping genes. The final gene set produced by MAKER contains 22,413 genes (BUSCO completeness score of 94.1%), which is within the range of the number of genes annotated in other lizards [2, 4, 12, 13, 16].

Generating a resource for comparative reptile genomics

To facilitate using the v2 tegu lizard assembly for comparative genomics, we generated a reptile-focused, highly sensitive, multiple genome alignment. Since the tegu lizard genome is currently the most contiguous assembly, we used it as the reference. Our alignment includes 10 squamates and 7 other outgroup species such as mouse, human, birds, turtles, and alligator (Figure 6, Supplementary Table 8). To detect genomic regions that are under evolutionary constraint, we integrated conserved regions detected by PhastCons [41] and GERP [42]. The multiple alignment, the conserved regions, and the tegu gene annotation are available at https://bds.mpi-cbg.de/hillerlab/TeguGenomeData/ and can be loaded into the UCSC genome browser as an assembly hub [43] with the hub URL https://bds.mpi-cbg.de/hillerlab/TeguGenomeData/assemblyHub/hub.txt.

CNE analysis

We intersected the conserved elements with our gene annotation to extract conserved regions that do not overlap exons. This resulted in 324,770 CNEs, covering 38 Mb (1.83% of the tegu genome). We further used our genome alignment to extract a CNE subset that is only conserved among squamates, resulting in 47,931 squamate-specific CNEs (3.3 Mb, 0.16% of the tegu genome). By intersecting squamate-specific CNEs with transposons, we found that only 146 of the 47,931 CNEs (0.3%) overlap transposons (Figure 7). These 146 CNEs sum up to 19.8 Kb, 86% of which overlap LINEs, consistent with this transposon class being the most abundant one in the assembly (Figure 5). Overall, transposons may have given rise to only 0.6% (19.8 kb of 3.3 Mb) of the bases in squamate-specific CNEs.

Discussion

Here we present a high-quality genome assembly for the tegu lizard, the first sequenced Lacertoidea species. We combined Illumina short read with PacBio long read sequencing and Bionano optical mapping technologies to obtain an assembly with fewer gaps. In comparison to other available reptile genomes, the tegu v2 assembly has the longest contigs and thus the highest contiguity, and also a higher completeness of genes and non-exonic elements. Furthermore, the new v2 assembly contains several additional megabases of repetitive sequences that were not present in the previous Illumina-based v1 assembly. This illustrates the ability of long PacBio reads to add repetitive sequence to a short-read assembly and thus increase the completeness in the repeat content of an assembly.

We found that all analyzed reptile genomes have a similar repeat composition, with LINEs making up the largest portion. Interestingly, even though the repeat content of reptile genomes is fairly high, very few of the squamate-specific CNEs overlap transposable elements. This contrasts previous observations in mammals, where 16% of the placental mammal-specific CNEs overlap transposons [44]. Squamates are an older lineage compared

to mammals (~200 vs. ~100 Mya), which makes the identification of ancient squamate-specific repeats more challenging. Nevertheless, the CNE-transposon overlap is more than 50-fold lower in squamates, which highlights a difference in how functional lineage-specific non-exonic elements evolved in these two clades.

We assessed and compared assembly completeness not only considering coding genes, but also considering conserved non-exonic elements that often have regulatory activity. Furthermore, we used evolutionarily conserved pairs of CNEs as a novel measure of assembly contiguity. Together, this allows evaluating assembly completeness and contiguity in the non-exonic regions of an assembly. Since *cis*-regulatory elements typically reside in non-exonic regions, it is important to have a high completeness and contiguity in the intergenic portion of the genome, especially when applying high-throughput functional genomics methods, such as ChIP–seq or ATAC-seq, to discover regulatory elements.

To facilitate using the tegu genome for comparative studies focusing on the evolution of *cis*-regulatory elements, we generated a multiple genome alignment of 9 other reptiles and 7 other amniotes, and annotated a comprehensive set of conserved non-exonic elements. The alignment and CNE sets provide a valuable resource for comparative reptile genomics and will help to understand genome evolution in vertebrates.

Methods

Ethics, consent and permissions

All DNA samples were derived from a liquid nitrogen snap-frozen liver tissue sample from a single male individual of the tegu lizard *Salvator merianae*, collected in the state of Mato Grosso, Brazil (specimen accession number LG2117), in accordance with the Brazilian environmental and scientific legislation, under the SISBIO (Sistema de Autorização e

Informação em Biodiversidade, Instituto Chico Mendes de Conservação da Biodiversidade) license 30309-4.

DNA extraction and library preparation

DNA for Illumina and PacBio libraries was isolated after lysis of liver tissue in QIAgen Q2 lysis buffer with Proteinase K and standard phenol-chloroform extraction. High molecular weight genomic DNA was precipitated by centrifugation after adding ice-cold Ethanol and dissolved in Tris-EDTA, pH 8.0. All pipetting steps were carefully done with wide bore pipetting tips to avoid any damage to the genomic DNA. RNA was removed by RNase A treatment. Pulse field gel electrophoresis (PFGE, SAGE Pippinpulse) showed that the resulting DNA molecules were between 50 and 200 Kb long.

Extraction of megabase genomic DNA for Bionano optical mapping was done according to the IrysPrep™ Animal Tissue protocol (Bionano Tech Note v. 1.1.12). Briefly, cell nuclei were isolated from embryonic tegu tissue and embedded in agarose plugs. After Proteinase K and RNAse treatment of plugs, genomic DNA was extracted from agarose plugs and cleaned by drop dialysis against 1x TE. PFGE revealed DNA molecules with a minimum of 100 Kb and up to 1 Mb of length.

For transcriptome sequencing, we extracted total RNA from two tegu lizard embryos. Tissues were immediately frozen in liquid nitrogen and total RNA was later extracted following a standard Trizol extraction.

Sequencing

Illumina sequencing

Sequencing of the tegu genome with the Illumina platform is described in detail in [21]. Briefly, we sequenced 2x300 bp reads from three libraries on the MiSeq platform to a coverage of 41.3X, and sequenced 2x150 bp reads from two 2 Kb mate-pair libraries and

from two 10 Kb mate-pair libraries on the HiSeq 2500 to a coverage of 32.7X after adapter trimming. To obtain transcriptomic data to annotate genes, we sequenced 2x75 bp reads from eight strand-specific mRNA libraries on the Illumina HiSeq 2500 platform.

PacBio sequencing

Long insert libraries were prepared as recommended by Pacific Biosciences according to the Guidelines for preparing size-selected 20 Kb SMRTbell™ templates. Covaris g-Tubes™ were used for shearing 10 μg genomic DNA following the manufacturer instructions to fragments sizes of 10 to 25 Kb. The PacBio SMRTbell™ library was size selected for fragments larger than 9 Kb making use of the SAGE BluePippin™ devise. A second large insert library was prepared as described, but shearing of genomic DNA to 40 Kb fragments was done with the MegaRuptor™ device (Diagenode) and this PacBio SMRTbell™ library was size selected for fragments larger than 10 Kb. A total of 205 SMRT cells were sequenced on the PacBio RSII instrument making use of P4 polymerase and C2 sequencing chemistry. Movie length was 3 hours for all SMRT cells.

Optical map

We delivered high molecular weight DNA embedded in agarose gel to the VIB Nucleomics Core. The purified DNA sequence-specific labelling was performed by the Nick, Labelling, Repair and Staining steps according to IrysPrep TM NLRS assay (900 ng) version 30024D. Sequence specificity was provided by the nickase Nt.BspQ1 using a concentration between 5U and 7U. Labelling was carried out by a nick translation process in the presence of a fluorophore-labelled nucleotide. The labelled nicks were repaired to restore strand integrity and DNA molecules were stained for visualization of the backbone visualization. The molecules were imaged using the Irys system, loading stained molecules automatically into Bionano Genomics nanochannel chips using electrophoresis. Label positions and lengths of DNA molecules were recorded by the on-board CCD camera using green and blue lasers in the Bionano Genomics Irys system. Data was generated from a total of 5 flow cells.

Genome assembly

Illumina-only assembly

We previously generated an assembly using only Illumina sequencing data [21]. Briefly, we used cutadapt (cutadapt, RRID:SCR_011841)[45] (v1.5) to trim adapters in the raw Illumina sequencing reads, iteratively corrected sequencing errors with the SGA-ICE pipeline [46], and assembled the error-corrected MiSeq and HiSeq reads using ALLPATHS-LG (ALLPATHS-LG , RRID:SCR_010742)[22] (v52188, parameters '*CLOSE_UNIPATH_GAPS=False HAPLOIDIFY=True*'). Details of this previous Illumina-only assembly are described in [21].

Next, we improved this Illumina assembly by closing gaps and further scaffolding using PacBio data generated for this study. We first applied SOAP gapcloser (GapCloser, RRID:SCR_015026)[47] (v1.12, default parameters) with the SGA-ICE error-corrected MiSeq and HiSeq reads as input to resolve ambiguous base positions (N's) that typically represent SNPs. To correct sequencing errors in the PacBio reads, we used our SGA-ICE error-corrected MiSeq reads and Proovread [23] with the bwa mapper, first seeding with 12-mers and subsequently seeding with 13-mers. Then, we used GMcloser (GMcloser, RRID:SCR_000646)[24] (v1.5, parameter '*min_gap_size 200*') with the error-corrected PacBio reads as input and the *--extend* parameter set to fill gaps and extend scaffold ends with aligning PacBio reads. This gap closing step decreased the number of assembly gaps (runs of ≥25 N's) from 28,792 to 11,628. Finally, we further scaffolded the scaffolds with extended ends with SSPACE (SSPACE , RRID:SCR_005056)[25] (v2.0, default parameters) and the SGA-ICE corrected Illumina data.

PacBio assembly

Raw PacBio reads were assembled using the MARVEL assembler [26, 27] with default parameters unless mentioned otherwise. MARVEL consists of three major steps, namely the

setup phase, patch phase and the assembly phase. In the setup phase, reads were filtered by choosing only the best read of each ZMW and requiring subsequently a minimum read length of 2 Kb. The resulting 7.9 million reads (27.35X coverage) were stored in an internal database. The patch phase detects and corrects read artifacts including missed adapters, polymerase strand jumps, chimeric reads and long low-quality read segments that are the primary impediments to long contiguous assemblies. The patched reads (24.6X coverage) were then used for the final assembly phase, which stitches short alignment artifacts resulting from bad sequencing segments within overlapping read pairs. This step is followed by repeat annotation and the generation of the overlap graph. To this end, we used the tool LAq with a quality cutoff of 35 to calculate a quality and a trim annotation track. In addition, alignments were forced through low quality regions (<200 bp) that remained in the patched reads. LArepeat in coverage auto detection mode was used to create a repeat annotation track based on overlap coverage anomalies. The final assembled contigs are generated by touring the overlap graph. To correct base errors, we first used the correction module of MARVEL, which makes use of the final overlap graph and corrects only the reads that were used to build the contigs. Corrected contigs were further polished using PacBio's Quiver tool [48].

Merging Illumina and PacBio assemblies

We used quickmerge [30] to combine the improved Illumina and PacBio assemblies. Quickmerge was run in two rounds. In the first round, we used the improved Illumina assembly as query and the PacBio assembly as reference, specifying the '-*l*' parameter to the scaffold N50 of the reference assembly. In the second round, we again used the improved Illumina assembly as query but the resulting assembly from round 1 as Reference (again setting the '–*l*' parameter to the N50 of the reference assembly).

Optical map

A genome map was assembled *de novo* and used to order and orient the scaffolds from the quickmerged Illumina-PacBio assembly, and to correct contig misassemblies. Consensus physical maps (CMAP) were assembled using Bionano Access 1.1.2 and Bionano Solve 3.2. Molecules were filtered for minimum length of 100 Kb, minimum of eight labels on each molecule, and a backbone intensity of maximum 0.45 (n = 835772; approximately 89X raw coverage). A P-value threshold for the optical mapping assembly was set to at least $1x10^{-10}$. A total of 2,742 CMAPs (N50 of 1.052 Mb; total CMAP length of 2141.075 Mb) were generated.

Hybrid scaffolding

We used the Bionano Access 1.1.2/Bionano Solve 3.2 hybrid-scaffolding pipeline, with input parameters optimized for human (see Bionano Genomics 'Hybrid Scaffolding Theory of Operation' for a detailed explanation and summary of all input parameters [49]). In short, the process of hybrid scaffolding includes alignment of the Illumina-PacBio assembly against the Bionano physical maps, identifying and resolving conflicting alignments, merging of non-conflicting assembly and CMAPs into hybrid scaffolds, and the final translation back to fasta format.

Final assembly polishing

To correct remaining base errors, we used the variant detector FreeBayes (FreeBayes, RRID:SCR_010761)[50] and bcftools consensus [51] with a score cutoff of 1, to detect and correct erroneous or polymorphic positions in the assembly. Of a total of 6,322,937 assembly positions where the base identity was changed (0.3% of the genome), 82.8% correspond to heterozygous positions and 17.2% correspond to erroneous base calls in the original assembly.

Obtaining per-base quality values

We used bcftools (SAMtools/BCFtools, RRID:SCR_005227)[52] with the Illumina sequencing reads (parameters '*bcftools mpileup -A* | *bcftools call -c*') to obtain a quality value for each base in the assembly where a read is mapped to. Overall, 99.8% of the bases in the tegu v2 assembly have a Phred quality score greater than 40, which corresponds to a base accuracy of 99.99%.

K-mer size estimation

We used genomescope [53] to obtain a k-mer based estimate of the size of the tegu genome. We used the Illumina sequencing reads and the default k-mer size of 21 bp, and obtained a minimum-to-maximum estimate of 1.904 to 1.905 Gb.

Comparison of the tegu v1 and v2 assemblies

To analyze the sequence similarity between both assemblies, we aligned the v2 assembly to the v1 assembly as described previously [54], but with lastz [55] parameters '--*gappedthresh=8000 --hspthresh=4000'*. Then we determined the sequence identity in all aligning regions (note that no comparison can be made in assembly gap regions that were closed in v2). This showed that 99.83% of the bases in the v1 assembly are unchanged in v2 and that both assemblies differ in 0.12% substitutions, 0.04% insertions and 0.0028% deletions. Inspecting the differences and the aligned Illumina reads showed that almost all differences between both assemblies correspond to polymorphisms, where reads support both variants and the assembly polishing step changed identity of the variant.

Transcriptome assembly

We first trimmed the raw sequencing reads for the presence of sequencing adapters with cutadapt (cutadapt, RRID:SCR_011841)[45] (v1.5), setting a minimum read length of 30 bp, and then mapped the trimmed reads against the tegu lizard genome using HISAT2 (HiSat2, RRID:SCR_015530)[56] (v2.1.0, parameters '*--rna-strandness RF*'). We assembled the mapped reads using Cufflinks (Cufflinks, RRID:SCR_014597)[57](v2.2.1, parameters '--

library-type fr-firststrand'), resulting in 63,127 transcripts, and also using Trinity (Trinity , RRID:SCR_013048)[58](v2.3.2, parameters '*--SS_lib_type RF --genome_guided_max_intron 20000*'), resulting in 481,835 transcripts. Next, we applied PASA (PASA, RRID:SCR_014656)[59](v.2.3.0, default parameters) to map the assembled transcripts to the genome with BLAT. PASA also removed low-quality alignments (alignment identity less than 95% and minimum of 75% aligned) and combined both trinity and cufflinks transcripts by collapsing redundant transcripts and clustering transcripts that have overlapping exons on the same strand. This resulted in 304,367 transcripts.

Assessing assembly completeness

We assessed completeness of the tegu lizard genome assembly and compared it to the genomes of other reptiles by quantifying both the number of conserved genes and non-exonic genomic regions found in each genome. For genes, we ran BUSCO (BUSCO , RRID:SCR_015008)[31](v3.0.2) on genome mode to search for genes conserved in vertebrate and tetrapod species (vertebrata_odb9 and tetrapoda_odb9 gene databases, created on 2016-02-13). The vertebrata database consists of 2,586 genes, and the tetrapoda database consists of 3,950 genes.

We further assessed assembly completeness using two sets of non-exonic regions that are highly conserved among vertebrates. First, as previously described [26], we selected a set of 197 Ultra-Conserved Elements (UCEs; genomic regions equal or greater than 200 bp that are identical between human, mouse and rat [32] that are also conserved in chicken, zebrafish, and medaka and that do not overlap exons (based on human hg38 ensGene table from UCSC genome browser). Second, we obtained CNEs that are well conserved among mammals and teleost fish, and also align to shark and lamprey from [33]. To ensure that CNEs can be easily found in a genome if the CNE sequence is present, we focused only on those CNEs that are longer than 300 bp. Furthermore, we removed 30 bp from both ends as often the CNE core is conserved among large evolutionary distances. This resulted in a set

of 493 CNEs. Of these, 282 pairs of CNEs are neighbors located on the same chromosome and at most 1 Mb from each other in the human, mouse and chicken genome, and thus are evolutionarily conserved neighbors. Both UCE and CNE sets were mapped to the genome using lastz [55](v1.02.00, parameters '*--gappedthresh=3000 --hspthresh=2500 --seed=match6 --format=general*'). We further filtered these mappings for ≥60% alignment identity and ≥80% alignment coverage. The UCE/CNE sequences are provided as fasta files at https://bds.mpi-cbg.de/hillerlab/TeguGenomeData/ as a resource for further vertebrate assembly completeness assessments.

Repeat annotation

We used RepeatModeler (RepeatModeler, RRID:SCR_015027)[60](v1.0.8, parameters '*-engine ncbi*') to *de novo* identify repeat families in the tegu genome. Then, we used RepeatMasker (RepeatMasker , RRID:SCR_012954)(v4.0.5, default parameters) with the resulting repeat library to soft-mask the tegu genome, and ran Tandem Repeat Finder [61]to annotate simple and tandem repeats. We applied the same procedure to the genomes of all other analyzed squamates.

Gene annotation

In order to annotate genes in the tegu genome, we prepared the following four evidence-based datasets: First, we used our assembled PASA transcripts, which were passed to MAKER via the est_gff option in the maker_opts.ctl file. Second, we downloaded protein sequences available on UNIPROT (data accessed in March/April.2018; 20 lizard species, 9 snake species, chicken, softshell turtle, and two alligator species; Supplementary Table 7). We only kept those proteins with strong experimental evidence (sequences annotated with PE=1 or PE=2), resulting in a total of 3,739 protein sequences. We mapped these sequences to the tegu v2 genome with exonerate [37] (v2.2.20, parameters '*-m protein2genome --subopt 0 -M 20000 -D 2000 --minintron 20 --maxintron 50000 --softmasktarget T --proteinhspdropoff 20 --exhaustive no --refine region --bestn 1*'). This

resulted in 3,637 mappings for 3,607 proteins, which were passed to MAKER via the protein_gff option in the maker_opts.ctl file. Third, we mapped human genes to the tegu lizard genome with CESAR [38, 39]. We selected 20,145 transcripts corresponding to the longest isoform of human Ensembl genes downloaded from UCSC genome browser (hg38 ensGene table), and, based on our pairwise whole genome alignment (below), annotated exons with an intact open reading frame and consensus splice sites in the tegu lizard genome. We filtered out mappings corresponding to single-exon genes that were smaller than 100 bp, and mappings spanning more than 10 Mb. This resulted in 16,995 mappings which were passed to MAKER via the model_gff option in the maker_opts.ctl file. Fourth, we ran BRAKER [40] with the HISAT2-mapped reads and the gene annotation of the v1 assembly version [21] as input. We filtered the 81,625 gene predictions from BRAKER to eliminate short, low-scoring overlapping genes, resulting in 75,444 predictions which were passed to MAKER via the pred_gff option in the maker_opts.ctl file. In addition to evidence-based datasets, we also used *de novo* gene prediction using Augustus [62] with a previously-obtained gene model [21] and specified the MAKER augustus_species option in the maker_opts.ctl file.

We ran MAKER [36](v2.31.9), setting *est2genome* and *protein2genome=1*, *max_dna_len=300000*, *min_contig=100*, *always_complete=1*, *keep_preds=0*, *split_hit=10000*, *single_exon=1*, *single_length=150*, *correct_est_fusion=1*, and *alt_splice=0*.

Multiple genome alignment

We first computed pairwise genome alignments between tegu and other reptiles and amniotes using the lastz/chain/net pipeline, as described in [33, 54]. To this end, we used lastz [55] (v1.04.00) with alignment parameters '*K=2200 L=3000 Y=9400 H=2000*' and the default scoring matrix for aligning reptile species to the tegu genome. The same parameters were used to align non-squamate species, except that we set *Y=3400* and used the HoxD55 scoring matrix. We next built co-linear alignment chains with axtChain [63] using default

parameters and applied chainCleaner [64] (parameters *-LRfoldThreshold=2.5 -doPairs -LRfoldThresholdPairs=10 -maxPairDistance=10000 -maxSuspectScore=100000 -minBrokenChainScore=75000*) to improve alignment specificity. For non-squamate species, which are separated from the tegu by >0.72 neutral substitutions per site, we subsequently ran an additional round of highly sensitive local alignments with lastz to uncover additional alignments that were missed before. To this end, we used the parameters '*K=1500 L=2500 W=5*' on all non-aligning regions flanked by local alignments in the chains that are between 20 bp and 100 Kb long. As shown in [33, 54], this procedure is able to uncover numerous additional alignments to exons and CNEs. All local alignments were quality-filtered by requiring that each alignment contains at least one ≥30 bp region with ≥60% sequence identity and ≥1.8 bits entropy as described in [33]. We then generated alignment nets from the chains using chainNet [63] with default parameters. We removed low-scoring alignment nets that are unlikely to represent real homologies by running a non-nested filtering procedure that keeps only nets that span ≥4 Kb in both genomes and have a score ≥20,000. Nets that represent inversions or local translocations and have a score ≥10,000 were also kept. Finally, we used Multiz [65] to produce a multiple alignment from all filtered pairwise alignment nets. The phylogenetic position of the squamate species was taken from [66]. We estimated neutral branch lengths in the phylogenetic tree using phyloFit [41] with parameters '*--EM --precision HIGH --subst-mod REV*' and 4-fold degenerated third codon positions based on our gene annotation.

Annotating conserved regions

To detect genomic regions that are under evolutionary constraint, we applied PhastCons [41] (parameters '*expected-length=45, target-coverage=0.3 rho=0.3*') and GERP (GERP, RRID:SCR_000563)[42] (default parameters) to our multiple alignment using the phylogenetic tree with neutral branch lengths. We merged both PhastCons and GERP sets of conserved regions, joined those regions separated by ≤10 bp and filtered the resulting

ones for a minimum size of 30 bp. Finally, we only kept conserved regions that align well to at least 4 of the 9 non-tegu squamates in the tegu-based alignment.

To obtain conserved non-exonic elements (CNEs), we excluded all bases from the full set of conserved elements that overlap exons in our CESAR or MAKER gene annotation. Specifically, we subtracted exonic bases from all bases in conserved elements and required that the resulting CNEs are at least 30 bp long.

We defined two subsets of CNEs, a squamate-specific set and a not squamate-specific one, based on well-aligning regions in other species. For each species in the multiple alignment, we determined all windows of ≥30 bp where the alignment identity is ≥60%. To define the squamate-specific subset, we selected those CNEs that overlap these aligning windows in at least 6 of the 9 squamates and not a single non-squamate amniote. To define the not squamate-specific subset, we selected those CNEs that overlap aligning windows in at least 6 of the 9 squamates and overlap aligning windows in at least one non-squamate amniote. To determine the overlap between CNEs and transposons, we considered SINE, LINE, LTR and DNA transposons from our RepeatMasker annotation and extracted CNEs that overlap transposons for at least 30 bp.

Data availability

All raw sequencing data and genome assemblies are available at NCBI under the project accession number PRJNA473319. All other data, including annotated genes, the multiple genome alignment, and conserved element datasets are available at https://bds.mpi-cbg.de/hillerlab/TeguGenomeData/. The genome and its annotations can also be loaded into the UCSC genome browser as an assembly hub (URL https://bds.mpi-cbg.de/hillerlab/TeguGenomeData/assemblyHub/hub.txt). Optical map, annotation and tree data are also available from the *GigaScience* GigaDB repository [67].

Acknowledgments

We would like to thank the Computer Service Facilities of the MPI-CBG and MPI-PKS, and the Scientific Computing Facility of the MPI-CBG for their support, and Thomas Hackl and Peter Steinbach for help with error correction. This work was supported by the Max Planck Society, by FAPESP stipends 2012/01319-8 and 2012/23360 to JGR, and by the Tschira foundation.

References

1. Uetz P: The reptile database. http://www.reptile-database.org. Accessed march.2018.
2. Alfoldi J, Di Palma F, Grabherr M, Williams C, Kong L, Mauceli E, et al. The genome of the green anole lizard and a comparative analysis with birds and mammals. Nature. 2011;477 7366:587-91. doi:10.1038/nature10390.
3. Bradnam KR, Fass JN, Alexandrov A, Baranay P, Bechner M, Birol I, et al. Assemblathon 2: evaluating de novo methods of genome assembly in three vertebrate species. Gigascience. 2013;2 1:10. doi:10.1186/2047-217X-2-10.
4. Castoe TA, de Koning AP, Hall KT, Card DC, Schield DR, Fujita MK, et al. The Burmese python genome reveals the molecular basis for extreme adaptation in snakes. Proc Natl Acad Sci U S A. 2013;110 51:20645-50. doi:10.1073/pnas.1314475110.
5. Crotalus genome. https://www.ncbi.nlm.nih.gov/assembly/727941. Accessed feb.2018.
6. Gilbert C, Meik JM, Dashevsky D, Card DC, Castoe TA and Schaack S. Endogenous hepadnaviruses, bornaviruses and circoviruses in snakes. Proc Biol Sci. 2014;281 1791:20141122. doi:10.1098/rspb.2014.1122.
7. Vonk FJ, Casewell NR, Henkel CV, Heimberg AM, Jansen HJ, McCleary RJ, et al. The king cobra genome reveals dynamic gene evolution and adaptation in the snake venom system. Proc Natl Acad Sci U S A. 2013;110 51:20651-6. doi:10.1073/pnas.1314702110.
8. Castoe TA, Bronikowski AM, Brodie ED, 3rd, Edwards SV, Pfrender ME, Shapiro MD, et al. A proposal to sequence the genome of a garter snake (Thamnophis sirtalis). Stand Genomic Sci. 2011;4 2:257-70. doi:10.4056/sigs.1664145.
9. Ullate-Agote A, Milinkovitch MC and Tzika AC. The genome sequence of the corn snake (Pantherophis guttatus), a valuable resource for EvoDevo studies in squamates. Int J Dev Biol. 2014;58 10-12:881-8. doi:10.1387/ijdb.150060at.
10. Aird SD, Arora J, Barua A, Qiu L, Terada K and Mikheyev AS. Population Genomic Analysis of a Pitviper Reveals Microevolutionary Forces Underlying Venom Chemistry. Genome Biol Evol. 2017;9 10:2640-9. doi:10.1093/gbe/evx199.
11. viper. https://www.ncbi.nlm.nih.gov/assembly/233891.
12. Liu Y, Zhou Q, Wang Y, Luo L, Yang J, Yang L, et al. Gekko japonicus genome reveals evolution of adhesive toe pads and tail regeneration. Nat Commun. 2015;6:10033. doi:10.1038/ncomms10033.
13. Xiong Z, Li F, Li Q, Zhou L, Gamble T, Zheng J, et al. Draft genome of the leopard gecko, Eublepharis macularius. Gigascience. 2016;5 1:47. doi:10.1186/s13742-016-0151-4.
14. Song B, Cheng S, Sun Y, Zhong X, Jin J, Guan R, et al. A genome draft of the legless anguid lizard, Ophisaurus gracilis. Gigascience. 2015;4:17. doi:10.1186/s13742-015-0056-7.
15. Georges A, Li Q, Lian J, O'Meally D, Deakin J, Wang Z, et al. High-coverage sequencing and annotated assembly of the genome of the Australian dragon lizard Pogona vitticeps. Gigascience. 2015;4:45. doi:10.1186/s13742-015-0085-2.
16. Gao J, Li Q, Wang Z, Zhou Y, Martelli P, Li F, et al. Sequencing, de novo assembling, and annotating the genome of the endangered Chinese crocodile lizard Shinisaurus crocodilurus. Gigascience. 2017;6 7:1-6. doi:10.1093/gigascience/gix041.
17. Ávila-Pires TC. Lizards of the brazilian Amazon (Reptilia:Squamata). Zool Verhandelingen (Leiden). 1995;299:706.
18. Presch W. A review of the tegu lizards genus Tupinambis (Sauria: Teiidae) from south America. Copeia. 1973;4:6.
19. Péres J. *Sistemática e conservação de lagartos do gênero Tupinambis (Squamata, Teiidae)*. Universidade de Brasília, 2003.
20. Mazzotti FJMMRMRRNEJKVJEJW, J. Tupinambis merianae as nest predators of crocodilians and turtles in Florida, USA. . Biological Invasions. 2015;17:3.

21. Roscito JG, Sameith K, Parra G, Langer BE, Petzold A, Moebius C, Bickle M, Rodrigues MT, Hiller M. Phenotype loss is associated with widespread divergence of the gene regulatory landscape in evolution. Nat Commun. 2018 Nov 9;9(1):4737. doi: 10.1038/s41467-018-07122-z.
22. Gnerre S, Maccallum I, Przybylski D, Ribeiro FJ, Burton JN, Walker BJ, et al. High-quality draft assemblies of mammalian genomes from massively parallel sequence data. Proc Natl Acad Sci U S A. 2011;108 4:1513-8. doi:10.1073/pnas.1017351108.
23. Hackl T, Hedrich R, Schultz J and Forster F. Proovread: large-scale high-accuracy PacBio correction through iterative short read consensus. Bioinformatics. 2014;30 21:3004-11. doi:10.1093/bioinformatics/btu392.
24. Kosugi S, Hirakawa H and Tabata S. GMcloser: closing gaps in assemblies accurately with a likelihood-based selection of contig or long-read alignments. Bioinformatics. 2015;31 23:3733-41. doi:10.1093/bioinformatics/btv465.
25. Boetzer M, Henkel CV, Jansen HJ, Butler D and Pirovano W. Scaffolding pre-assembled contigs using SSPACE. Bioinformatics. 2011;27 4:578-9. doi:10.1093/bioinformatics/btq683.
26. Nowoshilow S, Schloissnig S, Fei JF, Dahl A, Pang AWC, Pippel M, et al. The axolotl genome and the evolution of key tissue formation regulators. Nature. 2018;554 7690:50-5. doi:10.1038/nature25458.
27. Grohme MA, Schloissnig S, Rozanski A, Pippel M, Young GR, Winkler S, et al. The genome of Schmidtea mediterranea and the evolution of core cellular mechanisms. Nature. 2018;554 7690:56-61. doi:10.1038/nature25473.
28. pacbio github page. https://github.com/PacificBiosciences/Bioinformatics-Training/wiki/Large-Genome-Assembly-with-PacBio-Long-Reads.
29. Berlin K, Koren S, Chin CS, Drake JP, Landolin JM and Phillippy AM. Assembling large genomes with single-molecule sequencing and locality-sensitive hashing. Nat Biotechnol. 2015;33 6:623-30. doi:10.1038/nbt.3238.
30. Chakraborty M, Baldwin-Brown JG, Long AD and Emerson JJ. Contiguous and accurate de novo assembly of metazoan genomes with modest long read coverage. Nucleic Acids Res. 2016;44 19:e147. doi:10.1093/nar/gkw654.
31. Simao FA, Waterhouse RM, Ioannidis P, Kriventseva EV and Zdobnov EM. BUSCO: assessing genome assembly and annotation completeness with single-copy orthologs. Bioinformatics. 2015;31 19:3210-2. doi:10.1093/bioinformatics/btv351.
32. Bejerano G, Pheasant M, Makunin I, Stephen S, Kent WJ, Mattick JS, et al. Ultraconserved elements in the human genome. Science. 2004;304 5675:1321-5. doi:10.1126/science.1098119.
33. Hiller M, Agarwal S, Notwell JH, Parikh R, Guturu H, Wenger AM, et al. Computational methods to detect conserved non-genic elements in phylogenetically isolated genomes: application to zebrafish. Nucleic Acids Res. 2013;41 15:e151. doi:10.1093/nar/gkt557.
34. Woolfe A, Goodson M, Goode DK, Snell P, McEwen GK, Vavouri T, et al. Highly conserved non-coding sequences are associated with vertebrate development. PLoS Biol. 2005;3 1:e7. doi:10.1371/journal.pbio.0030007.
35. Visel A, Prabhakar S, Akiyama JA, Shoukry M, Lewis KD, Holt A, et al. Ultraconservation identifies a small subset of extremely constrained developmental enhancers. Nat Genet. 2008;40 2:158-60. doi:10.1038/ng.2007.55.
36. Cantarel BL, Korf I, Robb SM, Parra G, Ross E, Moore B, et al. MAKER: an easy-to-use annotation pipeline designed for emerging model organism genomes. Genome Res. 2008;18 1:188-96. doi:10.1101/gr.6743907.
37. Slater GS and Birney E. Automated generation of heuristics for biological sequence comparison. BMC Bioinformatics. 2005;6:31. doi:10.1186/1471-2105-6-31.
38. Sharma V, Elghafari A and Hiller M. Coding exon-structure aware realigner (CESAR) utilizes genome alignments for accurate comparative gene annotation. Nucleic Acids Res. 2016;44 11:e103. doi:10.1093/nar/gkw210.

39. Sharma V, Schwede P and Hiller M. CESAR 2.0 substantially improves speed and accuracy of comparative gene annotation. Bioinformatics. 2017;33 24:3985-7. doi:10.1093/bioinformatics/btx527.
40. Hoff KJ, Lange S, Lomsadze A, Borodovsky M and Stanke M. BRAKER1: Unsupervised RNA-Seq-Based Genome Annotation with GeneMark-ET and AUGUSTUS. Bioinformatics. 2016;32 5:767-9. doi:10.1093/bioinformatics/btv661.
41. Siepel A, Bejerano G, Pedersen JS, Hinrichs AS, Hou M, Rosenbloom K, et al. Evolutionarily conserved elements in vertebrate, insect, worm, and yeast genomes. Genome Res. 2005;15 8:1034-50. doi:10.1101/gr.3715005.
42. Davydov EV, Goode DL, Sirota M, Cooper GM, Sidow A and Batzoglou S. Identifying a high fraction of the human genome to be under selective constraint using GERP++. PLoS Comput Biol. 2010;6 12:e1001025. doi:10.1371/journal.pcbi.1001025.
43. Nguyen N, Hickey G, Raney BJ, Armstrong J, Clawson H, Zweig A, et al. Comparative assembly hubs: web-accessible browsers for comparative genomics. Bioinformatics. 2014;30 23:3293-301. doi:10.1093/bioinformatics/btu534.
44. Mikkelsen TS, Wakefield MJ, Aken B, Amemiya CT, Chang JL, Duke S, et al. Genome of the marsupial Monodelphis domestica reveals innovation in non-coding sequences. Nature. 2007;447 7141:167-77. doi:10.1038/nature05805.
45. Martin M. Cutadapt removes adapter sequences from high-throughput sequencing reads. EMBnetjournal Bioinformatics in Action 2011;17.
46. Sameith K, Roscito JG and Hiller M. Iterative error correction of long sequencing reads maximizes accuracy and improves contig assembly. Brief Bioinform. 2015;18 1:1-8. doi:10.1093/bib/bbw003.
47. Luo R, Liu B, Xie Y, Li Z, Huang W, Yuan J, et al. SOAPdenovo2: an empirically improved memory-efficient short-read de novo assembler. Gigascience. 2012;1 1:18. doi:10.1186/2047-217X-1-18.
48. Quiver. https://github.com/PacificBiosciences/GenomicConsensus.
49. Bionano genomics. www.bionanogenomics.com.
50. Freebayes. https://github.com/ekg/freebayes.
51. bcftools. https://samtools.github.io/bcftools/bcftools.html.
52. Li H. A statistical framework for SNP calling, mutation discovery, association mapping and population genetical parameter estimation from sequencing data. Bioinformatics. 2011;27 21:2987-93. doi:10.1093/bioinformatics/btr509.
53. Vurture GW, Sedlazeck FJ, Nattestad M, Underwood CJ, Fang H, Gurtowski J, et al. GenomeScope: fast reference-free genome profiling from short reads. Bioinformatics. 2017;33 14:2202-4. doi:10.1093/bioinformatics/btx153.
54. Sharma V and Hiller M. Increased alignment sensitivity improves the usage of genome alignments for comparative gene annotation. Nucleic Acids Res. 2017;45 14:8369-77. doi:10.1093/nar/gkx554.
55. Harris RS. *Improved pairwise alignment of genomic DNA.* The Pennsylvania State University, , 2007.
56. Kim D, Langmead B and Salzberg SL. HISAT: a fast spliced aligner with low memory requirements. Nat Methods. 2015;12 4:357-60. doi:10.1038/nmeth.3317.
57. Trapnell C, Williams BA, Pertea G, Mortazavi A, Kwan G, van Baren MJ, et al. Transcript assembly and quantification by RNA-Seq reveals unannotated transcripts and isoform switching during cell differentiation. Nat Biotechnol. 2010;28 5:511-5. doi:10.1038/nbt.1621.
58. Grabherr MG, Haas BJ, Yassour M, Levin JZ, Thompson DA, Amit I, et al. Full-length transcriptome assembly from RNA-Seq data without a reference genome. Nat Biotechnol. 2011;29 7:644-52. doi:10.1038/nbt.1883.
59. Haas BJ, Delcher AL, Mount SM, Wortman JR, Smith RK, Jr., Hannick LI, et al. Improving the Arabidopsis genome annotation using maximal transcript alignment assemblies. Nucleic Acids Res. 2003;31 19:5654-66.
60. Repeat masker. http://www.repeatmasker.org/.
61. Tandem repeat finder. https://tandem.bu.edu/trf/trf.html.

62. Stanke M and Waack S. Gene prediction with a hidden Markov model and a new intron submodel. Bioinformatics. 2003;19 Suppl 2:ii215-25.

63. Kent WJ, Baertsch R, Hinrichs A, Miller W and Haussler D. Evolution's cauldron: duplication, deletion, and rearrangement in the mouse and human genomes. Proc Natl Acad Sci U S A. 2003;100 20:11484-9. doi:10.1073/pnas.1932072100.

64. Suarez HG, Langer BE, Ladde P and Hiller M. chainCleaner improves genome alignment specificity and sensitivity. Bioinformatics. 2017;33 11:1596-603. doi:10.1093/bioinformatics/btx024.

65. Blanchette M, Kent WJ, Riemer C, Elnitski L, Smit AF, Roskin KM, et al. Aligning multiple genomic sequences with the threaded blockset aligner. Genome Res. 2004;14 4:708-15. doi:10.1101/gr.1933104.

66. Pyron RA, Burbrink FT and Wiens JJ. A phylogeny and revised classification of Squamata, including 4161 species of lizards and snakes. BMC Evol Biol. 2013;13:93. doi:10.1186/1471-2148-13-93.

67. Roscito JG; Sameith K; Pippel M; Francoijs K; Winkler S; Dahl A; Papoutsoglou G; Myers G; Hiller M (2018): Supporting data for "The genome of the tegu lizard Salvator merianae: combining Illumina, PacBio, and optical mapping data to generate a highly contiguous assembly" GigaScience Database. http://dx.doi.org/10.5524/100529

68. Irisarri I, Baurain D, Brinkmann H, Delsuc F, Sire JY, Kupfer A, et al. Phylotranscriptomic consolidation of the jawed vertebrate timetree. Nat Ecol Evol. 2017;1 9:1370-8. doi:10.1038/s41559-017-0240-5.

Figures

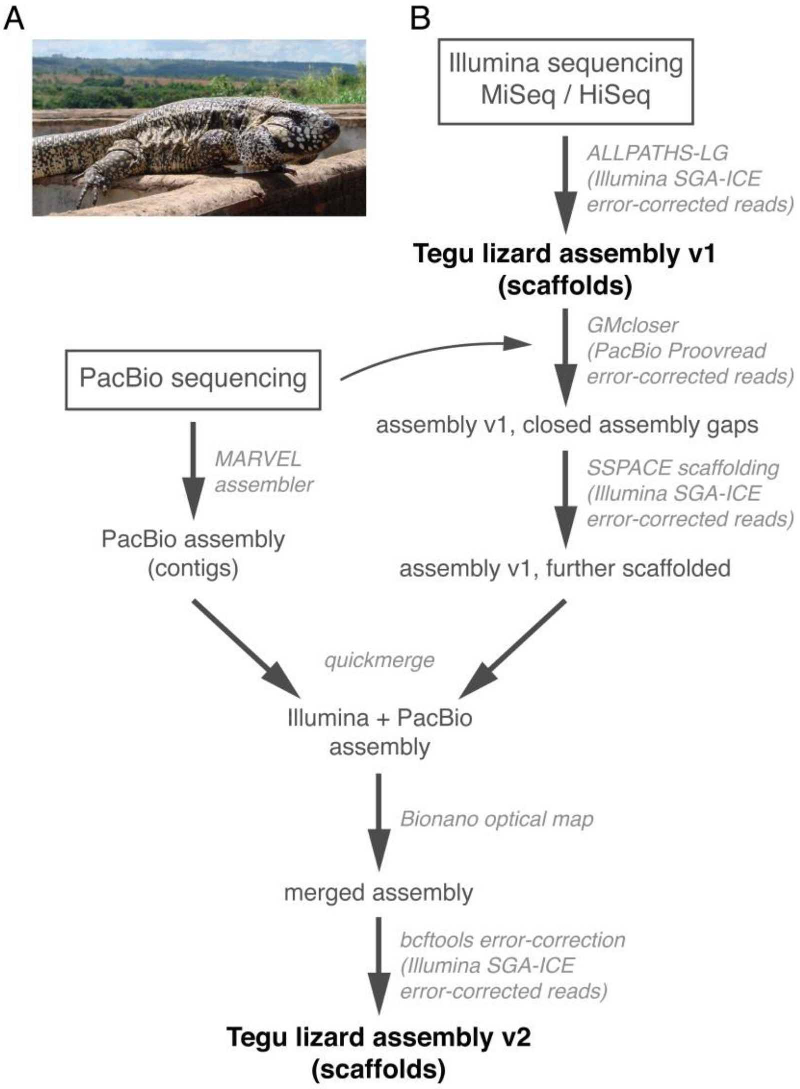

Figure 1: Workflow to generate the tegu lizard v2 assembly.

(A) The tegu lizard *Salvator merianae.*

(B) Assembly v1 was built entirely from Illumina short read data. To improve this assembly, we used PacBio long read data to close assembly gaps and extend scaffolds, and merged the improved Illumina with a PacBio-only assembly. Finally, optical mapping data was used to resolve contig chimeras and scaffold even further. Used tools and their input data are shown in grey.

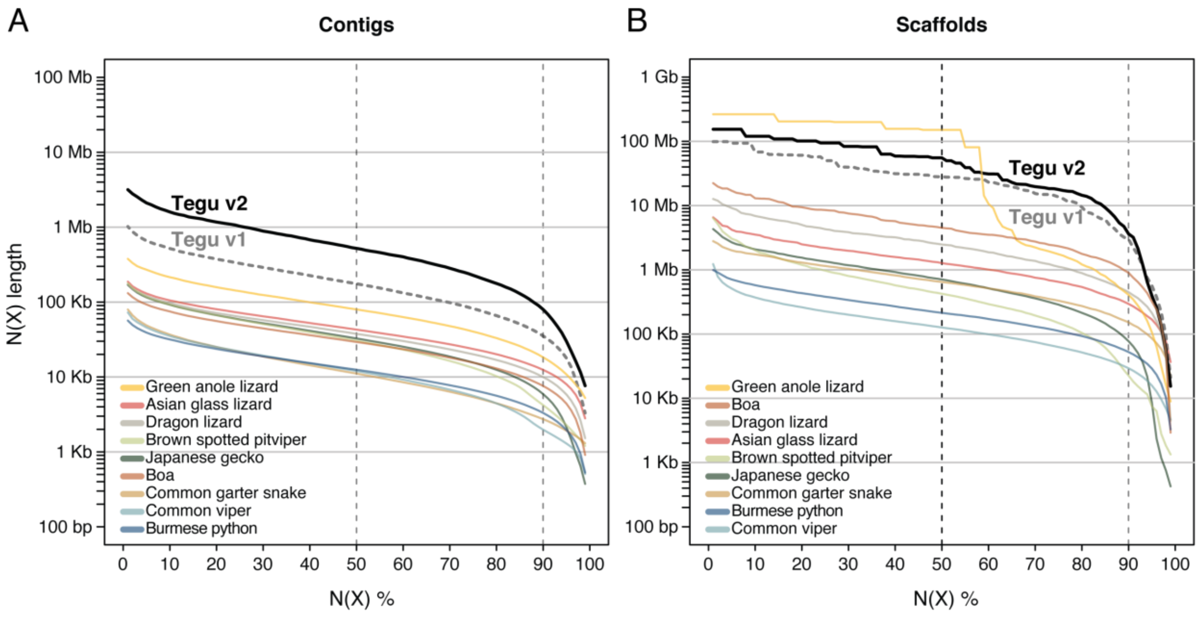

Figure 2: Comparison of assembly contiguity.

N(x)% graphs show the contig (A) and scaffold (B) sizes (y-axis), where x% of the genome assembly consists of contigs and scaffolds of at least that size. The tegu lizard v1 and v2 assemblies are shown in grey and black. All other assemblies are sorted by the N50 values in the insets. Dashed lines mark the N50 and N90 values.

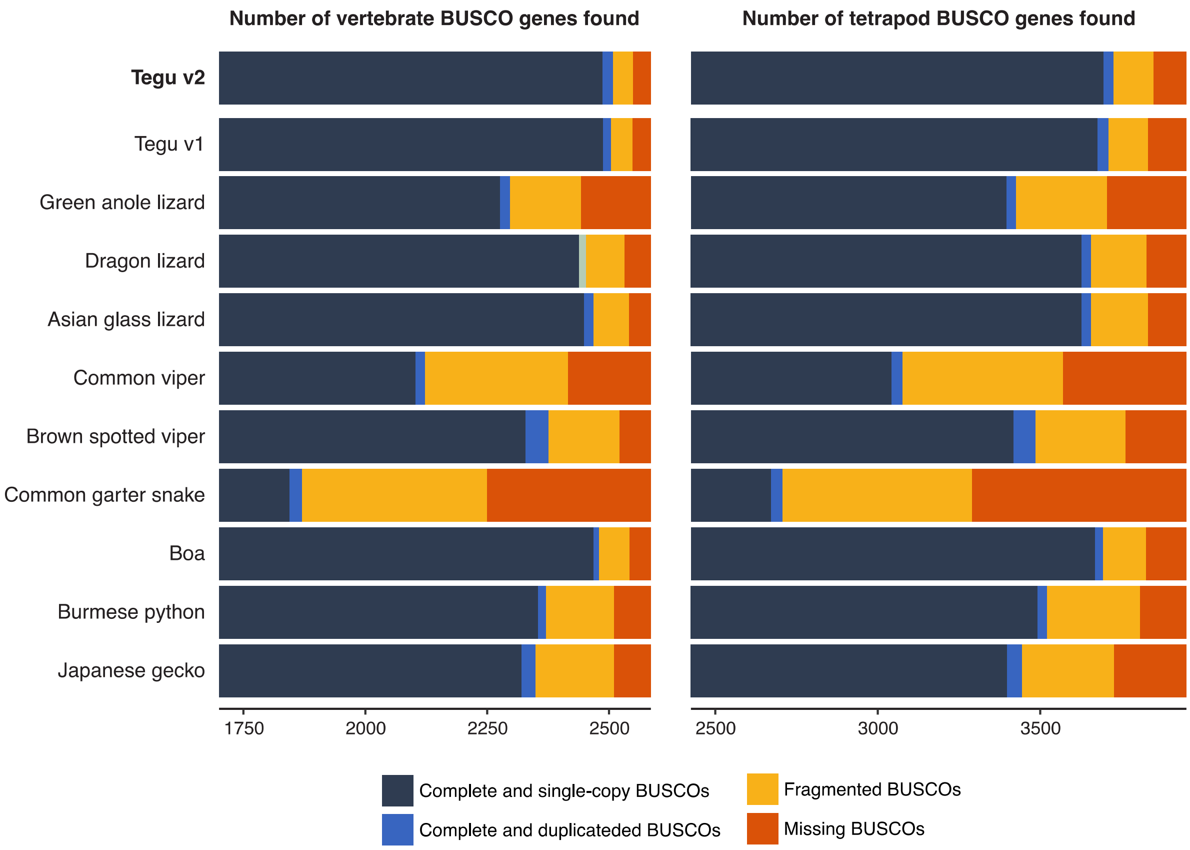

Figure 3: Comparison of genome completeness for coding genes.

The bar charts show the number of complete, fragmented and missing genes using two BUSCO datasets for vertebrate-conserved (left) and tetrapod-conserved (right) genes.

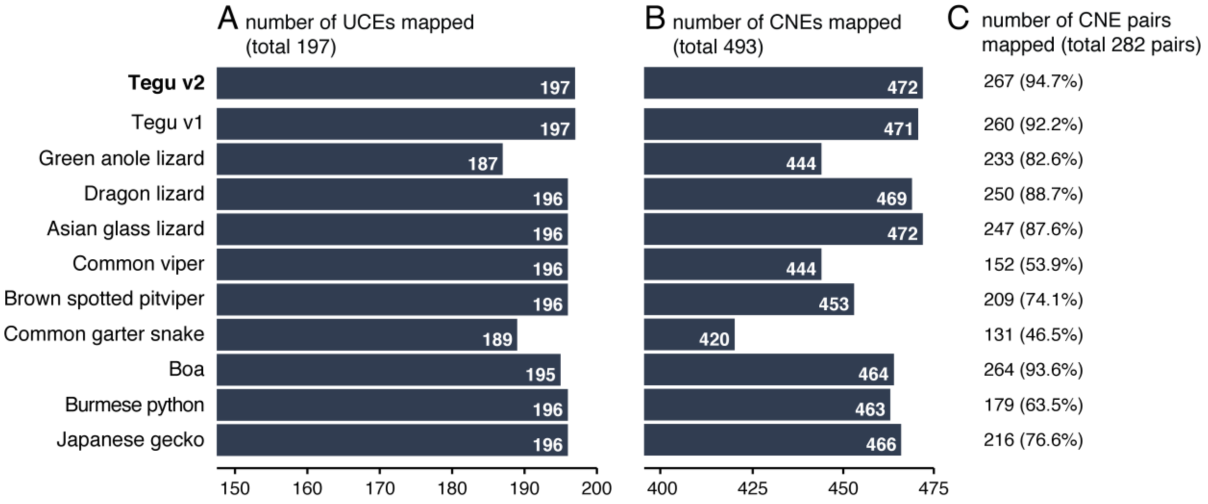

Figure 4: Using conserved non-exonic elements to compare genome completeness and contiguity.

Bar charts show (A) the number of aligning Ultra-Conserved Elements that do not overlap coding regions (UCEs, N=197 in total) and (B) the number of aligning conserved elements that do not overlap exons (CNEs, N=493 in total). (C) The percentage of 282 evolutionarily-conserved pairs of neighboring CNEs that are also found as neighbors in the squamate assemblies. Both UCE and CNE sets are highly conserved among vertebrates and thus are likely to exist in squamates.

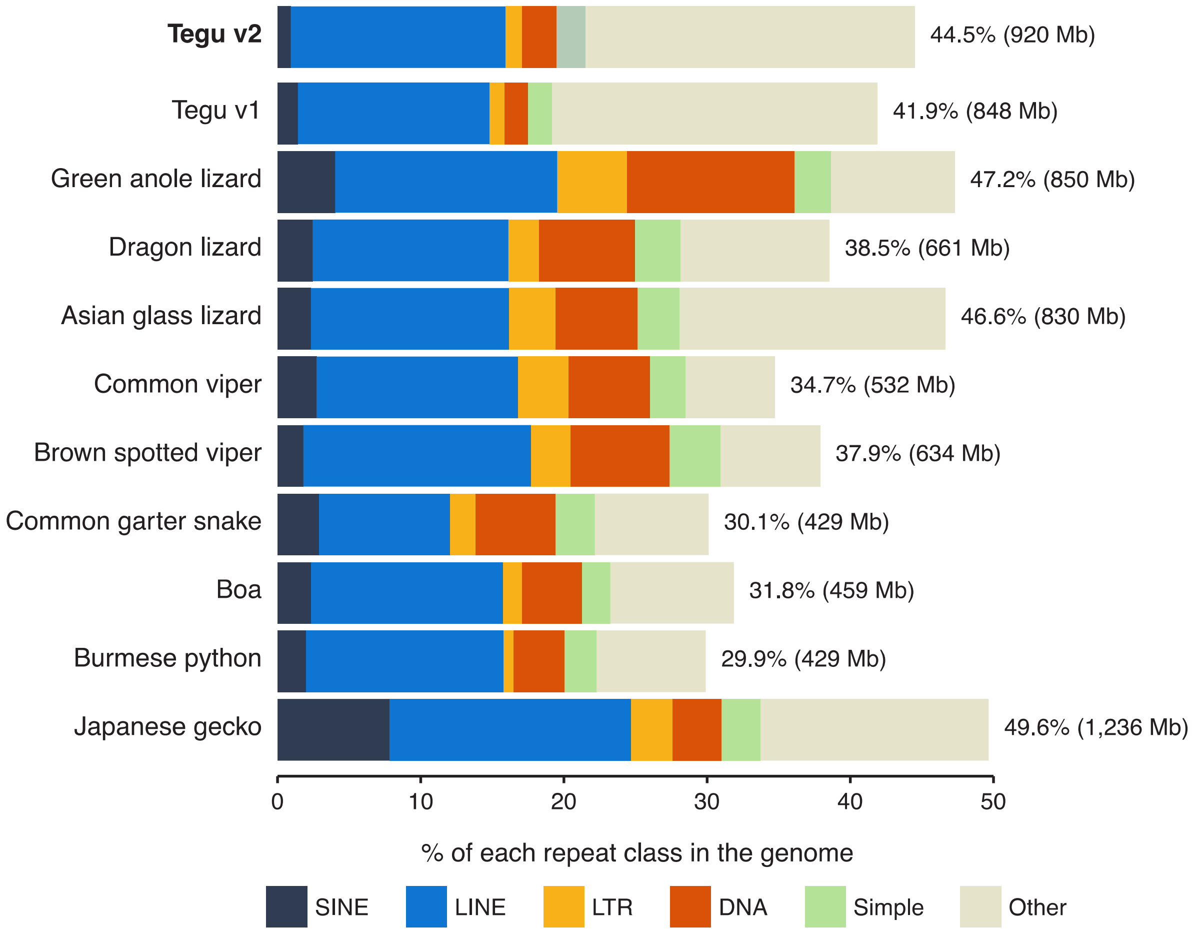

Figure 5: Repeat landscape in squamate genomes.

Major classes of repeats are color-coded and shown as bar charts that represent the portion of the genome they cover. Simple repeats comprise tandem repeats, low complexity regions and satellite repeats.

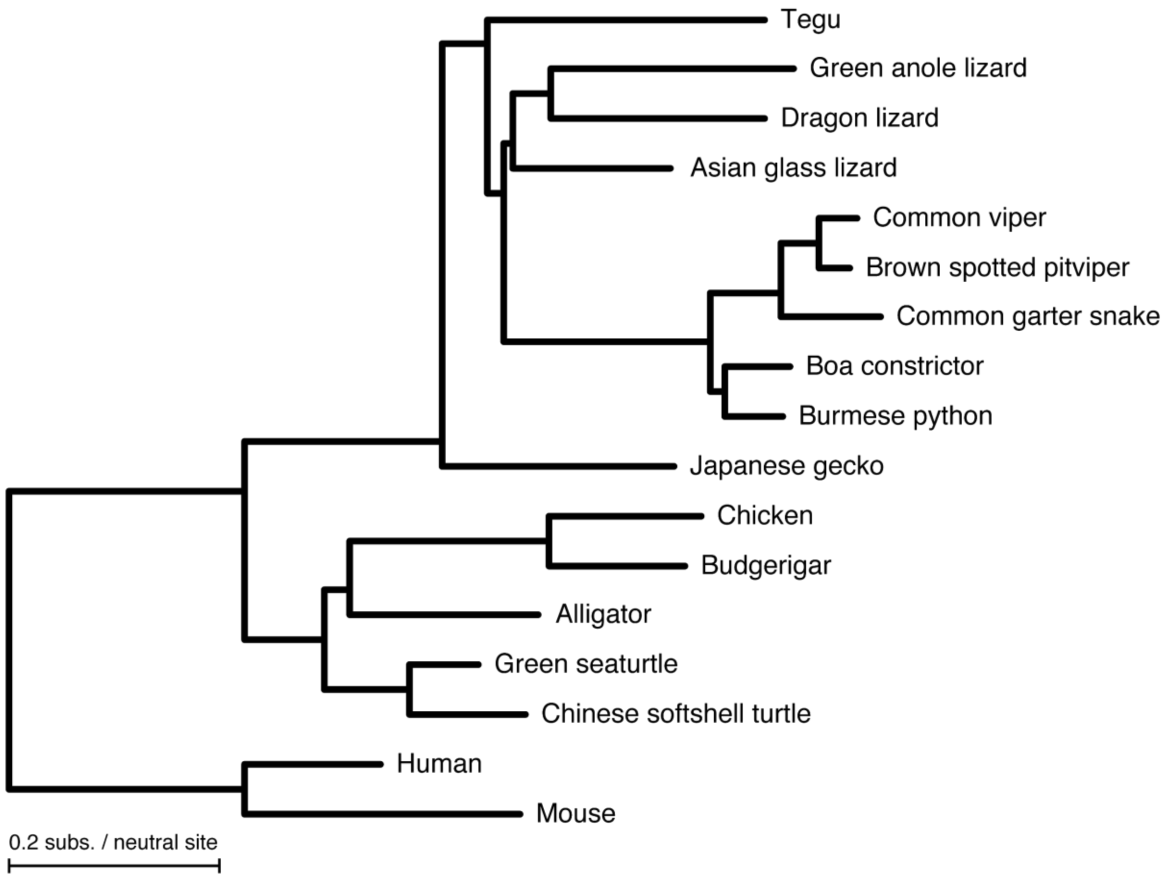

Figure 6: Phylogenetic tree of the amniote species included in our multiple genome alignment. The topology of the tree is based on references [66] and [68]. Branch lengths represent the number of substitutions per neutral site, as estimated from four-fold degenerated codon positions.

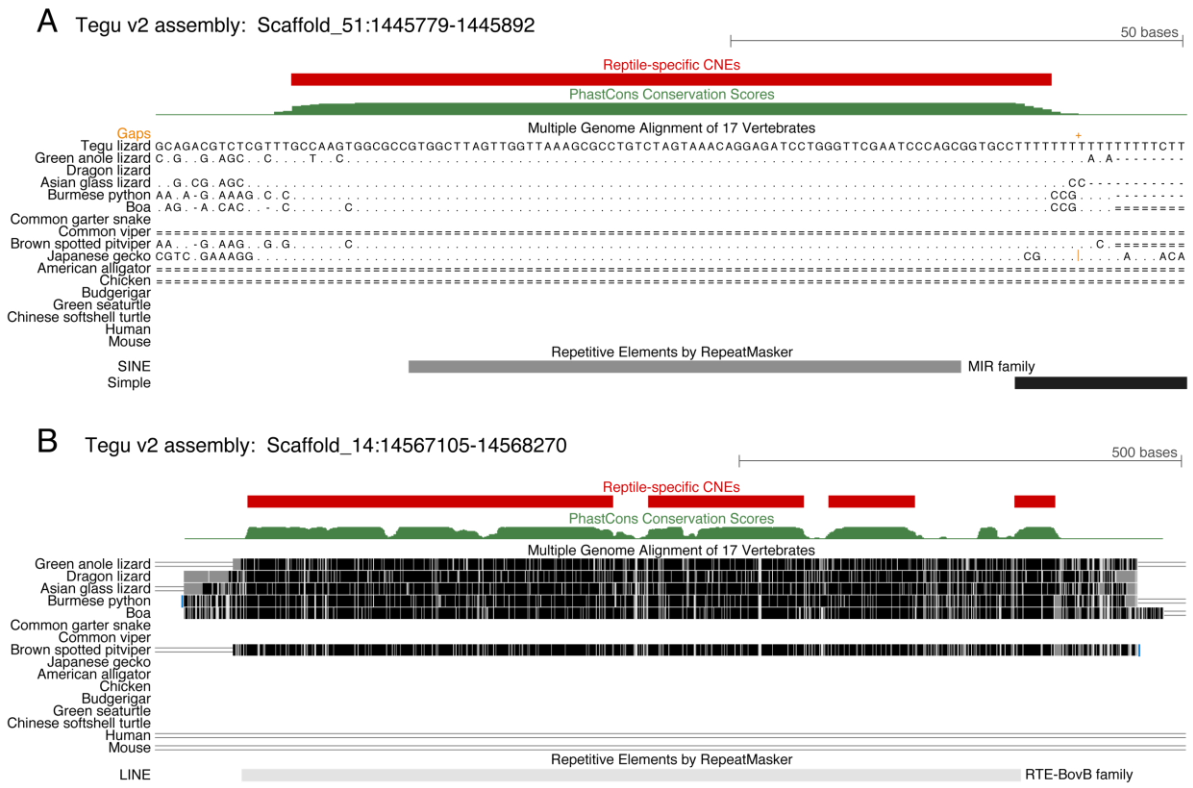

Figure 7: Transposon-derived conserved non-exonic (CNE) squamate-specific elements.

(A) A squamate-specific CNE likely originated from the insertion of a Short Interspersed Nuclear Element (SINE) belonging to the MIR family. The multiple genome alignment shows that this CNE is highly-conserved among squamates, but does not align to non-reptile species.

(B) Several squamate-specific CNEs likely originated from the insertion of a LINE of the RTE-BovB family. This insertion likely happened after the split from the lineage leading to geckos as no sequence aligns to the gecko genome.

Figure 1

Click here to access/download;Figure;fig1.

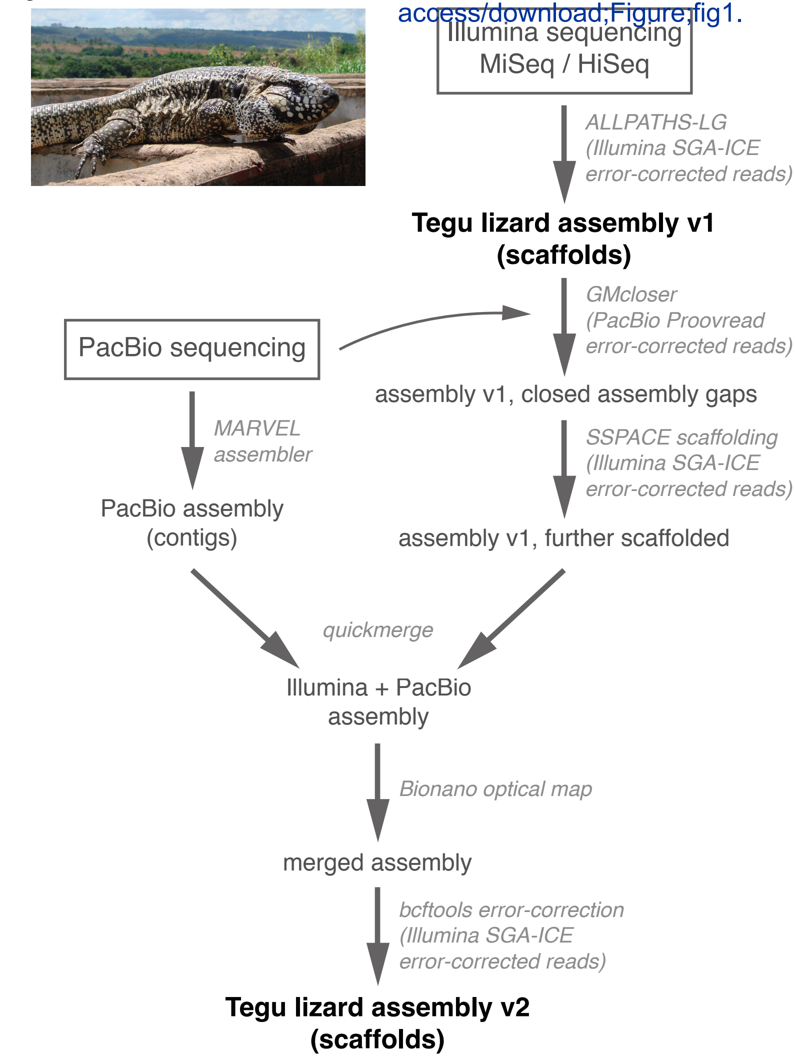

Figure 2

Click here to access/download;Figure;fig2.pdf

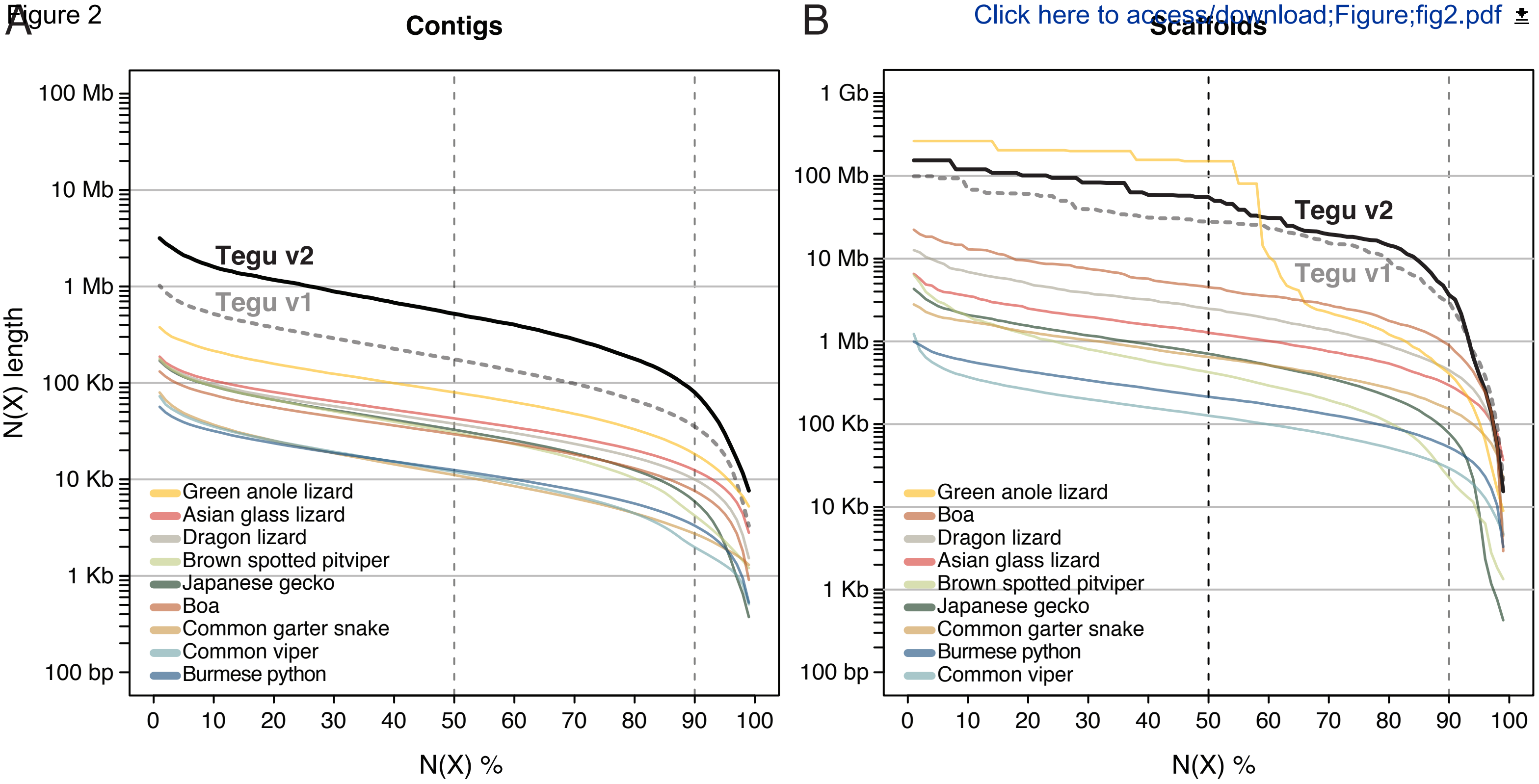

Figure 3

Click here to access/download;Figure;fig3.pdf

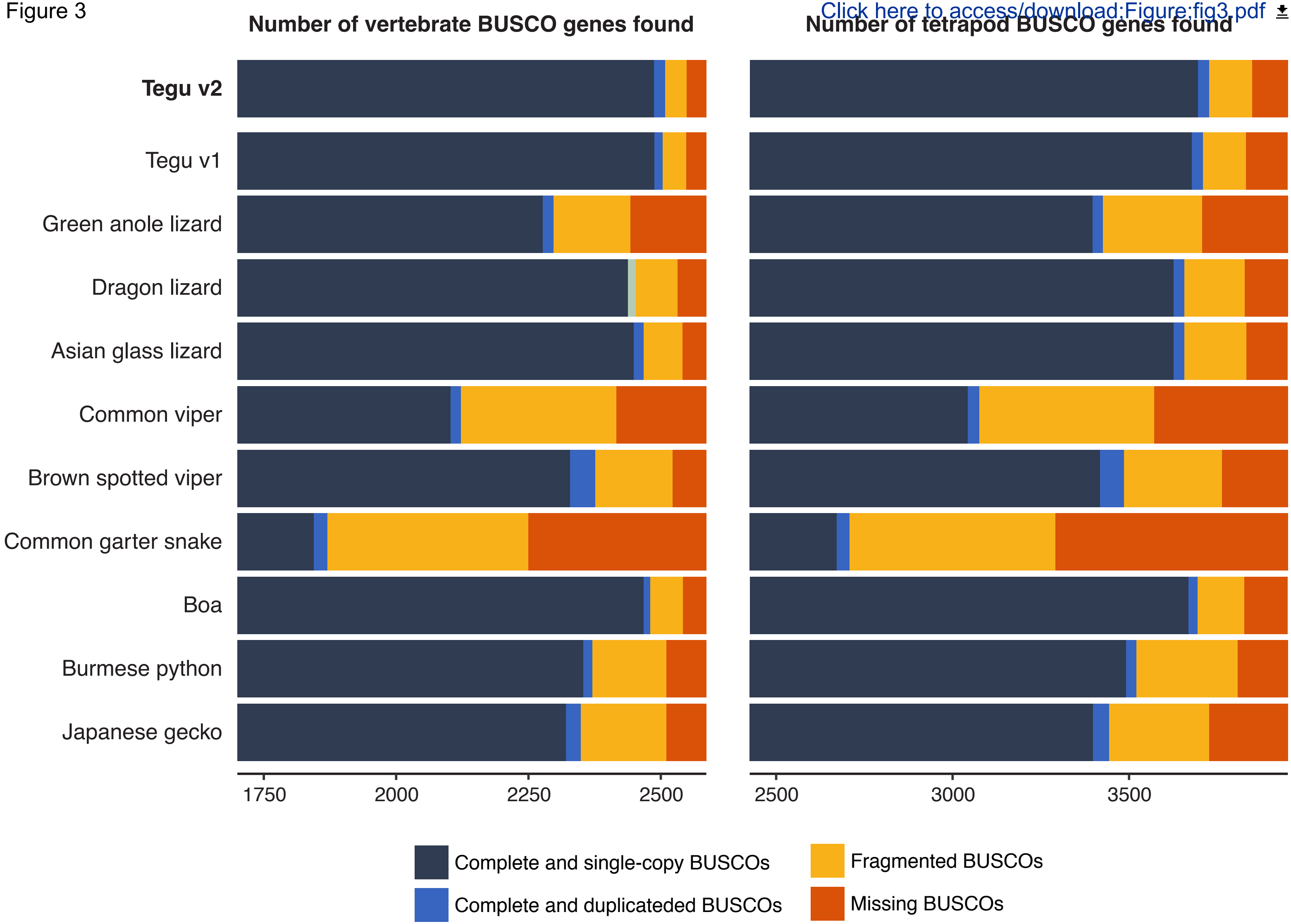

Figure 4

Click here to access/download;Figure;fig4.pdf

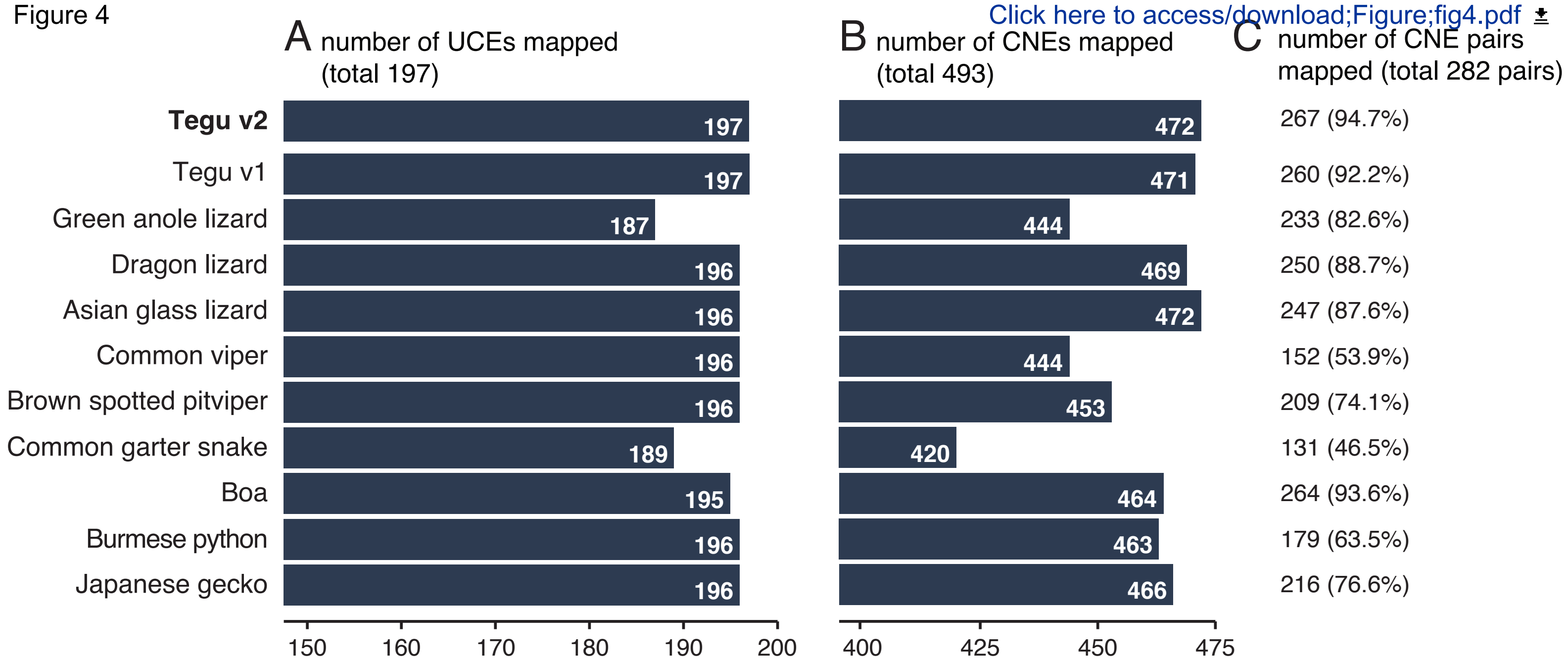

Figure 5

Click here to access/download;Figure;fig5.pdf

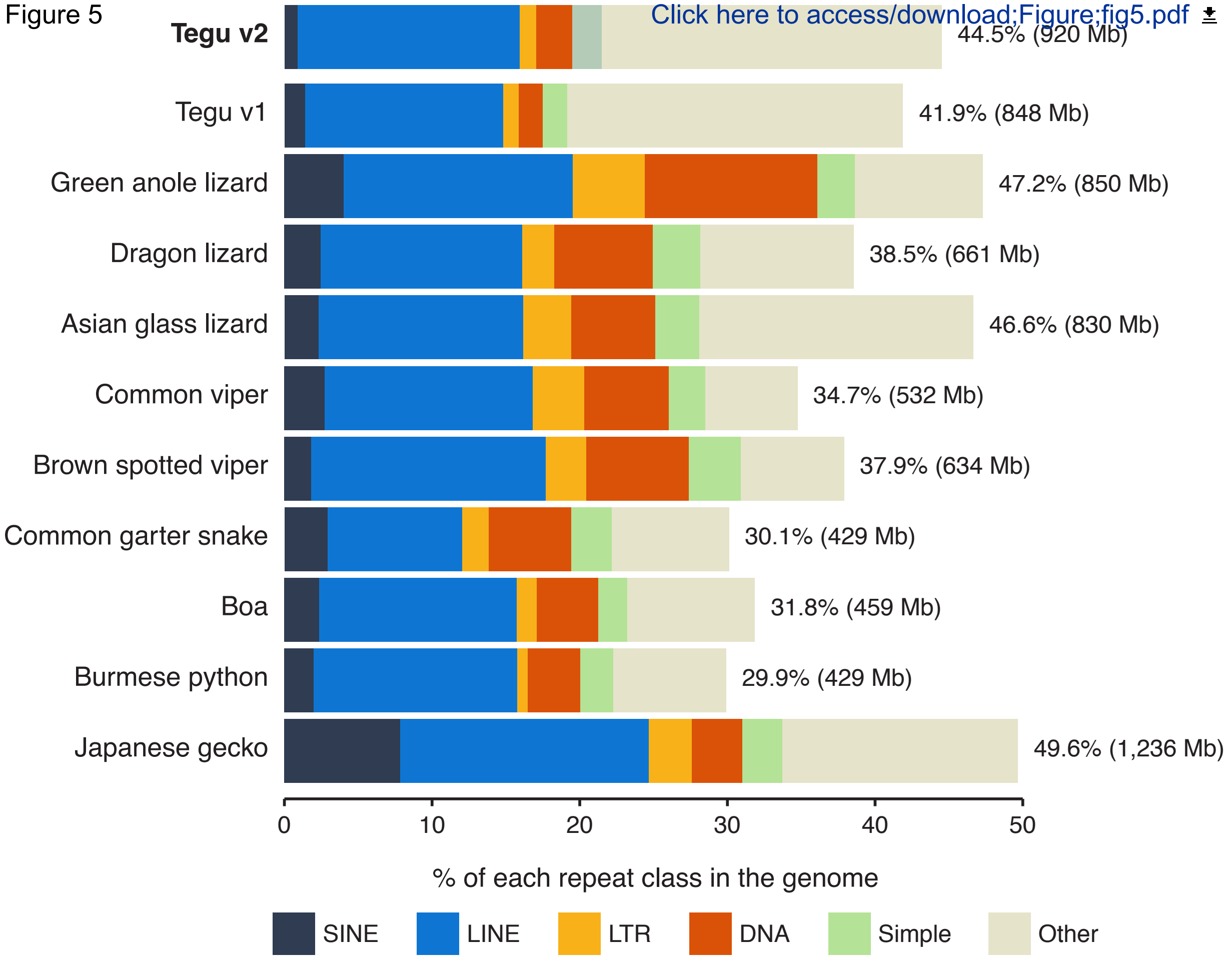

Figure 6

Click here to access/download;Figure;fig6.pdf

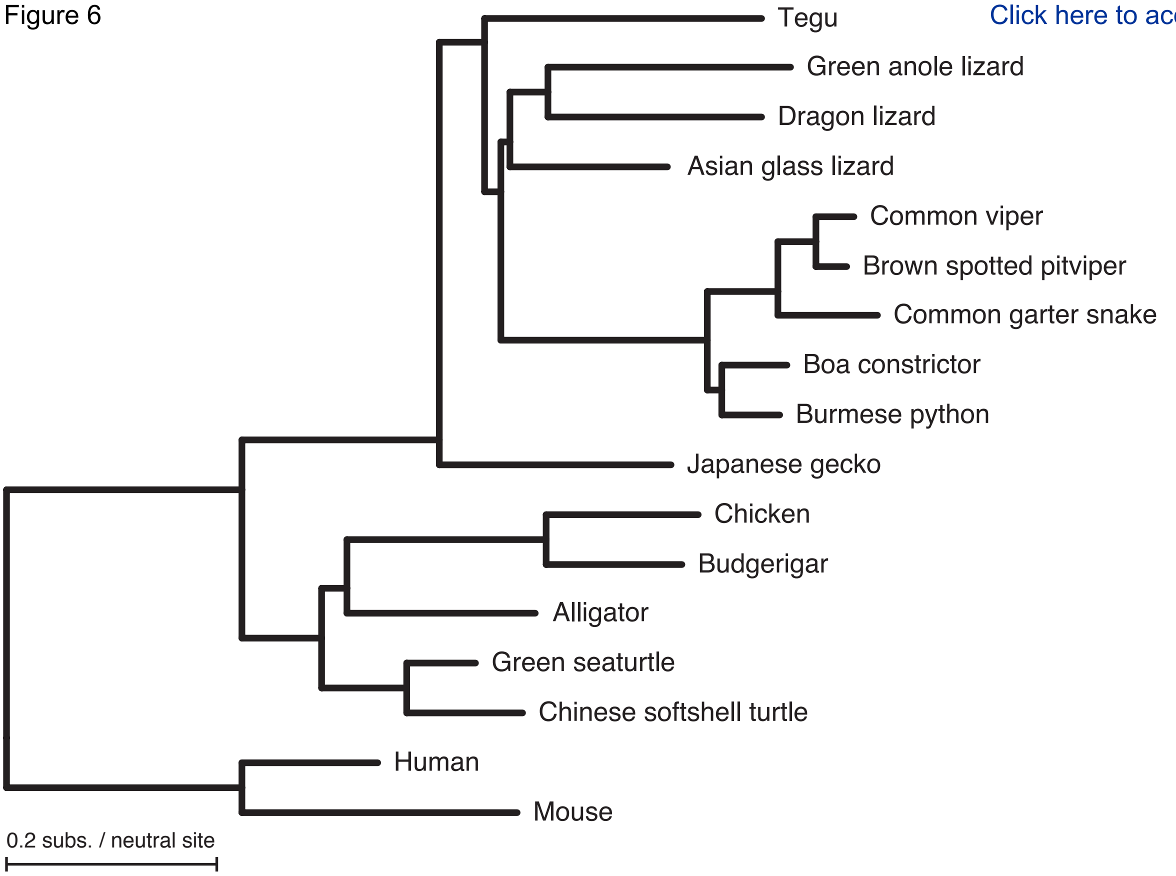

Figure 7

Click here to access/download;Figure;fig7.pdf

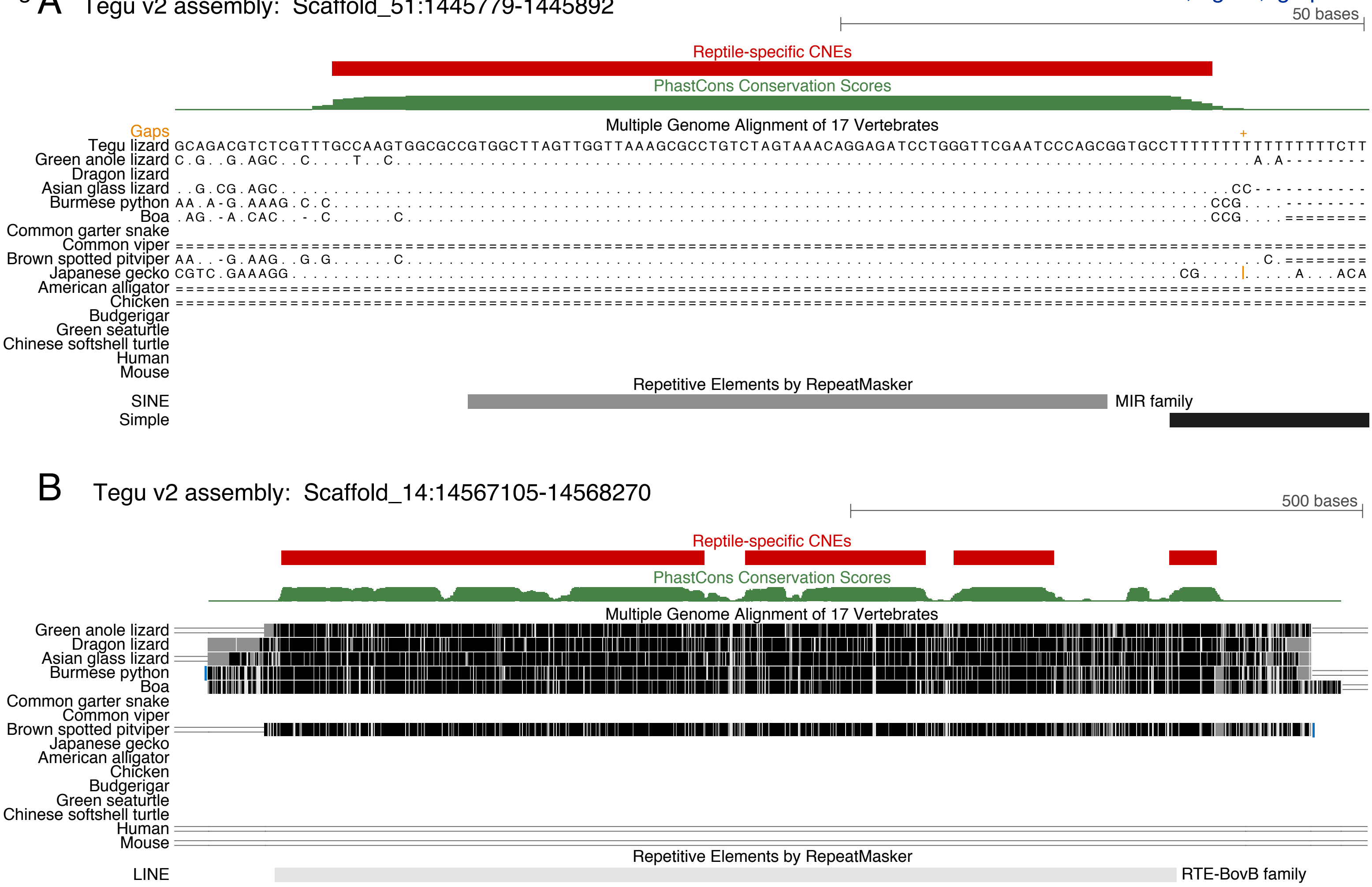

Supplementary Tables

Click here to access/download
Supplementary Material
SupplementaryTables.xlsx

Manuscript
Click here to access/download;Personal Cover;Responses.docx

Point-by-point response

Reviewer #1:

A well-assembled genome presented in a clear manuscript which provides important insights in conserved elements. It will greatly facilitate reptilian genome studies and comparative analyses.

We thank the reviewer for these kind words and helpful comments.

I only have some minor comments to improve the manuscript:

- line 63: I would replace 'non-avian reptiles' by 'Squamates' otherwise turtles and crocodiles should be indicated

Thank you for noticing that. We have now replaced ‘non-avian reptiles’ by 'Squamates' in the text.

- line 91: include reference to v1

Done.

- line 114: Independently

Corrected.

- line 143: I would move the section 'comparing assembly completeness' after the 'generating a resource for comparative reptile genomics' as it uses the multiple genome analyses

The purpose of the “Comparing assembly completeness” section is to evaluate the quality of the tegu lizard assembly, which belongs to the previous section “Comparing contiguity to other reptile assemblies”. To assess completeness and compare it between previously published reptile genomes, we made use of UCE and CNE datasets from Bejerano et al. 2004 and Hiller et al. 2013. Importantly, these datasets were defined based on conservation between vertebrate species (and not only reptiles). Therefore, these UCE/CNE data sets are independent from the reptile-focused multiple genome alignment that we produced and described in the resource section, and this has now been clarified in the text (please see the next point below). Thus, we would prefer to keep the current order of the paragraphs.

- line 158: Please add a short sentence here on how the CNEs were defined

We revised the sentence to include a CNE definition:
Pg.6, line 156, results: “In addition, we selected a larger set of 493 vertebrate-conserved non-exonic elements (CNEs; Supplementary Table 5), defined as regions longer than 300 bp that are conserved among mammals, teleost fish, shark and lamprey {Hiller, 2013 #23}, and counted the number of elements that aligned to the genome of each species with at least 80% coverage and 60% identity.”

- supplementary material: the 'tree.mod' file appears twice in the README

Thank you for noticing that, we removed the duplicated entry.

And a final suggestion to the authors: setting up a browser for the multiple genome assembly would prove particularly helpful.

We now provide a UCSC assembly hub that allows everybody to load and visualize the new tegu lizard genome and its annotations in the UCSC genome browser. We hope this will facilitate further analysis of the genome and using it for comparative genomics. This has been added to the text:

Pg.8, line 212, results: “The multiple alignment, the conserved regions, and the tegu gene annotation are available at https://bds.mpi-cbg.de/hillerlab/TeguGenomeData/ and can be loaded as an assembly hub into the UCSC genome browser [41] with the hub URL https://bds.mpi-cbg.de/hillerlab/TeguGenomeData/assemblyHub/hub.txt.”

----

1) Are the methods appropriate to the aims of the study, are they well described, and are necessary controls included? YES

2) Are the conclusions adequately supported by the data shown? YES

3) Please indicate the quality of language in the manuscript. Does it require a heavy editing for language and clarity? NO

4) Are you able to assess all statistics in the manuscript, including the appropriateness of statistical tests used? NA

Reviewer #2:

In this manuscript, the authors present an impressively well-assembled genome of the Tegu lizard that employs an interesting mixture of sequencing and scaffolding technologies. It is a very appropriate manuscript for publication in Gigasciences. From what I can tell, their genome assembly, annotation, and analyses are conducted in a robust fashion and all methodology seems quite sound and reasonable. There are, however, some important improvements and edits that should be made to the manuscript text itself before it would be ready for publication, and I outline these below.

We thank the reviewer for these kind words and helpful comments.

Suggested edits and additions:

I would like the authors to add a k-mer based estimate of genome size (using their illumina reads). Given the broad arguments in this paper that this genome assembly is highly complete and contiguous, evidence that the k-mer based estimate of genome size is fairly similar to the scaffolded genome size (for the v2 genome) would be important to demonstrate (and should be incorporated early in the results).

Thank you for the suggestion. We have used genomescope (Vurture et al. 2017, https://github.com/schatzlab/genomescope) to estimate the size of the tegu genome based on the Illumina sequencing reads. Using the default k-mer size of 21 bp, the minimum-maximum size estimate is 1,904,897,288 - 1,905,177,427 bp. This is a bit shorter than our final assembly size of 2.068 Gb and consistent with our relatively high assembly completeness estimates. We have added this information in the results and methods sections:
Pg.5, line 122, results: “The final v2 assembly of the tegu lizard genome has a size of 2.068 Gb, which is close to the 1.904 - 1.905 Gb size estimated by k-mer analysis of Illumina reads.”
Pg. 15, line 411, methods: ”K-mer size estimation. We used genomescope [51] to obtain a k-mer based estimate of the size of the tegu genome. We used the Illumina sequencing reads and the default k-mer size of 21 bp, and obtained a minimum-to-maximum estimate of 1.904 to 1.905 Gb.

In some figures (e.g., Figure 3) the color schemes seem overly dark and are very difficult to read due to the low contrast among categories of data shown. These figure color schemes should be revised to use color palates that are more easy to read/interpret.

We followed the suggestion and changed the color palette of Figures 3 and 6. In Figure 3, the new colors are on purpose more similar between the complete (single-copy or duplicated) BUSCO genes and between the fragmented/missing BUSCO genes.

I would also like the authors to "soften" their conclusions about the links between CNEs and transposons (starting around Line 218, and below). The authors compared the overlap between identified conserved non-coding elements (CNEs) and their repeat annotation, and find that some of these annotations (n=146) overlap. My concern (e.g., Line 219) is that the authors conclude that the CNEs "originate from transposons". While interesting, the only evidence the authors have is that they overlap, suggesting that some CNE's "may originate" from transposons. Because there is no functional work, I would prefer if the authors used more tentative language throughout this section to state more clearly what the facts are (i.e., some overlap) and could possibly indicate that transposons may have given rise to CNEs. The heart of my concern is that both transposons and CNEs are thought to include regulatory sequences and the annotation of a sequence as a transposon not necessarily mean that it was actually derived from a transposon, but instead shares high sequence homology to a transposon. Also, given the frequency of transposon DNA in the genome, transposons often directly flank CNEs, further increasing chances that the annotation of a CNE also as a transposon can be spuriously interpreted as a transposon sequencing giving rise to a CNE. Therefore rather than stating "LINEs contribute the most CNEs" (e.g., Line 219-220), it would be better to say that "LINEs most frequently overlapped with CNE annotations"… Also, on the next line, this should be changed to "Overall, these data suggest that transposons (and LINEs in particular) may have given rise to a substantial number of reptile-specific CNEs"…

We agree and have changed the text as suggested:

Pg 9, line 223, results: "By intersecting squamate-specific CNEs with transposons, we found that only 146 of the 47,931 CNEs (0.3%) overlap transposons (Figure 7). These 146 CNEs sum up to 19.8 Kb, 86% of which overlap LINEs, consistent with this transposon class being the most abundant one in the assembly (Figure 5)."

We changed the last sentence to:
"Overall, transposons may have given rise to only 0.6% (19.8 kb of 3.3 Mb) of the bases in squamate-specific CNEs."

Overall, the Discussion is poorly written and lacks any actual discussion, but instead consists of multiple paragraphs that simply re-state the results. I would ask the authors to include some new more synthetic discussion points here. Examples might include: (i) how their findings of many reptile-specific CNEs, and their possible links to transposons, open new questions about the roles of transposons that would be interesting for future studies, (ii) the relevance of their findings that repeat content may be substantially under-represented in less contiguous genome assemblies, (iii) some discussion of the variation in gene annotations across squamate genomes, and the potential that some are either massively over-counted (due to fragmentary assemblies) or undercounted due to the extreme fragmentary nature of others, based on the finding of about 14,000 genes in this highly contiguous genome and based on their Busco analyses, (iv) some broad discussion of the importance of more highly contiguous and complete assemblies for addressing sets of questions in squamates that poor genomes cannot adequately address.

Thank you for these suggestions. We have now added a discussion for points i, ii and iv:
(i) "Interestingly, even though the repeat content of reptile genomes is fairly high, very few of the squamate-specific CNEs overlap transposable elements. This contrasts with previous observations in mammals, where 16% of the placental mammal-specific CNEs overlap transposons [42]. Squamates are an older lineage compared to mammals (~200 vs. ~100 Mya), which makes the identification of ancient squamate-specific repeats more challenging. Nevertheless, the CNE-transposon overlap is more than 50-fold lower in squamates, which highlights a difference in how functional lineage-specific non-exonic elements evolved in these two clades."
(ii) "Furthermore, the new v2 assembly contains several additional megabases of repetitive sequences that were not present in the previous Illumina-based v1 assembly. This illustrates the ability of long PacBio reads to add repetitive sequence to a short-read assembly and thus increase the completeness in the repeat content of an assembly."
(iv) "Together, this allows evaluating assembly completeness and contiguity in the non-exonic regions of an assembly. Since *cis*-regulatory elements typically reside in non-exonic regions, it is important to have a high completeness and contiguity in the intergenic portion of the genome, especially when applying high-throughput functional genomics methods, such as ChIP–seq or ATAC-seq, to discover regulatory elements."

With respect to point (iii), we would like to point out that the number of annotated genes for the tegu lizard is similar to the gene number of other lizards (19,400 – 25,385). Furthermore, we improved the gene annotation, giving gene predictions based on the annotation of the v1 assembly as additional evidence set for MAKER. This improved gene set now contains 22,413 genes, and has a BUSCO completeness score of 94.1%. We now add:
Pg.8, line 198, results:
"Since CESAR was run on a genome alignment that makes extensive use of conserved alignment order, these 16,995 tegu loci likely contain orthologs of human genes. Fourth, we used BRAKER [39] to obtain gene predictions based on mapped RNAseq data and the previous gene set from v1 assembly, resulting in 75,444 predictions after removing short, overlapping genes. The final gene set produced by MAKER contains 22,413 genes (BUSCO completeness score of 94.1%), which is within the range of number of genes annotated in other lizards [2, 4, 12, 13, 16]"

Also, the author's current comment in the first discussion paragraph about the repeat content of anolis is higher in their study than a single previous study is inappropriate because the repeat content of Anolis has been re-estimated in a handful of studies since the publication of the Anolis genome (and these studies are not cited).

We removed this sentence from the discussion.

Text Copy Edits and Suggestions:

There are many grammatical errors in the paper, and I have tried to identify many of these below, although I would strong encourage the authors to have a very careful additional round of review to catch ones I may have missed.

Thank you for noticing these mistakes. We corrected them all with the exception of the Line 30. We also carefully read the entire manuscript again.

Line 29 - change "with a great…" to "with great…"
Line 30 - delete "the" before "vertebrate species"
Since we are specifically referring to only those vertebrate species that have sequenced genomes, we feel that the 'the' should remain.

Line 40 - replace "largest" with "most abundant"
Line 41 - change "transcriptomics" to transcriptomic"
Line 63 - there are multiple issues with this sentence that could be fixed by rewriting to: Squamate reptiles (i.e., lizards and snakes) are heavily under-represented…"

Paragraph starting on line 62 - this paragraph is mostly a long list of the genomes that have been sequenced for squamates. It seems like this paragraph would be better served as a table, and more rapidly referred to in the text. Also, the point at the end that I think is meant to point out the lack of many lizard species is not made clearly and should be reworded.

We agree that the sentence listing all available genomes was too long. We have therefore now drastically shortened it to:
Pg.3, line 70, introduction: "Right now, nine snake species (*Boa constrictor*, Burmese python, two rattlesnakes, king cobra, garter snake, corn snake, and two vipers) and six lizards (green anole, two geckos, Asian glass lizard, dragon lizard, Chinese crocodile lizard) have assembled genomes [3-16]."
We would prefer to keep this shorter list of sequenced squamate species in the main text to properly acknowledge previous work of the reptile genomics community and to cite the respective publications.

And we agree that the point of the last sentence in this paragraph was not clear. Therefore, we have removed the last sentence and rewrote the start of the paragraph:
Pg.3., line 62, introduction: "Squamate reptiles comprise a species-rich group of approximately 6,500 lizards, 3,700 snakes, and 200 amphisbaenian species [1]. However, this group is heavily under-represented among the vertebrate species with sequenced genomes, especially considering the great morphological, behavioural, and life history diversity in this group"

Lines 87-88 - delete this last sentence because it isn't particularly important and because it doesn't totally make sense to me (i.e., why are non-native species considered endangered?)…

Non-native species can have conservation value and can be environmentally relevant for the preservation of native species, as argued in https://www.nature.com/articles/474153a and https://onlinelibrary.wiley.com/doi/abs/10.1111/j.1523-1739.2010.01646.x. Nevertheless, we agree that the part "native and non-native" is not necessary and removed it from the sentence.

Line 93 - change "completeness in genes" to "completeness of genes"
Done
Line 95 - change "annotation" to "annotations"
Done
Line 163 - change "contained less" to "contained fewer"
Done

Lines 187 - rewrite to avoid using "repeat modeled and repeat masked" as verbs. For example, rewrite to: "We also used repeat modeler and repeat masker to identify repeats in other reptile genomes…"

> We changed this sentence to:
> “We also modelled and masked repeats in the other reptile genomes analyzed in this study and found a similar repeat content…”

Reviewer #3:
This manuscript by Roscito and his colleagues reported an update genome assembly of the tegu lizard Salvator merianae by hybrid assembly with data from Illumina, PacBio and Bionano. By doing so, the authors improved the genome assembly by increasing the scaffold N50 from 28Mb to55Mb and contig N50 from 175kb to 521kb, making the tegu lizard genome as the highest assembly contiguity among published reptiles genomes. Testing the BUSCO analysis for the vertebrate core gene conserved non-coding elements (CNEs) analysis showed the assembly has the highest completeness compared to other reptile genomes according to the authors' criteria. The authors also generated the multiple genome alignments of 17 species and identified conserved regions.

We thank the reviewer for these kind words and helpful comments.

General comments:
Data quality: The long reads generated by Pacbio or nanopore have notorious problem for the high sequencing error rates. Many efforts have been done and published before to polish the raw sequencing reads and improve the overall quality. Still, the safest way of using pacbio long reads technology is to produce high depth of data and polish the raw reads by self-correction for systemic error. Although the short reads correction could correct the mismatch, the method has limit capacity in dealing with the indel errors for pacbio reads. The authors only produce <30x pacbio data, restricted them to use the self-correction method for polishing. The quality of the hybrid genome assembly might be a concern.

We agree that <30X PacBio coverage is not sufficient for self-correction. However, we would like to point out that we used Proovread and our 2x300 bp Illumina MiSeq data (41X coverage) to correct errors in the PacBio reads before using these reads to improve the assembly. Proovread is one of the most accurate methods and we specifically used it because it does not break the longer-than-usual Illumina MiSeq reads into smaller k-mers for a De-Bruijn-graph based error correction. While correcting the PacBio reads with Proovread was computationally very expensive, we found that this method and our data is able to correct indel errors in PacBio reads. Furthermore, as an additional measure to correct remaining base errors, we polished the final assembly using FreeBayes and bcftools with our Illumina data, as it is now likely becoming the standard in the Vertebrate Genome Project.

We would also like to mention that despite merging our previous Illumina-only assembly with a new PacBio assembly and using PacBio reads for gap closing, a large portion of the bases were already contained in our previous Illumina-only assembly and most of these bases remain unchanged (please see below).

Finally, it is expected that uncorrected base errors (in particular uncorrected indels) would create frameshifts in genes, which BUSCO analyses would detect as fragmented genes. As shown in Supplementary Table 3, BUSCO finds that our v2 assembly has only 1.6% fragmented genes, which is lower than all other Illumina-only assemblies that we compared. BUSCO also shows that our v2 assembly has 97% complete genes, which is higher than other assemblies. Together, this provides evidence that the base quality of our assembly is at least not worse than for other available squamate assemblies.

Since the authors also produced the assembly with short reads, I would recommend the authors to perform the comparison for the two genome assembly versions and produce a summary for the difference (indels, alignment quality etc.).

Thank you for this suggestion. To compare both assembly versions, we aligned the v1 and v2 assemblies to each other with lastz and determined the number of identical bases and the number of substitutions and indels. This analysis showed that both assemblies are very similar to each other, with 99.83% of the bases in the v1 assembly being unchanged in v2. The percentage of bases corresponding to substitutions, insertions, and deletions is low: 0.12%, 0.04%, and 0.0028% respectively, the majority of which correspond to polymorphisms.

We added this information as a new paragraph:
Pg.16, line 416: “Comparison of the tegu v1 and v2 assemblies. To analyze the sequence similarity between both assemblies, we aligned the v2 assembly to the v1 assembly as described previously

[38], but with lastz [52] parameters *'--gappedthresh=8000 --hspthresh=4000'*. Then we determined the sequence identity in all aligning regions (note that no comparison can be made in assembly gap regions that were closed in v2). This showed that 99.83% of the bases in the v1 assembly are unchanged in v2 and that both assemblies differ in 0.12% substitutions, 0.04% insertions and 0.0028% deletions. Inspecting the differences and the aligned Illumina reads showed that almost all differences between both assemblies correspond to polymorphisms, where reads support both variants and the assembly polishing step changed identity of the variant."

It would be even better if the authors could calculate the quality value for each single base of the new assembly, by using the short reads mapping results.

We used bcftools mpileup and bcftools call and our Illumina sequencing data to obtain a per-base quality score for the tegu v2 assembly. For each base in the assembly, bcftools determines a score as well as the potential variant of a position, using information contained in the bam file of the mapped sequencing reads.

Overall, the vast majority of the bases (99.8%) in the tegu v2 assembly have a Phred quality score ≥40, which corresponds to a base accuracy of 99.99%. The per-base quality scores are loaded in the UCSC assembly hub that we set up (https://bds.mpi-cbg.de/hillerlab/TeguGenomeData/assemblyHub/hub.txt; please see the comment of reviewer 1 above).

We added this information to the methods:
Pg.15, line 405, methods: "Obtaining per-base quality values. We used bcftools [50] with the Illumina sequencing reads (parameters '*bcftools mpileup -A* | *bcftools call -c*') to obtain a quality value for each base of the assembly where a read is mapped to. Overall, 99.8% of the bases in the tegu v2 assembly have a Phred quality score greater than 40, which corresponds to a base accuracy of 99.99%."

I could not find any assessment for the quality of the pacbio raw reads. This is also an important information for others who might be interested to use the raw reads and probably to further improve the genome by producing more pacbio reads.

We apologize for not including it before. We have now created a new supplementary table (Supplementary Table 1) describing the length statistics of the PacBio reads in detail.

Furthermore, since the PacBio Q values are not very informative in our experience, we have computed intrinsic quality values of the PacBio reads, as described in the dazzlerblog (https://dazzlerblog.wordpress.com/2015/11/06/intrinsic-quality-values/). Briefly, for each read, every other overlapping read is determined. Afterwards, for all 100 bp segments of the read, we compute the average similarity to the 50% best-aligning reads. These intrinsic quality values are entirely derived from the data itself and reflect the error rate in the selected read.

We obtained the following histogram of intrinsic quality values. The histogram shows that most segments are between quality values of 15 - 25, which is expected given that a random error rate of ~12-15% will yield pairwise read differences of 24-30%. The tail of the distribution also shows that there are reads with a very poor quality. This is also consistent with the read coverage dropping to <25X after sequencing artefact correction in MARVEL's patch phase. However, these reads would not be used in the MARVEL assembly and they would not align to Illumina contig end, and thus would not contribute to gap closing. Overall, the quality values of the PacBio reads are in the expected range.

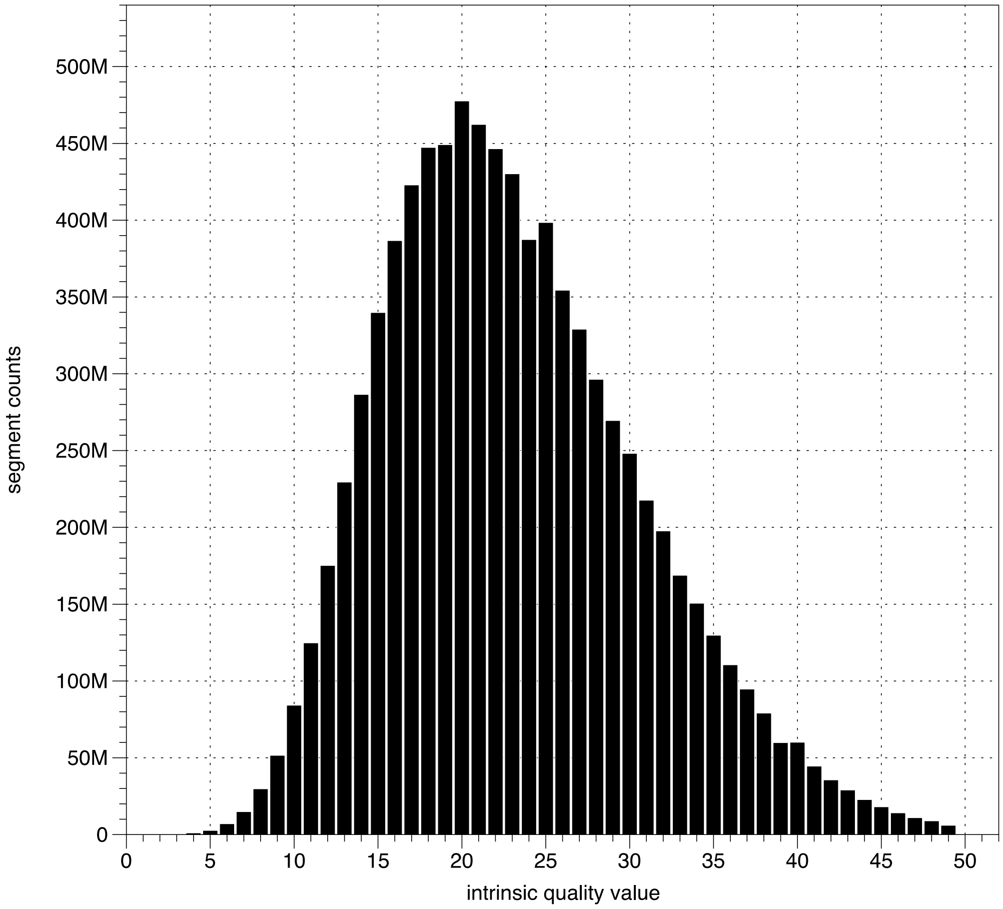

Data access: all data should be uploaded online or the GigaDB. I only found the final hybrid assembly data in the website they offer. The authors should upload all the raw data and the v1 genome assembly with short reads.

> We apologize that we uploaded all data to NCBI after submitting this manuscript. Now, all raw data produced in this study has been uploaded to SRA under the project accession number PRJNA473319 (https://www.ncbi.nlm.nih.gov/bioproject/473319). All Illumina and PacBio data are already available there. Unfortunately, both genome assemblies (v1 and v2) that were submitted weeks ago to NCBI are still being processed by NCBI. We have repeatedly emailed the NCBI staff and they now confirmed that both assemblies will be available soon under the accession number PRJNA473319. If this does not happen, we would be happy to also upload the assemblies to GigaDB. In any case, we guarantee that all data is freely and publicly available (currently at https://bds.mpi-cbg.de/hillerlab/TeguGenomeData/).
>
> This has been added to the Data availability statement:
> "All raw sequencing data and genome assemblies are available at NCBI under the project accession number PRJNA473319. All other data, including annotated genes, the multiple genome alignment, and conserved element datasets are available at https://bds.mpi-cbg.de/hillerlab/TeguGenomeData/. The genome and its annotations can also be loaded into the UCSC genome browser as an assembly hub (URL https://bds.mpi-cbg.de/hillerlab/TeguGenomeData/assemblyHub/hub.txt)"

Minor comments:

Abstract, I would suggest the authors to add a summary table for the data they have produced main text

so the readers could easily capture what data has been used in the new genome assembly. And it would be even better if the authors could indicate the coverage for each dataset in abstract.

As suggested, we have added the coverages to the abstract:
"We combined 74X Illumina short read, 29.8X PacBio long read and …"

We revised the first sentence in the Results to specify the exact coverage and read lengths of our Illumina MiSeq and HiSeq data:
Pg.4, line 98, results:
"The first version of the tegu genome (v1) was assembled with ALLPATHS-LG [22] using high-coverage Illumina sequencing data (41X 2x300 bp MiSeq reads and 33X 2x150 bp HiSeq reads; Supplementary Table 1), resulting in a 2.026 Gb assembly with a scaffold N50 of 28.146 Mb (5,988 scaffolds) [21]."

The new Supplementary Table 1 also provides the coverage of the PacBio data together with statistics about read lengths.

Line 106, the scaffold N50 should be 28.146Mb, instead of 28,146 Mb. Contig N50 should be 175.755kb.

Thank you for noticing these mistakes. They have been corrected.

Line 193, "protein sequences from closely-related species", how many closely-related species used, which species used? Please clarify.

We used high-quality protein data with experimental support for sauropsids species, downloaded from UNIPROT. We have now added information about the exact species and how many proteins were available for each in a new Supplementary Table 7. The text in the Results and Methods has been updated to specify the details:
Pg7, line 190, results: "To annotate genes in the tegu lizard genome we used MAKER [36] with four types of input data: transcriptome data, protein sequences from 33 sauropsid species (Supplementary Table 7), human genes mapped to the tegu lizard genome, and gene predictions based on the gene annotation of the v1 assembly"
Pg18, line 477, methods: "Second, we downloaded protein sequences available on UNIPROT (data accessed in March/April.2018; 20 lizard species, 9 snake species, chicken, softshell turtle, and two alligator species; Supplementary Table 7). We only kept those proteins with strong experimental evidence (sequences annotated with PE=1 or PE=2), resulting in a total of 3,739 protein sequences."

Line 201, The number of genes produced by MAKER (19,101) < number of transcripts (19,128). Do you set the parameter alt_splice=1, which produce alternative splicing for each gene. I could not find it in the MAKER parameters. If so, why there are only 27 alternative splicing transcripts?

No, we used MAKER with alt_splice=0. This parameter has now been added to the Methods section. The 27 isoforms are contained in the annotation because of Augustus, which was running as part of MAKER and predicts alternative transcripts for a few genes. This seems to be an intrinsic feature of Augustus, which MAKER can't override.

We would like to mention that we improved the gene annotation for this assembly during the revision of this manuscript by providing an additional evidence set for MAKER (see pg.18, methods). Our new and final gene set contains now 22,413 genes and 22,417 transcripts (the small difference being, again, because of Augustus). Compared to our previous annotation, the new gene set has a higher BUSCO score of 94.1% complete genes.

Line 380, Here you used FreeBayes to detect variants. How many homozygous variations detected? How many base errors corrected? Can provide a summary here.

The majority of the 6.3 million corrections performed by FreeBayes, based on the Illumina data, corresponds to heterozygous positions (82.8%). 17.2% correspond to erroneous base calls, i.e., the reference base is incorrect. A summary of the number of corrected errors is now included in the Methods.

Pg.15, line 399, methods: “To correct remaining base errors, we used the variant detector FreeBayes [48] and bcftools consensus [49] with a score cutoff of 1, to detect and correct erroneous or polymorphic positions in the assembly. Of a total of 6,322,937 assembly positions where the base identity was changed (0.3% of the genome), 82.8% correspond to heterozygous positions and 17.2% correspond to erroneous base calls in the original assembly.”

Line 394 "collapsing redundant transcripts and clustering overlapping transcripts." How do you collapse and cluster the transcripts? Do you choose the transcript with longest ORF as representative from the same gene locus? I would expect the number of transcripts after collapsing and clustering to be falsely high.

We apologize that it was not clear from the respective sentence how this step is performed. We did not clustered overlapping transcripts ourselves, but used the PASA pipeline for that. Specifically, PASA only collapses transcripts that align to the same locus, have significant overlap in their exons and map to the same strand. That means, antisense transcripts are not clustered together. We clarified this in the Methods by writing:

Pg.16, line 435: “PASA also removed low-quality alignments (alignment identity less than 95% and minimum of 75% aligned) and combined both trinity and cufflinks transcripts by collapsing redundant transcripts and clustering transcripts that have overlapping exons on the same strand.”

Line 445, I suggest change "reading frame" to " open reading frame"

Done

Figure 1, I suggest change "BCF error-correction" to "BCFtools error-correction" to make it clear to understand.

The figure has been changed accordingly.

Figure 6, please provide citation the phylogenetic tree was taken from.

The tree topology was taken from a recent phylotranscriptomic study (Irisarri et al. Nat Ecol Evol. 2017) and from a detailed study of the squamate phylogeny (Pyron et al. BMC Evo Bio. 2013). This information is now added to the legend of Figure 6.

Other minor issue, the authors have used Tegu lizard genome as reference. However, the scientific interest of this species has not been well described in the introduction section. It is better to justify the argument to use this species as reference, as most other would prefer using other species like the green anole lizard as reference.

The reason for using the tegu lizard as the reference is that its assembly has a higher contiguity than that of the anole lizard. In particular, the tegu lizard contigs have N50 values 6.5-times larger than that of the anole (521 vs. 80 Kb), which implies much less assembly gaps. Genomic regions that are assembly gaps in the reference genome would not be visible in the genome alignment when visualized in a genome browser of the reference. Therefore, using anolis as the reference would result in many truly-aligning regions that would be missed. We clarified this in the Results section:

Pg.8, line 208, results: “Since the tegu lizard genome is currently the most contiguous assembly, we used it as the reference.”

Manuscript Click here to access/download;Personal Cover;CoverLetter.docx

Dr. Michael Hiller
Senior Research Group Leader
Max Planck Institute of Molecular Cell Biology and Genetics
Pfotenhauerstr. 108, Dresden, Germany
Email: hiller@mpi-cbg.de
Phone: +49 351 210-2781
https://www.mpi-cbg.de/hiller

Sep 26th, 2018

Dear Dr. Edmunds,

Thank you very much for considering a revised version of our manuscript "**The genome of the tegu lizard *Salvator merianae*: combining Illumina, PacBio, and optical mapping data to generate a highly contiguous assembly**".

We would like to thank all three reviewers for their constructive and insightful comments. We have now addressed all points raised and revised the manuscript by adding new analyses. In particular, we

- provide an assembly hub that allows browsing and analyzing the tegu genome and all the annotations that we generated (genes, repeats, CNEs, multiple genome alignment) in the UCSC genome browser,
- performed a k-mer analysis, which provided a genome size estimate that is close to our genome assembly size and confirms assembly completeness,
- performed a comparison of the v1 and v2 assembly, showing that 99.83% of the bases are unchanged and most of the differences are due to polymorphisms,
- generated assembly quality score tracks, showing that 99.8% of the bases have Phred scores ≥40 (99.99% accuracy),
- changed figure colors to improve readability,
- added additional discussion points to the Discussion section.

We have uploaded all assembly and sequencing read data to NCBI. All data except the genome assemblies is already publicly available under the BioProject PRJNA473319. Unfortunately, the NCBI staff has not yet released the v1 assembly (which is already approved), and has not yet processed our v2 assembly submission, despite submitting both assemblies weeks ago. We are working with the NCBI staff to accelerate this process as much as possible. In the meantime, both v1 and v2 assemblies are available at https://bds.mpi-cbg.de/hillerlab/TeguGenomeData/. In case

this process is further delayed, we can also upload them to GigaDB. Importantly, all data generated for this manuscript is freely and publicly available.

All text changes in the manuscript are highlighted in red font. Please find our point-by-point response to the comments raised by the reviewers uploaded as a separate Word document.

We hope that our revised manuscript is now acceptable for publication in *GigaScience*. We look forward to hearing from you.

Sincerely,

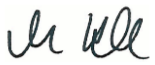

Michael Hiller
