# Supplementary material for: The genome of the tegu lizard Salvator merianae: combining Illumina, PacBio, and optical mapping data to generate a highly contiguous assembly
Source: Gigascience. 2018 Nov 27;7(12):giy141. doi: 10.1093/gigascience/giy141 (PMC6304105; doi:10.1093/gigascience/giy141)
Supplement: reviewer_2_report_(original_submission) -- Todd Castoe — 6/11/2018 Reviewed [file giy141_reviewer_2_report_(original_submission).pdf]

Reviewer Report

Title: **NanoAmpli-Seq: A workflow for amplicon sequencing for mixed microbial communities on the nanopore sequencing platform. The genome of the tegu lizard Salvator merianae: combining Illumina, PacBio, and optical mapping data to generate a highly contiguous assembly**

Version: **Original Submission** **Date:** 6/11/2018

Reviewer name: **Todd Castoe**

Reviewer Comments to Author:

In this manuscript, the authors present an impressively well-assembled genome of the Tegu lizard that employs an interesting mixture of sequencing and scaffolding technologies. It is a very appropriate manuscript for publication in Gigasciences. From what I can tell, their genome assembly, annotation, and analyses are conducted in a robust fashion and all methodology seems quite sound and reasonable. There are, however, some important improvements and edits that should be made to the manuscript text itself before it would be ready for publication, and I outline these below.

Suggested edits and additions:

I would like the authors to add a k-mer based estimate of genome size (using their illumina reads). Given the broad arguments in this paper that this genome assembly is highly complete and contiguous, evidence that the k-mer based estimate of genome size is fairly similar to the scaffolded genome size (for the v2 genome) would be important to demonstrate (and should be incorporated early in the results).

In some figures (e.g., Figure 3) the color schemes seem overly dark and are very difficult to read due to the low contrast among categories of data shown. These figure color schemes should be revised to use color palates that are more easy to read/interpret.

I would also like the authors to "soften" their conclusions about the links between CNEs and transposons (starting around Line 218, and below). The authors compared the overlap between identified conserved non-coding elements (CNEs) and their repeat annotation, and find that some of these annotations (n=146) overlap. My concern (e.g., Line 219) is that the authors conclude that the CNEs "originate from transposons". While interesting, the only evidence the authors have is that they overlap, suggesting that some CNE's "may originate" from transposons. Because there is no functional work, I would prefer if the authors used more tentative language throughout this section to state more clearly what the facts are (i.e., some overlap) and could possibly indicate that transposons may have given rise to CNEs. The heart of my concern is that both transposons and CNEs are thought to include regulatory sequences and the annotation of a sequence as a transposon not necessarily mean that it was actually derived from a transposon, but instead shares high sequence homology to a transposon. Also, given the frequency of transposon DNA in the genome, transposons often directly flank CNEs, further increasing chances that the annotation of a CNE also as a transposon can be spuriously interpreted as a transposon sequencing giving rise to a CNE. Therefore rather than stating "LINEs contribute the most CNEs" (e.g., Line 219-220), it would be better to say that "LINEs most frequently overlapped with CNE annotations"… Also, on the next line, this should be changed to "Overall, these data suggest that transposons (and LINEs in particular) may have given rise to a substantial number of reptile-specific CNEs"…

Overall, the Discussion is poorly written and lacks any actual discussion, but instead consists of multiple paragraphs that simply re-state the results. I would ask the authors to include some new more synthetic

discussion points here. Examples might include: (i) how their findings of many reptile-specific CNEs, and their possible links to transposons, open new questions about the roles of transposons that would be interesting for future studies, (ii) the relevance of their findings that repeat content may be substantially under-represented in less contiguous genome assemblies, (iii) some discussion of the variation in gene annotations across squamate genomes, and the potential that some are either massively over-counted (due to fragmentary assemblies) or undercounted due to the extreme fragmentary nature of others, based on the finding of about 14,000 genes in this highly contiguous genome and based on their Busco analyses, (iv) some broad discussion of the importance of more highly contiguous and complete assemblies for addressing sets of questions in squamates that poor genomes cannot adequately address.

Also, the author's current comment in the first discussion paragraph about the repeat content of anolis is higher in their study than a single previous study is inappropriate because the repeat content of Anolis has been re-estimated in a handful of studies since the publication of the Anolis genome (and these studies are not cited).

Text Copy Edits and Suggestions:

There are many grammatical errors in the paper, and I have tried to identify many of these below, although I would strong encourage the authors to have a very careful additional round of review to catch ones I may have missed.

Line 29 - change "with a great…" to "with great…"
Line 30 - delete "the" before "vertebrate species"
Line 40 - replace "largest" with "most abundant"
Line 41 - change "transcriptomics" to transcriptomic"
Line 63 - there are multiple issues with this sentence that could be fixed by rewriting to: Squamate reptiles (i.e., lizards and snakes) are heavily under-represented…"
Paragraph starting on line 62 - this paragraph is mostly a long list of the genomes that have been sequenced for squamates. It seems like this paragraph would be better served as a table, and more rapidly referred to in the text. Also, the point at the end that I think is meant to point out the lack of many lizard species is not made clearly and should be reworded.
Lines 87-88 - delete this last sentence because it isn't particularly important and because it doesn't totally make sense to me (i.e., why are non-native species considered endangered?)…
Line 93 - change "completeness in genes" to "completeness of genes"
Line 95 - change "annotation" to "annotations"
Line 163 - change "contained less" to "contained fewer"
Lines 187 - rewrite to avoid using "repeat modeled and repeat masked" as verbs. For example, rewrite to: "We also used repeat modeler and repeat masker to identify repeats in other reptile genomes…"

Level of Interest

Please indicate how interesting you found the manuscript: Choose an item.

Quality of Written English

Please indicate the quality of language in the manuscript: Choose an item.

Declaration of Competing Interests

Please complete a declaration of competing interests, considering the following questions:

- Have you in the past five years received reimbursements, fees, funding, or salary from an organisation that may in any way gain or lose financially from the publication of this manuscript, either now or in the future?
- Do you hold any stocks or shares in an organisation that may in any way gain or lose financially from the publication of this manuscript, either now or in the future?
- Do you hold or are you currently applying for any patents relating to the content of the manuscript?
- Have you received reimbursements, fees, funding, or salary from an organization that holds or has applied for patents relating to the content of the manuscript?
- Do you have any other financial competing interests?
- Do you have any non-financial competing interests in relation to this paper?

If you can answer no to all of the above, write 'I declare that I have no competing interests' below. If your reply is yes to any, please give details below.

I declare that I have no competing interests

I agree to the open peer review policy of the journal. I understand that my name will be included on my report to the authors and, if the manuscript is accepted for publication, my named report including any attachments I upload will be posted on the website along with the authors' responses. I agree for my report to be made available under an Open Access Creative Commons CC-BY license (http://creativecommons.org/licenses/by/4.0/). I understand that any comments which I do not wish to be included in my named report can be included as confidential comments to the editors, which will not be published.

I agree to the open peer review policy of the journal

To further support our reviewers, we have joined with Publons, where you can gain additional credit to further highlight your hard work (see: https://publons.com/journal/530/gigascience). On publication of this paper, your review will be automatically added to Publons, you can then choose whether or not to claim your Publons credit. I understand this statement. Yes
