# Supplementary material for: The genome of the tegu lizard Salvator merianae: combining Illumina, PacBio, and optical mapping data to generate a highly contiguous assembly
Source: Gigascience. 2018 Nov 27;7(12):giy141. doi: 10.1093/gigascience/giy141 (PMC6304105; doi:10.1093/gigascience/giy141)
Supplement: reviewer_3_report_(original_submission) -- Guojie Zhang — 6/26/2018 Reviewed [file giy141_reviewer_3_report_(original_submission).pdf]

Reviewer Report

Title: **The genome of the tegu lizard Salvator merianae: combining Illumina, PacBio, and optical mapping data to generate a highly contiguous assembly**

Version: **Original Submission** **Date:** 6/26/2018

Reviewer name: **Guojie Zhang**

Reviewer Comments to Author:

This manuscript by Roscito and his colleagues reported an update genome assembly of the tegu lizard Salvator merianae by hybrid assembly with data from Illumina, PacBio and Bionano. By doing so, the authors improved the genome assembly by increasing the scaffold N50 from 28Mb to55Mb and contig N50 from 175kb to 521kb, making the tegu lizard genome as the highest assembly contiguity among published reptiles genomes. Testing the BUSCO analysis for the vertebrate core gene conserved non-coding elements (CNEs) analysis showed the assembly has the highest completeness compared to other reptile genomes according to the authors' criteria. The authors also generated the multiple genome alignments of 17 species and identified conserved regions.

General comments:
Data quality: The long reads generated by Pacbio or nanopore have notorious problem for the high sequencing error rates. Many efforts have been done and published before to polish the raw sequencing reads and improve the overall quality. Still, the safest way of using pacbio long reads technology is to produce high depth of data and polish the raw reads by self-correction for systemic error. Although the short reads correction could correct the mismatch, the method has limit capacity in dealing with the indel errors for pacbio reads. The authors only produce <30x pacbio data, restricted them to use the self-correction method for polishing. The quality of the hybrid genome assembly might be a concern.

Since the authors also produced the assembly with short reads, I would recommend the authors to perform the comparison for the two genome assembly versions and produce a summary for the difference (indels, alignment quality etc.).

It would be even better if the authors could calculate the quality value for each single base of the new assembly, by using the short reads mapping results.

I could not find any assessment for the quality of the pacbio raw reads. This is also an important information for others who might be interested to use the raw reads and probably to further improve the genome by producing more pacbio reads.

Data access: all data should be uploaded online or the GigaDB. I only found the final hybrid assembly data in the website they offer. The authors should upload all the raw data and the v1 genome assembly with short reads.

Minor comments:
Abstract, I would suggest the authors to add a summary table for the data they have produced main text so the readers could easily capture what data has been used in the new genome assembly. And it would be even better if the authors could indicate the coverage for each dataset in abstract.
Line 106, the scaffold N50 should be 28.146Mb, instead of 28,146 Mb. Contig N50 should be 175.755kb.

Line 193, "protein sequences from closely-related species", how many closely-related species used, which species used? Please clarify.

Line 201, The number of genes produced by MAKER (19,101) < number of transcripts (19,128). Do you set the parameter alt_splice=1, which produce alternative splicing for each gene. I could not find it in the MAKER parameters. If so, why there are only 27 alternative splicing transcripts?
Line 380, Here you used FreeBayes to detect variants. How many homozygous variations detected? How many base errors corrected? Can provide a summary here.
Line 394 "collapsing redundant transcripts and clustering overlapping transcripts." How do you collapse and cluster the transcripts? Do you choose the transcript with longest ORF as representative from the same gene locus? I would expect the number of transcripts after collapsing and clustering to be falsely high.
Line 445, I suggest change "reading frame" to " open reading frame"
Figure 1, I suggest change "BCF error-correction" to "BCFtools error-correction" to make it clear to understand.
Figure 6, please provide citation the phylogenetic tree was taken from.
Other minor issue, the authors have used Tegu lizard genome as reference. However, the scientific interest of this species has not been well described in the introduction section. It is better to justify the argument to use this species as reference, as most other would prefer using other species like the green anole lizard as reference.

Level of Interest

Please indicate how interesting you found the manuscript: Choose an item.

Quality of Written English

Please indicate the quality of language in the manuscript: Choose an item.

Declaration of Competing Interests

Please complete a declaration of competing interests, considering the following questions:

- Have you in the past five years received reimbursements, fees, funding, or salary from an organisation that may in any way gain or lose financially from the publication of this manuscript, either now or in the future?
- Do you hold any stocks or shares in an organisation that may in any way gain or lose financially from the publication of this manuscript, either now or in the future?
- Do you hold or are you currently applying for any patents relating to the content of the manuscript?
- Have you received reimbursements, fees, funding, or salary from an organization that holds or has applied for patents relating to the content of the manuscript?
- Do you have any other financial competing interests?
- Do you have any non-financial competing interests in relation to this paper?

If you can answer no to all of the above, write 'I declare that I have no competing interests' below. If your reply is yes to any, please give details below.

I declare that I have no competing interests

I agree to the open peer review policy of the journal. I understand that my name will be included on my report to the authors and, if the manuscript is accepted for publication, my named report including any attachments I upload will be posted on the website along with the authors' responses. I agree for my report to be made available under an Open Access Creative Commons CC-BY license (http://creativecommons.org/licenses/by/4.0/). I understand that any comments which I do not wish to be included in my named report can be included as confidential comments to the editors, which will not be published.

I agree to the open peer review policy of the journal

To further support our reviewers, we have joined with Publons, where you can gain additional credit to further highlight your hard work (see: https://publons.com/journal/530/gigascience). On publication of this paper, your review will be automatically added to Publons, you can then choose whether or not to claim your Publons credit. I understand this statement. Yes
